# Medication Adherence Behavior Assessed Using a Digital Bottle Cap: Retrospective Cohort Study

**DOI:** 10.2196/91299

**Published:** 2026-09-21

**Authors:** Andrzej Nowojewski, Olga Elizarova, Annika Rutgersson, Anna Zarodkiewicz, Yunyun Jiang, Anneli Olsson, Matthew Kelham, Jessica Hanson, David Austin, Daniel A Jones, Ehsan Parvaresh Rizi, Wilhelm Ridderstråle, Jonas Oldgren, Stefan James, Robert F Storey

**Affiliations:** 1 Clinical Pharmacology and Safety Sciences Biopharmaceutical Research and Development AstraZeneca Cambridge, England United Kingdom; 2 Digital Health Oncology Research and Development AstraZeneca Waltham, MA United States; 3 Evinova AstraZeneca Mölndal Sweden; 4 Clinical Operations Biopharmaceutical Research and Development AstraZeneca Warsaw Poland; 5 Biometrics, Late-stage Development, Cardiovascular, Renal, and Metabolism, BioPharmaceuticals Research & Development AstraZeneca Gaithersburg, MD United States; 6 Division of Cardiology Department of Clinical Sciences Lund Lund University Lund Sweden; 7 Department of Cardiology Skåne University Hospital Lund Sweden; 8 Department of Cardiology St Bartholomew's Hospital London, England United Kingdom; 9 William Harvey Research Institute, Barts & The London Faculty of Medicine & Dentistry Queen Mary University of London London, England United Kingdom; 10 Cardiovascular Research Unit Division of Clinical Medicine, School of Medicine and Population Health University of Sheffield Sheffield, England United Kingdom; 11 National Institute for Health and Care Research Sheffield Biomedical Research Centre Sheffield Teaching Hospitals NHS Foundation Trust Sheffield, England United Kingdom; 12 Academic Cardiovascular Unit, The James Cook University Hospital South Tees NHS Foundation Trust Middlesbrough, England United Kingdom; 13 Population Health Science Institute Newcastle University Newcastle, England United Kingdom; 14 Late-Stage Development, Cardiovascular, Renal, and Metabolism, Biopharmaceutical Research and Development AstraZeneca Mölndal Sweden; 15 Uppsala Clinical Research Center Uppsala University Uppsala Sweden; 16 Department of Medical Sciences, Cardiology Uppsala University Uppsala Sweden

**Keywords:** acute myocardial infarction, medication adherence, adherence behavior, connected device, digital health, habit, smart bottle, remote monitoring, DAPA-MI, Dapagliflozin in Patients with Myocardial Infarction, continuous monitoring

## Abstract

**Background:**

Medication nonadherence in patients with myocardial infarction (MI) is a key obstacle to managing cardiovascular disease. However, there is a lack of studies, particularly randomized controlled trials (RCTs), focusing on objective measures of medication adherence behavior.

**Objective:**

This study aimed to (1) characterize daily medication adherence behavior and dose consistency using objective digital bottle cap data in patients post-MI enrolled in the Dapagliflozin in Patients with Myocardial Infarction (DAPA-MI) RCT, (2) identify patient and contextual factors associated with nonadherence, and (3) assess whether dose consistency is associated with adherence over time.

**Methods:**

DAPA-MI was a phase 3, double-blind, placebo-controlled RCT evaluating the effect of 10 mg of dapagliflozin versus a placebo given once daily, in addition to standard-of-care therapy, on adults hospitalized for MI. This post hoc analysis used 733,490 timepoints from a digital bottle cap that recorded when study drug bottles were opened. Adherence and dose consistency were evaluated using mixed-effects regression. Adherence was defined at the patient-day level as at least one bottle opening on a given day. Dose consistency was derived from the day-to-day variation in timing of the first recorded opening event. Digital cap data were linked with demographic, clinical, behavioral, and contextual variables to assess factors associated with nonadherence and lower dose consistency over time.

**Results:**

In total, 2429 participants were included in the analysis. Features associated with lower adherence included age <40 years (odds ratio [OR] 0.47, 99.9% CI 0.23-0.98), current smokers (OR 0.67, 99.9% CI 0.51-0.87), nonworking days, and serious adverse events. Higher adherence was associated with the New York Heart Association (NYHA) functional class III/IV (OR 1.48, 99.9% CI 1.18-1.86), low (<15 min) 7-day average time variance (OR 1.99, 99.9% CI 1.78-2.23), and morning medication use. Lower dose consistency was observed in patients aged 40-50 years (dose consistency ratio [DCR] 1.23, 99.9% CI 1.03-1.47), in current smokers (DCR 1.16, 99.9% CI 1.03-1.31), and on weekends, while patients with severe angina grading (DCR 0.83, 99.9% CI 0.74-0.94) or morning medication use (DCR 0.88, 99.9% CI 0.86-0.90) had higher dose consistency. Patients with early high dose consistency tended to maintain better adherence over time, whereas missed-dose streaks and routine disruptions were associated with worsening consistency.

**Conclusions:**

This study demonstrated the value of objective, continuous digital medication adherence behavior monitoring within a large RCT. Unlike studies relying on self-reported or prescription data, it quantified both adherence and dose consistency at scale, revealing a strong association between them. The findings indicate that temporal irregularity of medication taking may serve as a practical behavioral marker for identifying patients at increased risk of future nonadherence. These findings may advance medication adherence behavior research and inform behavior change interventions, remote monitoring strategies, and predictive adherence models.

## Introduction

Medication nonadherence in patients with myocardial infarction (MI) is considered a major barrier to managing cardiovascular disease risk factors, with 13%-61% of patients reported as nonadherent [1-3]. This can increase the risk of hospitalization, revascularization, and death [4,5], with a 3.8-fold increased risk of death in the year after a heart attack [6]. Moreover, in a randomized controlled trial (RCT) setting, nonadherence can compromise the integrity and dependability of results and may skew findings toward overestimation of dosing requirements [7].

Because of the significant impact that nonadherence can have on patients, clinical research, and health care systems [8], there are ongoing efforts to find effective interventions to improve medication adherence [9-11]. However, adherence research has primarily relied on self-reported measures of adherence [12] that can misestimate adherence due to social desirability or poor recall of missed doses [13]. Advances in remote monitoring technologies (eg, digital pill bottles and smart caps) may help overcome the weaknesses of self-reported assessments of adherence by automatically capturing medication-taking behavior, such as pill or dose timing, without depending on a person’s memory [14,15]. As a result, beyond tracking adherence itself, these devices may also be useful for assessing adherence-related factors, including habit strength [16].

A habit is an automatic behavior that develops through repeated performance in the same or similar circumstances [17]. In the case of medication adherence, this might involve taking the same number of pills at a regular time or as part of an established routine. Over time, these recurring environmental or situational cues strengthen the behavior so that it is enacted with little conscious effort and can even occur independently of deliberate intentions [18]. Because habits are characterized by consistent enactment in response to recurring cues, measures such as variation in pill-taking time may serve as a proxy for habit strength, although the relevant cues may reflect a routine time window or context rather than an exact clock time [16]. Recent research has shown that both self-reported and objective measures of habit strength are independently correlated with medication adherence [16,19].

In this study, we scrutinized the relationship between habit strength, measured as pill-timing variance (dose consistency), and medication adherence in a large cardiovascular clinical trial. Noteworthily, dose consistency is a convenient observable regularity [16] that represents only one aspect of the medication-taking habit, but habit in general will not always translate into consistency in timing [20]. Furthermore, the action of opening and closing the digital medication bottle is only a proxy for adherence, because we cannot verify independently whether the dose was actually taken [12]. Finally, the behavior of participants who know that their adherence patterns are being monitored in the context of a clinical trial may not easily translate into other contexts outside of clinical research owing to the observer effect, which tends to increase the baseline adherence [21].

The main objective of this analysis was to characterize the relationship between daily medication adherence and daily dose consistency across a longitudinal clinical trial. The secondary aim was to identify patient and contextual factors associated with nonadherence. Recent studies investigating the relationship between objective measures of habit strength and medication adherence have tended to involve few patients [14,16], use large patient databases compiled from various studies with inconsistent protocols or data collection standards [19,22], or restrict the analysis to evaluation of correlations between different measures of habit strength and adherence [16,19]. Although invaluable in establishing a link between these two variables, these studies have been unable to demonstrate a link in the context of a long clinical trial involving a large number of patients and a large number of measured confounders included in a principled regression analysis. In this work, we used 733,490 timepoints from digital bottle caps fixed to study drug bottles used by 2429 patients randomized in the Dapagliflozin in Patients with Myocardial Infarction (DAPA-MI) RCT to understand medication adherence behavior [23]. In line with European Society for Patient Adherence, Compliance, and Persistence (ESPACOMP) Medication Adherence Reporting Guideline (EMERGE) [7], this study focused on the implementation phase of adherence, defined as the extent to which patients’ dosing matched the prescribed regimen.

## Methods

### DAPA-MI Study Design

DAPA-MI was a phase 3 registry-based, multicenter, double-blind, placebo-controlled RCT conducted at 103 sites in Sweden and the United Kingdom. DAPA-MI aimed to evaluate the effect of 10 mg of dapagliflozin versus a placebo given once daily, in addition to standard-of-care therapy, on patients hospitalized for MI and with impaired left ventricular systolic function without known diabetes or established heart failure (HF). Included patients were aged ≥18 years, hospitalized for acute MI in Sweden or the United Kingdom, had evidence of regional or global impairment of left ventricular systolic function, were treated with standard therapies according to guidelines for MI management, and were enrolled in the SWEDEHEART (Sweden) or Myocardial Ischaemia National Audit Project (MINAP, UK) registries. The full methodology and the primary outcome of DAPA-MI have been published previously [23,24]. The study was registered prospectively on September 25, 2020, with ClinicalTrials.gov (NCT04564742), with the first participant enrolled on December 22, 2020.

The aim of this post hoc analysis was to characterize the daily medication adherence behavior and dose consistency of patients enrolled in DAPA-MI. The design and execution of this analysis involved no patient or public contribution.

### Study Medication Adherence Tracking

This analysis used data collected from a digital cap fixed to study drug bottles (CleverCap Lite, Compliance Meds Technologies) issued to all patients enrolled in DAPA-MI during their in-person randomization visit. The digital cap detected and recorded when the bottle was opened and closed, and it automatically uploaded this information to a central portal using a 3G wireless network, where it could be accessed by study and site teams. In line with EMERGE [7], this study focused on the implementation phase of adherence, defined as the extent to which patients’ dosing matched the prescribed regimen. Adherence during the implementation phase was operationalized as the proportion of recorded bottle openings consistent with the prescribed dosing schedule. Patients were instructed only to take the study drug once daily, without specification of the time of day. Neither the digital caps nor the mobile app (used by a subset of patients for monthly EQ-5D-5L submissions) issued notifications or reminders to patients if doses were missed. However, unexpected user patterns, including at least four consecutive missed doses, triggered a notification to the study team, who were advised to contact the patient.

### Study Population

This analysis included all patients originally enrolled into DAPA-MI, with exclusions for patients who withdrew consent, were never treated or never issued a digital cap, never used the digital cap during treatment, or used a pill organizer.

### Ethical Considerations

DAPA-MI was approved by the Swedish Ethical Review Authority (Dnr 2020-03087, 2021-03037, 2022-00101-02, and 2023-01452-02) and the UK Health Research Authority (reference #20/NW/0312). All patients received information about data collection through the digital cap and provided written informed consent to participate in the trial. Patients who did not consent to secondary analyses were excluded from this analysis. All data used in this analysis were de-identified: patient data were pseudonymized at source, and patient identities were never shared with the researchers; site names and locations were further obscured to the level of country; age was provided to the nearest year at enrollment; and GPS locations were never shared. Participants received no financial compensation for their involvement in the study other than reimbursement of reasonable expenses. No individual participant can be identified in any images of the manuscript or appendix; therefore, no additional consent was required.

### Definition of Adherence and Dose Consistency

The analysis focused on two quantitative outcome (dependent) variables: adherence and dose consistency (which is a conveniently observable proxy for habit strength). Adherence was a binary variable coded as 1 if a patient opened the digital cap at least once on a given day and 0 if not. Multiple cap openings in a single 24-hour period were labeled as “off-schedule use.”

Dose consistency on day n was defined as the absolute value of the difference in the time, t_diff_, of the first cap-opening event on day t_n_ and on the previous day |t_n_ − t_n–1_|. If the cap was not opened on day n–1, then the difference was calculated using the closest available day when an event was recorded (see Table S13 in Multimedia Appendix 1 for example calculations). The dose consistency could be calculated only on the days when the cap was opened and had to be less than 24 hours. This objective habit metric is most closely related to “standard deviation of the day-to-day hour of intake” used by Pironet et al. [19], but it uses a mean absolute deviation rather than the standard deviation because the latter can emphasize the influence of outliers.

### Variables and Data Processing

Most patient variables were collected at the baseline visit. The NYHA functional class and the Canadian Cardiovascular Society (CCS) angina grade were collected at each visit. Patients were also asked to complete the EQ-5D-5L questionnaire every month via a mobile app (Unify [25]) or the paper-based EQ-5D-3L questionnaire at in-person visits (Sweden only). For these outcomes, the most recent available value collected was used in the analyses. A full list of included variables is shown in Table S1 in Multimedia Appendix 1. Because the models used categorical variables, covariates with less than 10% of missing values were imputed using the mode (ie, most common level). Variables with higher proportions of missing values were excluded. We used Jamshidian and Jalal’s nonparametric test to evaluate whether the values were missing completely at random (MCAR) [26], because most of the variables were not normally distributed.

Further refinements to generate the “adherence dataset” included removal of data points outside the DAPA-MI treatment period (ie, date of first dose to date of last dose) from concurrent devices and from superfluous digital cap events. On days when a patient opened the digital cap multiple times, the first event was kept in the dataset and additional events labelled as “off-schedule use,” which was included as an explanatory variable. The “dose consistency” dataset included only data points for which there were no missing events (ie, for which dose consistency, |t_n_ – t_n–1_|, could be calculated).

### Statistical Analysis

Univariate comparisons of included and excluded patient populations in the analysis were performed using the chi-squared test for categorical variables, the two-sample *t* test for continuous variables that had approximately normal distributions, and the Kruskal-Wallis test for continuous variables with nonnormal distributions.

To identify factors driving medication adherence and dose consistency, a mixed-effects modeling approach accounted for the repeated measures design of within-patient data points grouped within site. Two levels of random intercept were used, patient and site, which were coded to be explicitly nested because patients were not permitted to participate in multiple studies at the same time. The adherence model was a logistic mixed-effects regression model with a binary adherence indicator for each patient-day and with the fixed-effects listed in Table S1 in Multimedia Appendix 1.

Because dose consistency was quantified as a time interval with a nonnormal distribution, two separate transformations were used to normalize it: (1) a logarithmic transformation, y_n_ = ln(|t_n_ – t_n–1_| + 1 second), which implies a straightforward multiplicative interpretation of the model coefficients, and (2) a Box–Cox transformation, which enables fulfilment of the modeling assumptions of normality and homoscedasticity. Both transformed variables were then used in linear mixed-effects regression models, with the fixed effects listed in Table S1 in Multimedia Appendix 1.

All except two independent variables overlapped between the adherence and dose consistency models. “Average time variance in the past 7 days” was used as an independent variable in the adherence model to test the relationship between average dose consistency and adherence. For the same reason, the dose consistency model included the “after a missed dose” variable that quantified proximity to short or long nonadherence streaks. In some cases, data collection via the digital cap was temporarily suspended by the physician, usually owing to a patient holiday; these periods were defined as “data gaps.”

Analyses were performed using Python version 3.9.5 [27], R version 4.1.0 (R Foundation for Statistical Computing) [28], and the glmmTMB 1.1.9 library in R [29]. Public holidays were calculated using the workalendar 16.4.0 library, and statistical models were fitted using the glmmTMB 1.1.9 library in R. We used the MCAR test implementation found in the mice 3.19.0 package in R [30].

Additional analyses (as reported in Multimedia Appendix 1) included adherence and dose consistency according to EQ-5D-3L/EQ-5D-5L anxiety responses, a comparison of patients who established early high consistency versus those with early lower consistency, and a sensitivity analysis for the dose consistency model using Box–Cox-transformed data.

## Results

### Patient Characteristics

Of 4017 patients randomized in DAPA-MI, 2429 (60.5%) were included in this analysis (Figure 1). After dataset refinement, 733,490 patient-days were included in the adherence dataset and 682,731 patient-days of cap-opening events (one event per day) in the dose consistency dataset (Figure S1 in Multimedia Appendix 1). Patient characteristics are summarized in Table 1. The Anderson-Darling rank test statistic was 4.3 and median *P*=.15 for the Jamshidian and Jalal’s nonparametric MCAR test [26], showing insufficient evidence to reject MCAR at the .05 significance level. Univariate comparisons identified differences between included and excluded patients in country, race, height, NYHA functional class and CCS angina grade at the final assessment, and number of missing cap-opening events (eg, due to use of pill organizers), but otherwise, the groups were comparable.

**Figure 1:**
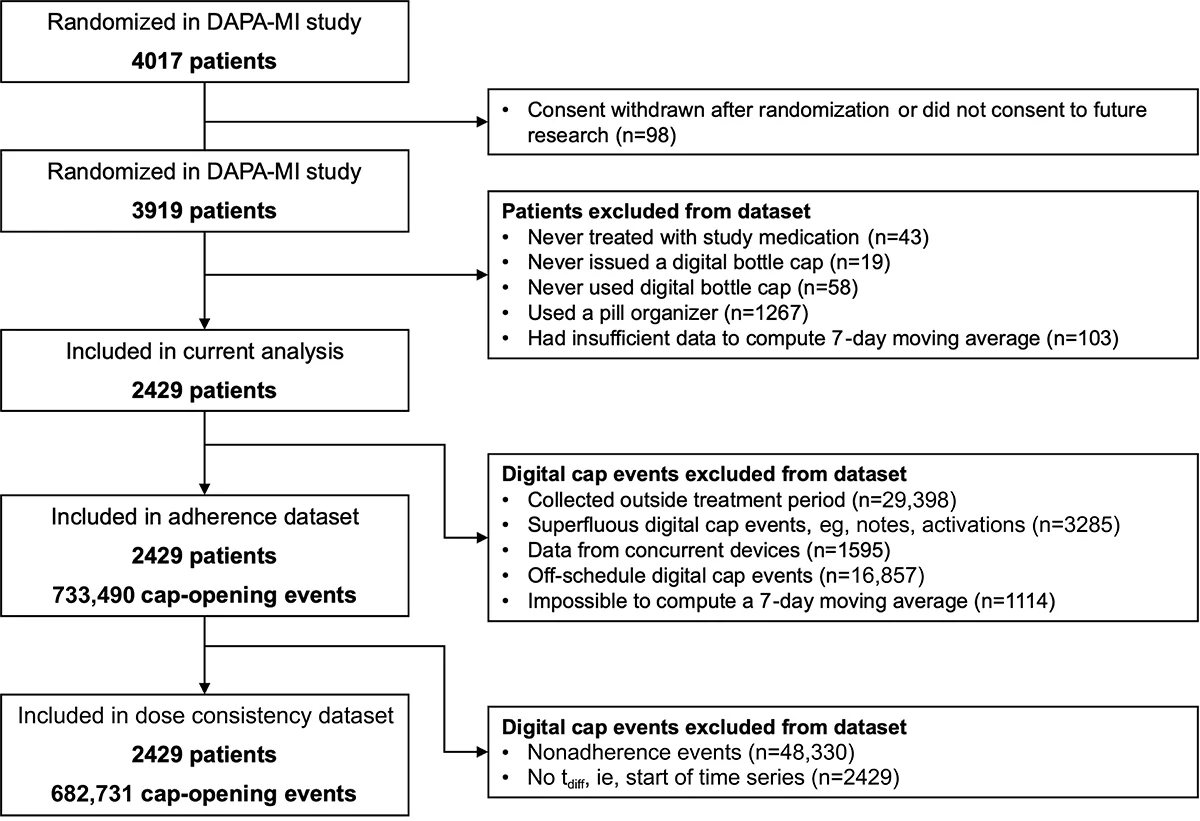
Flow chart showing selection of the analysis population from the DAPA-MI trial and derivation of the digital cap datasets used for adherence and dose consistency analyses. Of 4017 randomized patients, 2429 were included after excluding those who withdrew consent, were never treated, were not issued or did not use a digital cap during treatment, used a pill organizer, or did not consent to secondary analyses. After data cleaning, including removal of records outside the treatment period, from concurrent devices, and superfluous cap events, the adherence dataset comprised 733,490 patient-days and the dose consistency dataset comprised 682,731 cap-opening events. Dose consistency (t_diff_) was based on the absolute difference in timing between cap-opening events on consecutive days. DAPA-MI: Dapagliflozin in Patients with Myocardial Infarction; t_diff_: time difference between cap-opening events on consecutive days.

**Table 1:** Characteristics of the overall study population and comparison of patients included versus excluded from the analysis.^a^

Characteristics		Missing patients, n (%)	All patients (N=3919)	Patients excluded from analysis (n=1490)	Patients included in analysis (n=2429)	*P* value^b^
**Country, n (%)**						
	United Kingdom	0	2766 (70.6)	871 (58.5)	1895 (78.0)	<.001^c^
	Sweden	—	1153 (29.4)	619 (41.5)	534 (22.0)	—^d^
**Sex, n (%)**						
	Female	0	779 (19.9)	295 (19.8)	484 (19.9)	.96^c^
	Male	—	3140 (80.1)	1195 (80.2)	1945 (80.1)	—
**Race, n (%)**						
	Asian	3 (0.1)	113 (2.9)	36 (2.4)	77 (3.2)	<.001^c^
	Other	—	98 (2.5)	62 (4.2)	36 (1.5)	—
	White	—	3705 (94.6)	1391 (93.4)	2314 (95.3)	—
**Study arm, n (%)**						
	Dapagliflozin (10 mg)	0	1960 (50.0)	759 (50.9)	1201 (49.4)	.38^c^
	Placebo	—	1959 (50.0)	731 (49.1)	1228 (50.6)	—
Age, mean (SD)		0	62.9 (10.8)	62.3 (11.3)	63.2 (10.6)	.02^e^
Height, mean (SD)		21 (0.5)	173.9 (9.0)	174.7 (9.0)	173.4 (9.0)	<.001^e^
Weight, mean (SD)		10 (0.3)	85.6 (16.2)	86.7 (16.7)	85.0 (15.9)	.002^e^
BMI, mean (SD)		25 (0.6)	28.3 (4.8)	28.3 (4.9)	28.2 (4.8)	.45^e^
**Smoking status, n (%)**						
	Current	7 (0.2)	1086 (27.8)	391 (26.3)	695 (28.6)	.09^c^
	Former	—	1158 (29.6)	468 (31.5)	690 (28.4)	—
	Never	—	1668 (42.6)	625 (42.1)	1043 (43.0)	—
**NYHA^f^** **functional class, n (%)^g^**						
	No disease	71 (1.8)	1945 (50.5)	814 (56.2)	1131 (47.1)	<.001^c^
	I	—	1266 (32.9)	416 (28.7)	850 (35.4)	—
	II	—	529 (13.7)	184 (12.7)	345 (14.4)	—
	III/IV^h^	—	108 (2.8)	34 (2.3)	74 (3.1)	—
**Angina grade, n (%)^g^**						
	No disease	75 (1.9)	2987 (76.2)	1151 (77.2)	1836 (75.6)	<.001^c^
	CCS^i^ I	—	667 (17.0)	219 (14.7)	448 (18.4)	—
	CCS II	—	100 (2.6)	39 (2.6)	61 (2.5)	—
	CCS III/IV^h^	—	32 (0.8)	8 (0.5)	24 (1.0)	—
	Atypical chest pain	—	58 (1.5)	26 (1.7)	32 (1.3)	—
**Left ventricular ejection fraction, n (%)**						
	30-49	226 (5.8)	2610 (70.7)	1014 (71.0)	1596 (70.5)	.83^c^
	<30	—	260 (7.0)	96 (6.7)	164 (7.2)	—
	≥50	—	823 (22.3)	318 (22.3)	505 (22.3)	—
Treatment length (days), median (IQR)		71 (1.8)	316.0 (160.8-475.2)	332.0 (167.0-493.0)	308.0 (158.0-464.0)	.13^j^
Number of cap-opening events, median (IQR)		0	301.0 (154.0-462.0)	295.5 (138.0-465.0)	303.0 (161.0-457.0)	.02^j^
Number of missed cap-opening events, median (IQR)		0	29.0 (9.0-103.0)	129.0 (47.0-274.0)	15.0 (6.0-35.0)	<.001^j^
**Outcome: death, n (%)**						
	No	0	3848 (98.2)	1456 (97.7)	2392 (98.5)	.11^c^
	Yes	—	71 (1.8)	34 (2.3)	37 (1.5)	—
**Outcome: diabetes onset, n (%)**						
	No	0	3766 (96.1)	1422 (95.4)	2344 (96.5)	.11^c^
	Yes	—	153 (3.9)	68 (4.6)	85 (3.5)	—
**Outcome: >5% weight loss, n (%)**						
	No	0	3030 (77.3)	1181 (79.3)	1849 (76.1)	.03^c^
	Yes	—	889 (22.7)	309 (20.7)	580 (23.9)	—

^a^All characteristics were recorded at baseline, unless otherwise specified. A high proportion of patients excluded from analysis were users of pill organizers (n=1267, 32.3%; see Figure 1 for details).

^b^*P* values are from univariate comparisons between included and excluded groups.

^c^Chi-squared test for categorical variables.

^d^Not applicable.

^e^Two-sample *t* test for approximately normally distributed continuous variables.

^f^NYHA: New York Heart Association.

^g^The NYHA functional class and CCS angina grade were measured several times during the study; analyses were conducted using the most recent value collected.

^h^Given the small number of patients with class III/IV disease, these two categories were merged.

^i^CCS: Canadian Cardiovascular Society.

^j^Kruskal-Wallis test for nonnormally distributed continuous variables.

### Overall Adherence and Dose Consistency

Among the 2429 patients, overall adherence (median [IQR] of average adherence values for each included patient) was 96.7% (91.4%-98.9%). These findings represent implementation phase adherence outcomes according to the EMERGE framework [7], as measured by recorded bottle openings compared with the prescribed dosing regimen. The median time difference in adherence on consecutive days (dose consistency) was 34.7 (IQR 11.8-83.3) minutes (Table S2 in Multimedia Appendix 1).

There was a wide variety of behavioral adherence patterns exhibited by patients in terms of adherence and timing (Figure 2).

**Figure 2:**
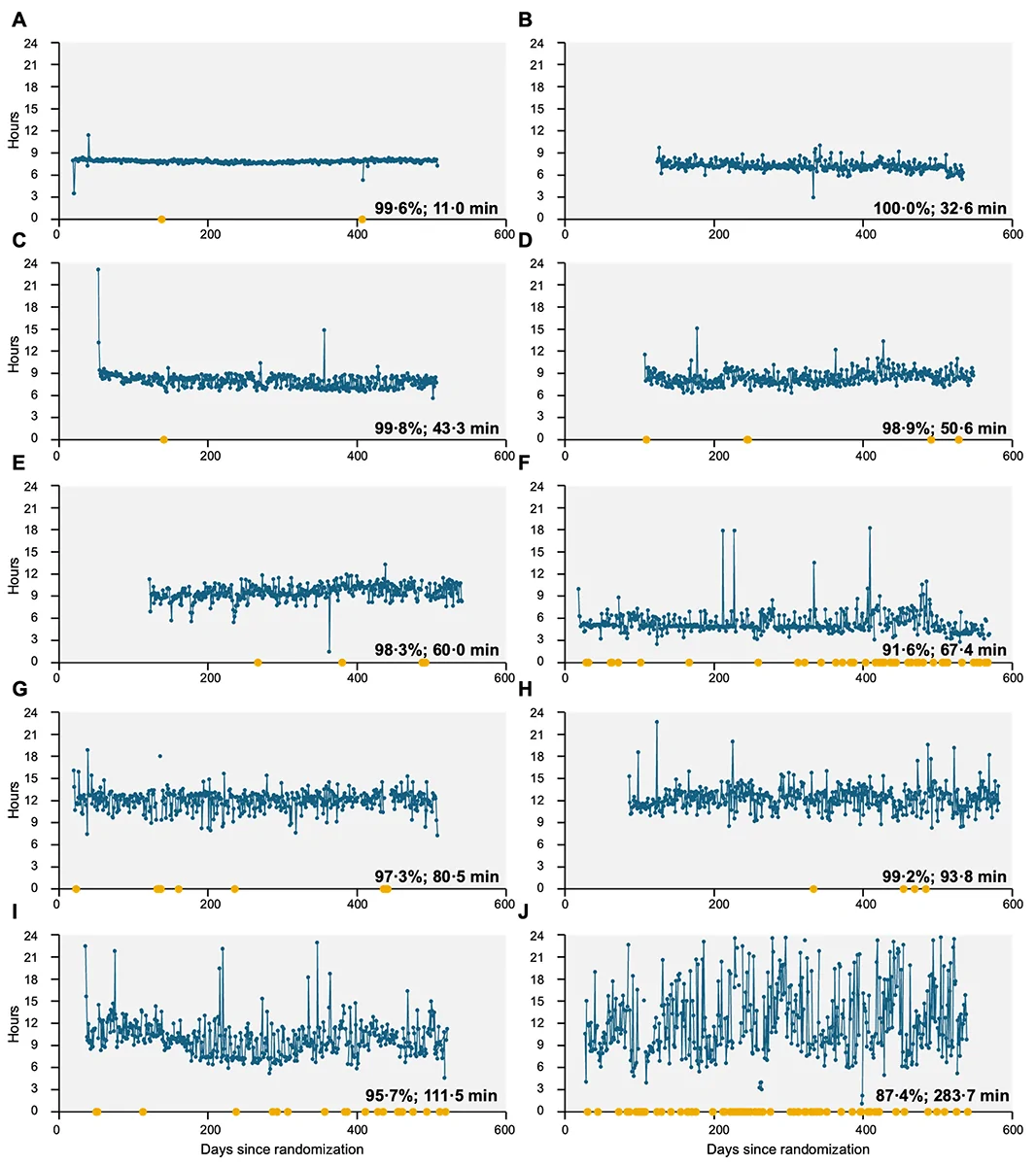
Illustrative plots showing individual digital cap–recorded medication-taking patterns over time in selected patients from the DAPA-MI trial. Patients with 400-500 days of trial participation (to make the traces more visually comparable) were ranked by average dose consistency, divided into deciles, and one patient was selected from each decile for display. Blue dots indicate cap-opening events, with consecutive events connected by blue lines; yellow dots on the horizontal axis indicate days with no recorded cap opening. The percentage shown in the bottom-right corner of each panel indicates overall adherence for the patient whose trace is shown, and the value in minutes indicates the mean deviation in timing of cap-opening events on consecutive days. These plots illustrate the heterogeneity of adherence behavior and timing consistency across patients. DAPA-MI: Dapagliflozin in Patients with Myocardial Infarction.

### Adherence Model

Figure 3 shows the results of the logistic regression model for adherence (full results in Table S3 in Multimedia Appendix 1; Figure S2 and the corresponding Table S4 in Multimedia Appendix 1 show acceptable distribution of random effects). Based on this, numerous factors contributed to adherence throughout the trial. Younger patients (aged <40 years) were 53% less likely (odds ratio [OR] 0.47, 99.9% CI 0.23-0.98) to be adherent than older patients (aged 60-70 years). Current smokers were 33% less likely (OR 0.67, 99.9% CI 0.51-0.87) to be adherent than patients who had never smoked. Adherence decreased gradually in the first 120 days of participation and then remained consistent until the end of the treatment. Adherence was likely to be higher in patients with an NYHA classification of “marked” or “severe” HF symptoms (class III/IV; OR 1.48, 99.9% CI 1.18-1.86) than in those without HF symptoms.

Patients were less likely to be adherent by 16%, 14%, 9%, and 34% on Saturdays (OR 0.84, 99.9% CI 0.79-0.90), Sundays (OR 0.86, 99.9% CI 0.80-0.91), public holidays (OR 0.91, 99.9% CI 0.83-0.99), and after commencement of study close-out visits (from February 27, 2023; OR 0.66, 99.9% CI 0.58-0.73), respectively. Compared with patients who used the smart bottle between 0800 and 1200 hours (reference group), patients who tended to use the device between 0400 and 0800 hours were more likely to be adherent (OR 1.08, 99.9% CI 1.01- 1.14), while patients who used it late in the evening (between 2000 and 0000 hours) were less likely to be adherent (OR 0.59, 99.9% CI 0.52-0.68). Patients who opened the smart bottle more than once a day in the preceding 7 days were significantly less likely to be adherent (OR 0.54, 99.9% CI 0.51-0.56) than patients who had no off-schedule use in the past 7 days. Patients were more likely to be adherent in the 7 days after completing the EQ-5D-5L questionnaire via the mobile app (OR 1.21, 99.9% CI 1.06-1.36) than on days with no app use or more than 7 days after the most recent app use. They were also more likely to be adherent when COVID-19 restrictions were in place at the site location (OR 1.18, 99.9% CI 1.05-1.32) than when no restrictions were in place.

Patients experiencing serious adverse events (AEs) that required both hospitalization and drug discontinuation, hospitalization only, discontinuation only, or neither were 91% (OR 0.09, 99.9% CI 0.07-0.11), 75% (OR 0.25, 99.9% CI 0.20-0.30), 92% (OR 0.08, 99.9% CI 0.05-0.11), and 35% (OR 0.65, 99.9% CI 0.53-0.78), respectively, less likely to be adherent than patients without AEs. In the 7 days after a serious AE, patients were 78% (OR 0.22, 99.9% CI 0.15-0.33), 27% (OR 0.73, 99.9% CI 0.54-0.97), and 91% (OR 0.09, 99.9% CI 0.04-0.20) less likely to be adherent in the first three instances, respectively.

A 7-day moving average of the time variance in cap-opening events (ie, weekly adherence) was strongly associated with adherence, with patients with the lowest time variance (<15 minutes) being almost twice as likely to be adherent as patients with a 7-day variance of >2 hours (OR 1.99, 99.9% CI 1.78-2.23).

**Figure 3:**
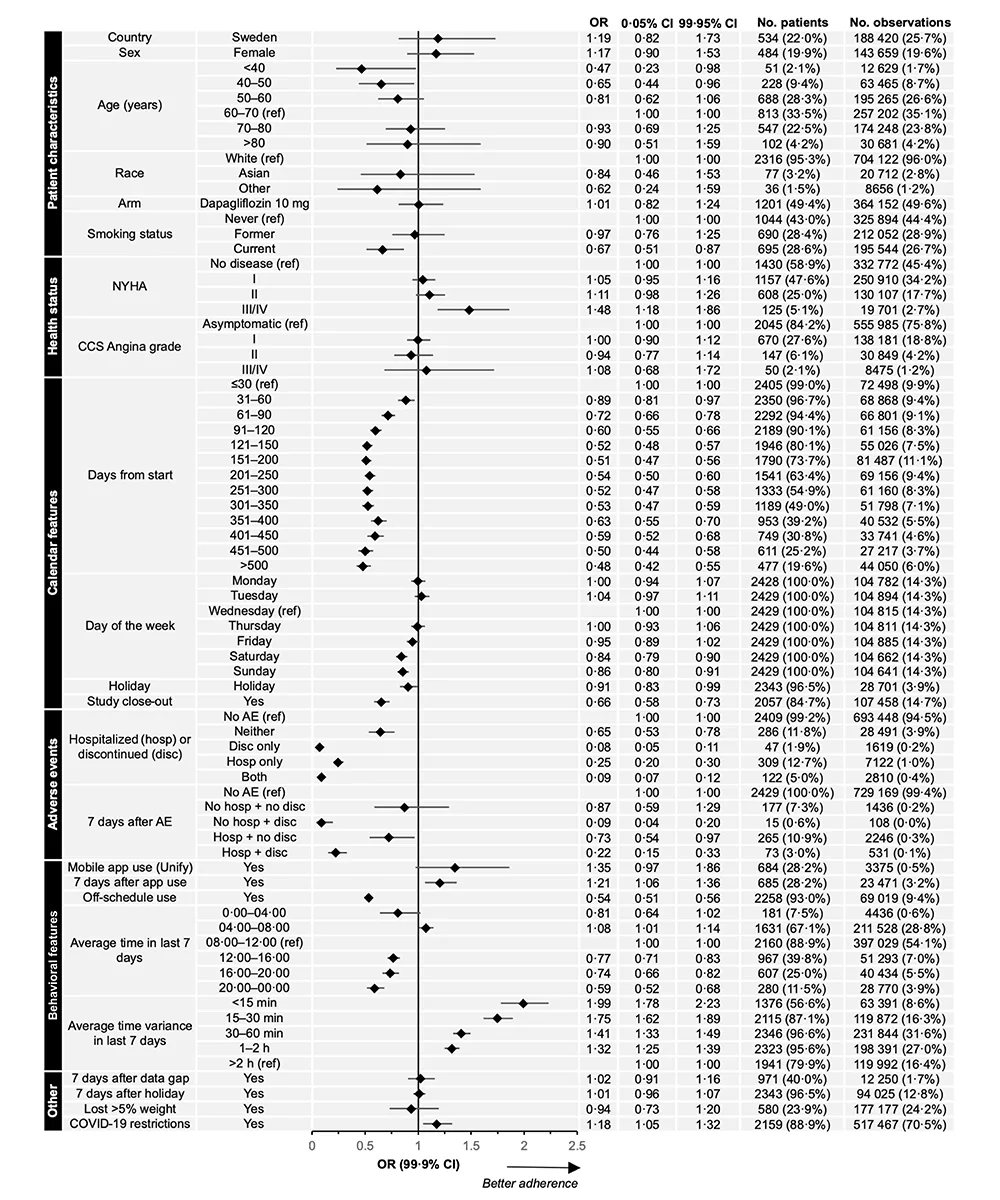
Forest plot showing results from the logistic mixed-effects regression model for daily adherence during the treatment period. Data are presented as ORs (99.9% CIs) for factors (full list available in Table S1 in Multimedia Appendix 1) associated with the likelihood of adherence, defined as at least one digital cap opening on a given day. Full model results are provided in Table S3 in Multimedia Appendix 1. AE: adverse event; CI: confidence interval; CCS: Canadian Cardiovascular Society; NYHA: New York Heart Association; OR: odds ratio.

### Dose Consistency Model

Dose consistency was analyzed using a linear regression model, and the results are shown in Figure 4 (full results in Table S5 in Multimedia Appendix 1; Figure S3C,D in Multimedia Appendix 1 shows no significant heteroscedasticity and an acceptable quantile-quantile plot of residuals, while Figure S2C,D and the corresponding Table S4 in Multimedia Appendix 1 show acceptable distribution of random effects). Compared with patients aged 60-70 years (reference population), patients aged 40-50 years had a 23% lower dose consistency (dose consistency ratio [DCR] 1.23, 99.9% CI 1.03-1.47), and patients aged >80 years had a 24% higher dose consistency (DCR 0.76, 99.9% CI 0.59-0.98). Current smokers had a 16% lower dose consistency than patients who never smoked (DCR 1.16, 99.9% CI 1.03-1.31), and patients who had angina symptoms at rest or during ordinary physical activity (CCS angina grade III/IV) had a 17% higher dose consistency (DCR 0.83, 99.9% CI 0.74-0.94). Dose consistency decreased gradually throughout the study, decreasing by 19% between days 450 and 500 compared with the first 30 days after treatment start (DCR 1.19, 99.9% CI 1.14-1.24).

Dose consistency was significantly lower on the weekends than on Wednesdays (reference: Saturdays DCR 1.47, 99.9% CI 1.44-1.50; Sundays DCR 1.32, 99.9% CI 1.29-1.35). Dose consistency was also moderately lower on Tuesdays (DCR 1.06, 99.9% CI 1.04-1.08) and Fridays (DCR 1.07, 99.9% CI 1.04-1.09) than on other days. Patients who used the digital smart bottle between 0400 and 0800 hours in the preceding 7 days had a higher dose consistency (DCR 0.88, 99.9% CI 0.86-0.90) than patients who used the bottle between 0800 and 1200 hours, and patients who used the bottle in the afternoon tended to have a lower dose consistency (DCR 1.41, 99.9% CI 1.37-1.46). Dose consistency also decreased after the following disruptions: by 9% on the day of mobile app use (DCR 1.09, 99.9% CI 1.01-1.19), by 10% in the 7 days after off-schedule use (DCR 1.10, 99.9% CI 1.08-1.12), by 38% in the 7 days after a data gap (DCR 1.38, 99.9% CI 1.32-1.44), and by 4% in the 7 days after a public holiday (DCR 1.04, 99.9% CI 1.03-1.06).

The dose consistency model distinguished the days after a long streak of missed doses (>3 days, n=720 events) versus shorter streaks (≤3 days, n=2115 events). The first day after a long streak was associated with a significantly lower dose consistency than a short streak (DCR 2.18, 99.9% CI 1.95-2.43, vs DCR 1.59, 99.9% CI 1.54-1.64) compared with days without a recent history of missed doses.

Finally, the presence of serious AEs decreased dose consistency in cases requiring both hospitalization and study medication discontinuation (DCR 1.20, 99.9% CI 1.04-1.38), hospitalization only (DCR 1.23, 99.9% CI 1.14-1.33), or neither (DCR 1.15, 99.9% CI 1.09-1.22). These effects persisted during an AE but not in the days immediately after it. In cases when an AE led to a recommendation to discontinue study medication without hospitalization, there were insufficient data points for the effect to be statistically significant.

**Figure 4:**
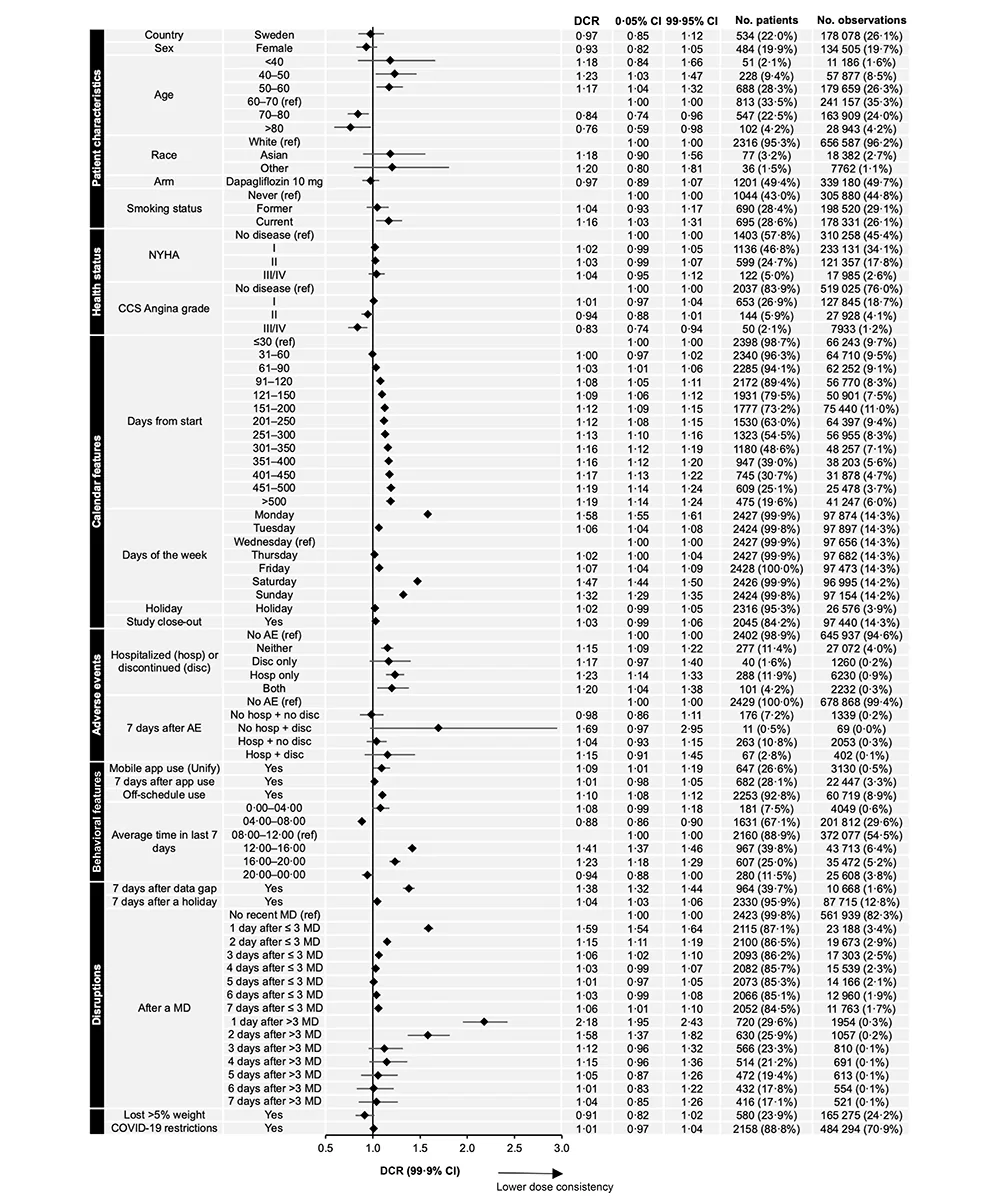
Forest plot showing results from the linear mixed-effects regression model for dose consistency during the treatment period. Data are presented as exponentiated coefficients, reported as DCRs (99.9% CIs) from the model fitted to log-transformed dose consistency values. Dose consistency was defined as the absolute difference in timing between cap-opening events on consecutive days. DCRs>1 indicate lower dose consistency relative to the reference category, corresponding to greater variability in medication timing, whereas DCRs<1 indicate higher dose consistency, corresponding to more consistent timing of cap-opening events. Full model results are provided in Table S5 in Multimedia Appendix 1. AE: adverse event; CCS: Canadian Cardiovascular Society; CI, confidence interval; DCR: dose consistency ratio; MD, missed dose; NYHA: New York Heart Association; OR: odds ratio.

### Additional Analyses

Results of the sensitivity analysis for the dose consistency model using Box–Cox-transformed data are shown in Figure S4 and Table S6 in Multimedia Appendix 1. Patients with the highest anxiety level according to EQ-5D-3L/EQ-5D-5L had significantly higher adherence than patients with no anxiety (Figures S5 and S6 and Tables S7 and S8 in Multimedia Appendix 1). Compared with patients with early low dose consistency, patients who established early high dose consistency continued to exhibit high dose consistency until the end of the study and had significantly higher adherence for up to a year into the study (Figures S7 and S8 and Tables S9-S12 in Multimedia Appendix 1).

## Discussion

### Principal Findings

This was a comprehensive post hoc analysis of medication adherence using a digital bottle cap in 2429 patients with a recent acute MI enrolled in a multicenter, double-blind RCT in two countries, covering ~700,000 cap-opening events. A key finding of this analysis was the identification of a relationship between medication adherence and dose consistency. Adherence was almost twice as high in patients with higher 7-day dose consistency than in those with lower dose consistency. Dose consistency also decreased after a streak of missed doses and further decreased when patients had more than three missed doses compared with shorter nonadherence streaks. These findings are largely in line with previous research [14,16,19]. Moreover, patients with low initial dose consistency were more likely to have lower adherence for the remainder of the trial than patients with early high dose consistency. A possible explanation for this may be that patients who took their medication at a similar time every day had some form of external reminder system. A previous study also identified a link between irregular schedules and lower adherence to medication amongst shift workers [31].

The analysis identified certain patient features associated with nonadherence and dose consistency. In keeping with previous studies in patients with cardiovascular disease and hypertension [32,33], younger patients had lower adherence and lower dose consistency than older patients. One reason for this may be that older patients are more likely to have caregivers, which has been linked to higher adherence [34]. Compared with patients with no disease/symptoms according to the NYHA functional class or the CCS angina grade, those with the most severe NYHA classifications were 50% more likely to be adherent to medication, and those with the most severe CCS angina grades had significantly higher dose consistency [35]. Other features associated with lower adherence included current smokers (vs patients who had never smoked) [36], lower anxiety levels [37], and absence of COVID-19 restrictions. Although depression has consistently shown a negative relationship with medication adherence, the evidence for anxiety has been more mixed [37,38]. In post-MI settings, mild anxiety may increase illness salience and vigilance toward preventive behaviors. Our use of a single anxiety/depression item from EQ-5D-3L/EQ-5D-5L may have captured health-focused anxiety, which could explain the higher adherence and dose consistency observed in these patients. Together, these observations suggest that a patient’s state of mind and understanding of the seriousness of their condition affect their adherence and dose consistency behaviors [39].

Serious AEs led to a significant decrease in adherence and decrease in dose consistency, likely owing to the hospitalization of patients, the study physician recommending a pause in treatment (the DAPA-MI protocol stipulated temporary interruption of dapagliflozin after certain AEs), or disruption to patient’s daily routines [40]. Although dose consistency returned to normal after AE resolution, adherence did not.

Medication timing also affected both adherence and dose consistency, with patients who took their medication between 0400 and 0800 hours being more likely to be adherent and have higher dose consistency than patients who took their medication at other times. These findings are in keeping with a previous study that identified similar trends in relation to medication timing [22]. Higher adherence and dose consistency in patients who took medication in the morning compared with the evening may be explained by the fact that mornings tend to vary less for most people and therefore allow easier adherence to a routine [41]. This may also explain why other routine-interrupting disruptions, such as weekends, off-schedule use, and data gaps, led to lower adherence and dose consistency. Adherence also increased after use of the mobile app to complete the EQ-5D-5L questionnaire, although dose consistency decreased on the day of app use [42]. It is possible that an app notification reminded patients to take dapagliflozin, which may have occurred outside their usual routine.

### Limitations

The primary limitation of this analysis is the assumption that opening a medication bottle is equivalent to ingesting a single unit of the drug, which is not always valid [43]. Similarly, a patient recorded as opening the bottle multiple times in a day (recorded as “off-schedule use” in our analysis) does not necessarily imply ingestion of multiple doses, as patients have been observed to open medication bottles out of curiosity [44]. Furthermore, the conclusions of this analysis have limited generalizability to real-world settings and other countries because the RCT only included patients from two countries who were aware their medication adherence was being monitored, which is likely to have affected their behavior [45]. Importantly, interventions implemented by site teams in response to smart bottle activity were not consistently recorded as part of the trial, and the type and timing of intervention differed between sites. For example, the study teams encouraged physicians to contact patients if the medication bottle was not opened for several consecutive days, but this policy was not consistent through time or across study sites. Other limitations are the exclusion of data from 32% of the DAPA-MI population because of the use of pill organizers and the lack of information about psychological variables on patients’ attitudes to medication. Furthermore, dose consistency represents an objective proxy for habit strength rather than pure automaticity, and the observed late-phase decline in dose consistency may reflect contextual drift or shifting daily cues rather than true habit weakening [19,41]. Finally, it is possible that in some instances, technological issues resulted in false recording of missing data (eg, the digital cap battery running out or the lack of a network connection).

### Conclusion

This study provided a novel and highly granular characterization of medication adherence behavior in patients with a recent acute MI by leveraging continuous, objective digital bottle cap monitoring within a large RCT. Unlike most previous medication adherence studies, which have relied primarily on self-reported measures, prescription refill data, or smaller observational cohorts [14,16], this analysis captured more than 700,000 real-world medication-taking events and simultaneously evaluated both adherence and dose consistency over time. By demonstrating a robust relationship between dose consistency and adherence, and by identifying specific patient-, behavioral-, and contextual-level predictors of both outcomes (in particular patient age, smoking status, HF symptom severity, daily medication timing, schedule disruptions, and AEs), the findings advance our understanding of the mechanisms underlying persistence on a medication and contribute important evidence to the emerging field of using advanced digital technologies to enable more objective and granular adherence monitoring and identifying patients at risk of nonadherence. In practice, the results might have direct implications for the design of behavior change interventions, remote monitoring strategies, and predictive machine learning tools that could enable earlier detection of adherence deterioration and more relevant and timely support for patients with cardiovascular disease and other chronic conditions.

## Appendix

Multimedia Appendix 1 Supplementary methods, results, figures, and tables. The appendix includes model specifications, sensitivity analyses, subgroup analyses, diagnostic plots, and detailed regression results.

## Abbreviations

AE: adverse event
CCS: Canadian Cardiovascular Society
DAPA-MI: Dapagliflozin in Patients with Myocardial Infarction
DCR: dose consistency ratio
EMERGE: European Society for Patient Adherence, Compliance, and Persistence (ESPACOMP) Medication Adherence Reporting Guideline
HF: heart failure
MCAR: missing completely at random
MI: myocardial infraction
NYHA: New York Heart Association
OR: odds ratio
RCT: randomized clinical trial

## Data Availability

Data underlying the findings described in this manuscript may be obtained in accordance with AstraZeneca’s data-sharing policy [46]. Data for studies directly listed on Vivli [47] and not listed on Vivli [48] could be requested through Vivli. AstraZeneca Vivli member page is also available outlining further details [49].
