# Supplementary material for: Medication Adherence Behavior Assessed Using a Digital Bottle Cap: Retrospective Cohort Study
Source: J Med Internet Res. 2026 Sep 21;28:e91299. doi: 10.2196/91299 (PMC13639418; doi:10.2196/91299)
Supplement: Supplementary file 1 [file jmir_v28i1e91299_app1.docx]

**Medication Adherence Behavior Assessed Using a Digital Bottle Cap: Retrospective Cohort Study**

**Table of Contents**

[Supplementary methods and results	3](#_Toc239345070)

[1. Study design	3](#_Toc239345071)

[2. Dose consistency modelling approach	3](#_Toc239345072)

[3. Sensitivity analysis	3](#_Toc239345073)

[4. EQ-5D anxiety effect	3](#_Toc239345074)

[5. Comparison of patients with higher versus lower initial dose consistency	3](#_Toc239345075)

[Supplementary Figure 1. Distribution of all adherence times in the dose consistency dataset	5](#_Toc239345076)

[Supplementary Figure 2. Random effects distribution	6](#_Toc239345077)

[Supplementary Figure 3. Residual distribution diagnostic plots for linear regression models	7](#_Toc239345078)

[Supplementary Figure 4. Box–Cox-transformed dose consistency model forest plot	8](#_Toc239345079)

[Supplementary Figure 5. Adherence model forest plot for the anxiety subset	9](#_Toc239345080)

[Supplementary Figure 6. Box–Cox-transformed dose consistency model forest plot for the anxiety subset	10](#_Toc239345081)

[Supplementary Figure 7. Adherence model forest plots for patients with low and high initial dose consistency	11](#_Toc239345082)

[Supplementary Figure 8. Box–Cox-transformed dose consistency model forest plots for patients with low and high initial dose consistency	12](#_Toc239345083)

[Supplementary Table 1. Independent variables used in adherence and dose consistency models	13](#_Toc239345084)

[Supplementary Table 2. Summary of adherence and dose consistency datasets	15](#_Toc239345085)

[Supplementary Table 3. Results of adherence model	21](#_Toc239345086)

[Supplementary Table 4. Summary statistics for patient and site random effects for adherence, log-transformed and Box–Cox-transformed dose consistency models	26](#_Toc239345087)

[Supplementary Table 5. Results of log-transformed dose consistency	27](#_Toc239345088)

[Supplementary Table 6. Results of Box–Cox-transformed dose consistency model	34](#_Toc239345089)

[Supplementary Table 7. Results of adherence model for the anxiety subset	38](#_Toc239345090)

[Supplementary Table 8. Results of Box–Cox-transformed dose consistency for the anxiety subset	42](#_Toc239345091)

[Supplementary Table 9. Results of adherence model for patients with low initial dose consistency	46](#_Toc239345092)

[Supplementary Table 10. Results of adherence model for patients with high initial dose consistency	50](#_Toc239345093)

[Supplementary Table 11. Results of Box–Cox-transformed dose consistency model for patients with low initial dose consistency	54](#_Toc239345094)

[Supplementary Table 12. Results of Box–Cox-transformed dose consistency model for patients with high initial dose consistency	58](#_Toc239345095)

[Supplementary Table 13. Example of calculation of average time variance in last 7 days.	62](#_Toc239345096)

[Supplementary References	62](#_Toc239345097)

## Supplementary methods and results

#### 1. Study design

Race, smoking status, hypertension history, body mass index (BMI), estimated glomerular filtration rate (eGFR), ST-elevation myocardial infarction (STEMI), and New York Heart Association (NYHA) and Canadian Cardiovascular Society (CCS) Angina Grade variables each had fewer than 1.5% of missing values, whereas the left ventricular ejection fraction (LVEF) missingness rate was 6.8%. These missing values were replaced using the most frequent level for each variable (mode imputation). Because the EQ-5D questionnaire was optional, there were over 50% of missing values and therefore EQ-5D results were not included in the main analysis; instead, the same models were fitted on a subset dataset including only the patients with an EQ-5D anxiety response.

#### 2. Dose consistency modelling approach

To justify our dose consistency modelling approach, **Supplementary Figure 1A** shows the distribution of all adherence times in the dose consistency dataset showing an overwhelming preference for taking the medication close to 08:00. **Supplementary Figure 1B** shows the distribution of dose consistency (the pill-timing variance on consecutive days: |t_n_-t_n-1_|) truncated at 12 h, which is calculated using the approach described in the Methods section by taking an absolute value of the difference in adherence times on consecutive days. The distribution has a significant right skew. The full distribution is shown in **Supplementary Figure 1C** using a logarithmic scale to highlight the structure of the tail of the distribution close to 24 h. These distributions are more easily modelled once transformed and **Supplementary Figure 1D** and 1**E** show the result of logarithmic and a Box–Cox transformation (with λ=0.15). The discontinuity in the lower range is because adherence timing data was recorded to the nearest second. Both transformations brought the overall distribution closer to the Gaussian distribution which is shown in **Supplementary Figure 1E** with a red outline.

#### 3. Sensitivity analysis

The results described above are based on a linear regression model fitted to a log-transformed dose consistency data. Although the distribution of random effects (**Supplementary Figure 2C and** **2D**) is acceptable and **Supplementary** **Figure** **3D** shows no heteroscedasticity in the residuals, the quantile–quantile plot in **Supplementary** **Figure** **3C** suggests that the fit may not be perfect. While past research^1^ has shown that this would have negligible effect on central estimates and confidence intervals, we run the same analysis using an adherence model with Box–Cox-transformed data that resulted in a model with much better residual distribution as shown in **Supplementary** **Figure 3A and 3B** (and random effects distributions shown in **Supplementary** **Figure 2E and 2F**). The forest plot of the linear regression coefficient is shown in **Supplementary** **Figure 4** and the corresponding results in **Supplementary** **Table 3** show that both log-transformed and Box–Cox-transformed models find almost exactly the same set of statistically significant coefficients.

#### 4. EQ-5D anxiety effect

To study the effect of EQ-5D Anxiety/Depression score, we fitted the same adherence and Box–Cox-transformed regression models by subsetting the datasets to only the patients who completed the EQ-5D (either 3-level or 5-level version) at any point in the trial (removing 53.9% of patients and 46.9% patient-days, see **Table 1**) and adding the categorical variable EQ-5D anxiety. **Supplementary Figure 5** **and** **Supplementary** Figure 6 (along with **Supplementary Table 7** **and Supplementary Table 8**) show coefficients of adherence and Box-Cox-transformed dose consistency models respectively. Although the numerical values of all coefficients are different from original models their relative sizes, signs, and statistical significance was largely unchanged except for the highest level of NYHA and CCS Angina grade classifications in the adherence and dose consistency models, respectively, which are no longer statistically significant (at α=0.1%). This could be due to significantly lower sample size that were included in this model.

#### 5. Comparison of patients with higher versus lower initial dose consistency

To study whether early dose consistency has a bearing on later adherence or dose consistency patterns, we divided the adherence and dose consistency datasets into two equally sized groups of patients according to their average dose consistency between day 7 (to minimize any transient effects at the start of the trial) and day 35. Only patients who had at least 10 days of data during that period were included in this analysis. The same adherence and Box–Cox-transformed dose consistency models were fitted as before.

Results from these analyses are plotted in **Supplementary Figure 7** and **Supplementary Figure 8** for the dose consistency and adherence models, respectively, with detailed results in **Supplementary Table 9, Supplementary Table 10, Supplementary Table 11**, and **Supplementary Table 12**. In the analysis of patients who established an early dose consistency, those who had a higher dose consistency early in the treatment period not only continued to exhibit a higher dose consistency until the end of the study but also had significantly higher adherence until a year into the study, after which time the adherence rates started to converge for the two groups of patients (**Supplementary Figure 7** and **Supplementary Figure 8**). Second, patients with a higher initial dose consistency had a greater effect size of high CCS Angina grade than patients with lower initial dose consistency, for whom CCS Angina grade was not statistically significant.

### Supplementary Figure 1. Distribution of all adherence times in the dose consistency dataset

Distribution of adherence times in the dose consistency dataset for all patients is shown in A. The distribution of dose consistency (the absolute value of adherence time differences on consecutive days) truncated at 12 h for clarity is shown in B. The full distribution but on logarithmic scale is shown in C. The distribution of dose consistency transformed with logarithmic transformation and Box–Cox transformation (λ= 0.150081) are shown in panels D and E, respectively.

### Supplementary Figure 2. Random effects distribution

Random effects distribution for patient (left) and site (right) for adherence logistic mixed-effects model (A and B, respectively), log-transformed dose consistency linear mixed-effects model (C and D, respectively), and Box–Cox-transformed dose consistency linear mixed-effects model (E and F, respectively).

### Supplementary Figure 3. Residual distribution diagnostic plots for linear regression models

Quantile–quantile plots for Box–Cox-transformed (A) and log-transformed (C) dose consistency (the absolute value of adherence time differences on consecutive days) linear mixed-effects regression models from Supplementary Table 5 and Supplementary Table 4, respectively. The red lines shows the expected normal distribution. Panels B and C show the corresponding fitted versus residual plots with a linear fit line (red) for the Box–Cox-transformed and log-transformed dose consistency models, respectively, showing absence of heteroscedasticity.

### Supplementary Figure 4. Box–Cox-transformed dose consistency model forest plot

Coefficients of the linear mixed effects regression model fit on the Box–Cox-transformed dose consistency (the absolute value of adherence time differences on consecutive days). Variables with multiple values are dichotomized and reference values are indicated by (ref). Error bars represent 99·95% confidence intervals (see corresponding results in Supplementary Table 5).

AE=adverse event; BMI=body mass index; CCS=Canadian Cardiovascular Society; eGFR=estimated glomerular filtration rate; LVEF=left ventricular ejection fraction; NYHA=New York Heart Association; STEMI=ST-elevation myocardial infarction.

### Supplementary Figure 5. Adherence model forest plot for the anxiety subset

Odds ratios of the logistic mixed effects regression model fit on the subset of adherence dataset including only patients who provided responses to EQ-5D Anxiety/Depression question. Variables with multiple values are dichotomized and reference values are indicated by (ref). Error bars represent 99·95% confidence intervals (see corresponding results in Supplementary Table 7).

AE=adverse event; BMI=body mass index; CCS=Canadian Cardiovascular Society; eGFR=estimated glomerular filtration rate; LVEF=left ventricular ejection fraction; NYHA=New York Heart Association; STEMI=ST-elevation myocardial infarction.

### Supplementary Figure 6. Box–Cox-transformed dose consistency model forest plot for the anxiety subset

Coefficients of the linear mixed effects regression model fit on the subset of the Box–Cox-transformed dose consistency (the absolute value of adherence time differences on consecutive days) including only patients who provided responses to EQ-5D Anxiety/Depression question. Variables with multiple values are dichotomized and reference values are indicated by (ref). Error bars represent 99·95% confidence intervals (see corresponding results in Supplementary Table 8).

AE=adverse event; BMI=body mass index; CCS=Canadian Cardiovascular Society; eGFR=estimated glomerular filtration rate; LVEF=left ventricular ejection fraction; NYHA=New York Heart Association; STEMI=ST-elevation myocardial infarction.

### Supplementary Figure 7. Adherence model forest plots for patients with low and high initial dose consistency

Odds ratios of the logistic mixed effects regression model fit on two subsets of the adherence dataset – low (blue) and high (red) initial dose consistency. The dataset was divided according to the average dose consistency between days 7 and 35. Only patients who had at least 10 days of data during that period were included in this analysis. Variables with multiple values are dichotomized and reference values are indicated by (ref). Error bars represent 99·95% confidence intervals (see corresponding results in Supplementary Tables 9 and 10).

AE=adverse event; BMI=body mass index; CCS=Canadian Cardiovascular Society; eGFR=estimated glomerular filtration rate; LVEF=left ventricular ejection fraction; NYHA=New York Heart Association; STEMI=ST-elevation myocardial infarction.

### Supplementary Figure 8. Box–Cox-transformed dose consistency model forest plots for patients with low and high initial dose consistency

Coefficients of the linear mixed effects regression model fit on two subsets of the Box–Cox-transformed dose consistency (the absolute value of adherence time differences on consecutive days). The dataset was divided according to the average dose consistency between days 7 and 35. Only patients who had at least 10 days of data during that period were included in this analysis. Variables with multiple values are dichotomized and reference values are indicated by (ref). Error bars represent 99·95% confidence intervals (see corresponding results in Supplementary Tables 11 and 12).

AE=adverse event; BMI=body mass index; CCS=Canadian Cardiovascular Society; eGFR=estimated glomerular filtration rate; LVEF=left ventricular ejection fraction; NYHA=New York Heart Association; STEMI=ST-elevation myocardial infarction.

### Supplementary Table 1. Independent variables used in adherence and dose consistency models

| Group | Variable | Number of levels | Levels | Reference level | Comment | Adherence model | Dose consistency model |
| --- | --- | --- | --- | --- | --- | --- | --- |
| Patient characteristics | **Country** | 2 | Sweden, UK | UK |  | Yes | Yes |
|  | **Sex** | 2 | Male, Female | Male |  | Yes | Yes |
|  | **Age** | 6 | <40, 40–50, 50–60, 60–70, 70–80, >80 | 60–70 | Left-closed intervals only, e.g. 60-70 refers to [60–70), i.e. it includes 60-year-olds but not 70-year-olds | Yes | Yes |
|  | **Race** | 3 | White, Asian, Other | White |  | Yes | Yes |
|  | **Arm** | 2 | Placebo, Dapagliflozin 10 mg | Placebo |  | Yes | Yes |
|  | **Smoking status** | 3 | Never, Former, Current | Never |  | Yes | Yes |
|  | **Hypertension history** | 2 | Yes, No | No |  | Yes | Yes |
| Health status | **Systolic pressure** | 2 | <130, ≥130 | <130 |  | Yes | Yes |
|  | **Diastolic pressure** | 2 | <80, ≥80 | <80 |  | Yes | Yes |
|  | **BMI** | 2 | <30, ≥30 | <30 |  | Yes | Yes |
|  | **LVEF** | 3 | ≥50, 30–49, <30 | ≥50 | Left ventricular ejection fraction | Yes | Yes |
|  | **eGFR** | 2 | ≥60, <60 | ≥60 | Estimated glomerular filtration rate | Yes | Yes |
|  | **STEMI** | 2 | STEMI, non STEMI | non STEMI | ST-elevation myocardial infarction status of the index event | Yes | Yes |
|  | **Revascularization** | 2 | No, Yes | No |  | Yes | Yes |
|  | **# medications** | 2 | >12, ≤12 | ≤12 | Number of concomitant medications during the trial | Yes | Yes |
|  | **NYHA** | 4 | No disease, No symptoms, Mild symptoms, Marked/Severe | No disease | New York Heart Association Functional Classification. The top two levels (Marked and Severe symptoms, levels III and IV) were merged into one level because of very few responses | Yes | Yes |
|  | **CCS Angina grade** | 4 | Asymptomatic, Strenuous exertion, Moderate exertion, Mild exertion/At rest | Asymptomatic | Canadian Cardiovascular Society Angina Grade. The top two levels (angina symptoms at rest and upon mild exertion) were merged into one level because of very few responses | Yes | Yes |
| Calendar features | **Days from start** | 13 | ≤30, 31–60, 61–90, 91–120, 121–150, 151–200, 201–250, 251–300, 301–350, 351–400, 401–450, 451–500, >500 | ≤30 | Number of days from the beginning of treatment. Right-closed intervals (i.e. including the upper boundary) | Yes | Yes |
|  | **Month** | 12 | January, February, March, April, May, June, July, August, September, October, November, December | January |  | Yes | Yes |
|  | **Days of the week** | 7 | Monday, Tuesday, Wednesday, Thursday, Friday, Saturday, Sunday | Wednesday |  | Yes | Yes |
|  | **Holiday** | 2 | Not holiday, Holiday | Not holiday | Country-specific public holidays extracted with workalendar 16.4.0 library | Yes | Yes |
|  | **Study close-out** | 2 | No, Yes | No | Dates after second amendment was approved; after 27 February 2023. | Yes | Yes |
| AEs | **Hospitalized or discontinued** | 5 | No AE, Neither, Discontinued only, Hospitalized only, Both | No AE | AEs that required either hospitalization, study drug discontinuation, both or neither | Yes | Yes |
|  | **7 days after AE** | 5 | No AE, Not hospitalized or discontinued, Not hospitalized but discontinued, Hospitalized, not discontinued, Hospitalized and discontinued | No AE | A week following the AE requiring either hospitalization, study drug discontinuation, both or neither | Yes | Yes |
| Behavioural features | **Mobile application use** | 2 | Yes, No | No | Day when mobile app was used for EQ-5D completion | Yes | Yes |
|  | **7 days after mobile application use** | 2 | Yes, No | No | Seven days following mobile app use for EQ-5D completion | Yes | Yes |
|  | **Off-schedule use in last 7 days** | 2 | Yes, No | No | Days with recent off-schedule smart bottle use | Yes | Yes |
|  | **Average time in last 7 days** | 6 | 0–4, 4–8, 8–12, 12–16, 16–20, 20–24 | 8–12 | Average time over previous 7 days when smart bottle was opened (ignoring days when no data are available). See Supplementary Table 13 for example calculations. | Yes | Yes |
|  | **Average time variance in last 7 days** | 5 | <15 min, 15–30 min, 30–60 min, 1–2 h, >2 h | >2 h | Mean absolute error of adherence time over previous 7 days (ignoring days when no data are available). See Supplementary Table 13 for example calculations. | Yes | No |
|  | **Days adherent in last 7 days** | 8 | 0, 1, 2, 3, 4, 5, 6, 7 | 7 | Average adherence over previous 7 days (ignoring days when no data are available) | Yes | Yes |
| Disruptions | **7 days after data gap** | 2 | Yes, No | No | 7 days following a missing data | Yes | Yes |
|  | **7 days after a holiday** | 2 | Yes, No | No | 7 days following a public holiday | Yes | Yes |
|  | **After a missed dose** | 15 | No recent non-adherence, 1 day after ≤3 missed, 2 days after ≤3 missed, 3 days after ≤3 missed, 4 days after ≤3 missed, 5 days after ≤3 missed, 6 days after ≤3 missed, 7 days after ≤3 missed, 1 day after >3 missed, 2 days after >3 missed, 3 days after >3 missed, 4 days after >3 missed, 5 days after >3 missed, 6 days after >3 missed, 7 days after >3 missed | No recent non-adherence | 7 days following a short (≤3) or longer (>3) streak of missed doses | No | Yes |
| Other | **Lost >5% weight** | 2 | No, Yes | No | Patients who lost >5% of body weight between first and last visit | Yes | Yes |
|  | **COVID-19 restrictions** | 2 | Yes, No | No | Days when a country where the site is located experienced any COVID-19 related restriction | Yes | Yes |
|  | **EQ-5D anxiety** | 6 | No, Moderate (3L), Extreme (3L), Slight (5L), Moderate (5L), Severe/Extreme (5L) | No | Response to anxiety/depression question in EQ-5D using 3- or 5-level scales (3L or 5L respectively) | No | No |

AE=adverse event; BMI=body mass index; CCS=Canadian Cardiovascular Society; eGFR=estimated glomerular filtration rate; LVEF=left ventricular ejection fraction; ML=machine learning; NYHA=New York Heart Association; STEMI=ST-elevation myocardial infarction.

### Supplementary Table 2. Summary of adherence and dose consistency datasets

|  |  |  | Adherence | | | Dose consistency | | |
| --- | --- | --- | --- | --- | --- | --- | --- | --- |
|  |  |  | Patients, n | Observations, n | Adherence rate (%) | Patients, n | Observations, n | Time difference [sec] (median, IQR) |
| Group | **Feature** | **Value** |  |  |  |  |  |  |
| Adherence | **Adherence** | **Yes** | 2429 (100·0%) | 685 160 (93·4%) | 100·0% | 2429 (100·0%) | 682731 (100·0%) | 2080 [709, 4997] |
|  |  | **No*** | 2202 (90·7%) | 48330 (6·6%) | 0·0% | 0 (0·0%) | 0 (0·0%) |  |
| Patient characteristics | **Country** | **Sweden** | 534 (22·0%) | 188420 (25·7%) | 94·8% | 534 (22·0%) | 178078 (26·1%) | 1803 [615, 4409] |
|  |  | **UK*** | 1895 (78·0%) | 545070 (74·3%) | 92·9% | 1895 (78·0%) | 504653 (73·9%) | 2189 [748, 5205] |
|  | **Sex** | **Male*** | 1945 (80·1%) | 589831 (80·4%) | 93·3% | 1945 (80·1%) | 548226 (80·3%) | 2117 [716, 5133] |
|  |  | **Female** | 484 (19·9%) | 143659 (19·6%) | 94·0% | 484 (19·9%) | 134505 (19·7%) | 1942 [681, 4486] |
|  | **Age** | **<40** | 51 (2·1%) | 12629 (1·7%) | 89·0% | 51 (2·1%) | 11186 (1·6%) | 2879 [905, 6950] |
|  |  | **40–50** | 228 (9·4%) | 63465 (8·7%) | 91·6% | 228 (9·4%) | 57877 (8·5%) | 2751 [910, 6500] |
|  |  | **50–60** | 688 (28·3%) | 195265 (26·6%) | 92·4% | 688 (28·3%) | 179659 (26·3%) | 2535 [826, 6146] |
|  |  | **60–70*** | 813 (33·5%) | 257202 (35·1%) | 94·1% | 813 (33·5%) | 241157 (35·3%) | 2103 [732, 4854] |
|  |  | **70–80** | 547 (22·5%) | 174248 (23·8%) | 94·4% | 547 (22·5%) | 163909 (24·0%) | 1621 [576, 3822] |
|  |  | **>80** | 102 (4·2%) | 30681 (4·2%) | 94·7% | 102 (4·2%) | 28943 (4·2%) | 1413 [518·5, 3355] |
|  | **Race** | **White*** | 2314 (95·3%) | 703485 (95·9%) | 93·6% | 2314 (95·3%) | 655987 (96·1%) | 2062 [701, 4955] |
|  |  | **Asian** | 77 (3·2%) | 20712 (2·8%) | 89·1% | 77 (3·2%) | 18382 (2·7%) | 2699 [965, 6324·5] |
|  |  | **Other** | 36 (1·5%) | 8656 (1·2%) | 90·1% | 36 (1·5%) | 7762 (1·1%) | 2347 [833, 5523·5] |
|  |  | **Missing** | 2 (0·1%) | 637 (0·1%) | 94·5% | 2 (0·1%) | 600 (0·1%) | 2542 [797·5, 6239·25] |
|  | **Arm** | **Placebo*** | 1228 (50·6%) | 369338 (50·4%) | 93·4% | 1228 (50·6%) | 343551 (50·3%) | 2090 [730, 4962] |
|  |  | **Dapagliflozin 10 mg** | 1201 (49·4%) | 364152 (49·6%) | 93·5% | 1201 (49·4%) | 339180 (49·7%) | 2068 [687, 5035] |
|  | **Smoking status** | **Never*** | 1043 (42·9%) | 325824 (44·4%) | 94·2% | 1043 (42·9%) | 305831 (44·8%) | 1930 [664, 4572] |
|  |  | **Former** | 690 (28·4%) | 212052 (28·9%) | 93·9% | 690 (28·4%) | 198520 (29·1%) | 1958 [678, 4690] |
|  |  | **Current** | 695 (28·6%) | 195544 (26·7%) | 91·6% | 695 (28·6%) | 178331 (26·1%) | 2553 [849, 6167·5] |
|  |  | **Missing** | 1 (0·0%) | 70 (0·0%) | 71·4% | 1 (0·0%) | 49 (0·0%) | 6022 [2246, 13858] |
|  | **Hypertension history** | **Yes** | 918 (37·8%) | 274892 (37·5%) | 93·9% | 918 (37·8%) | 257228 (37·7%) | 2017 [694, 4852] |
|  |  | **No*** | 1494 (61·5%) | 454548 (62·0%) | 93·1% | 1494 (61·5%) | 421786 (61·8%) | 2117 [717, 5084] |
|  |  | **Missing** | 17 (0·7%) | 4050 (0·6%) | 92·2% | 17 (0·7%) | 3717 (0·5%) | 2359 [880, 5590] |
| Health status | **Systolic pressure** | **<130*** | 1882 (77·5%) | 564298 (76·9%) | 93·4% | 1882 (77·5%) | 524940 (76·9%) | 2085 [708, 5014] |
|  |  | **≥130** | 547 (22·5%) | 169192 (23·1%) | 93·6% | 547 (22·5%) | 157791 (23·1%) | 2060 [713, 4940] |
|  | **Diastolic pressure** | **<80*** | 1834 (75·5%) | 550190 (75·0%) | 93·5% | 1834 (75·5%) | 512475 (75·1%) | 2040 [702, 4874] |
|  |  | **≥80** | 595 (24·5%) | 183300 (25·0%) | 93·2% | 595 (24·5%) | 170256 (24·9%) | 2206 [733, 5382·25] |
|  | **BMI** | **<30*** | 1641 (67·6%) | 501 448 (68·4%) | 93·6% | 1641 (67·6%) | 46 7751 (68·5%) | 1987 [676, 4799·5] |
|  |  | **≥30** | 772 (31·8%) | 228 181 (31·1%) | 92·9% | 772 (31·8%) | 211 257 (30·9%) | 2294 [787, 5414] |
|  |  | **Missing** | 16 (0·7%) | 3861 (0·5%) | 96·8% | 16 (0·7%) | 3723 (0·5%) | 2391 [797, 5907] |
|  | **LVEF** | **≥50*** | 505 (20·8%) | 123 664 (16·9%) | 92·5% | 505 (20·8%) | 113 901 (16·7%) | 2171 [734, 5225] |
|  |  | **30–49** | 1596 (65·7%) | 514 445 (70·1%) | 93·7% | 1596 (65·7%) | 480 306 (70·4%) | 2036 [690, 4899] |
|  |  | **<30** | 164 (6·8%) | 43 874 (6·0%) | 93·6% | 164 (6·8%) | 40 903 (6·0%) | 2057 [756, 4786] |
|  |  | **Missing** | 164 (6·8%) | 51 507 (7·0%) | 92·8% | 164 (6·8%) | 47 621 (7·0%) | 2365 [805, 5650] |
|  | **eGFR** | **≥60*** | 2188 (90·1%) | 658 622 (89·8%) | 93·4% | 2188 (90·1%) | 612 731 (89·7%) | 2114 [716, 5085] |
|  |  | **<60** | 240 (9·9%) | 74 657 (10·2%) | 93·9% | 240 (9·9%) | 69 834 (10·2%) | 1815 [651, 4286] |
|  |  | **Missing** | 1 (0·0%) | 211 (0·0%) | 79·1% | 1 (0·0%) | 166 (0·0%) | 2318 [1225·5, 4964·75] |
|  | **STEMI** | **STEMI** | 1780 (73·3%) | 539 315 (73·5%) | 93·4% | 1780 (73·3%) | 502 161 (73·6%) | 2087 [715, 5002] |
|  |  | **non STEMI*** | 643 (26·5%) | 191 981 (26·2%) | 93·3% | 643 (26·5%) | 178 512 (26·1%) | 2056 [685·75, 4989] |
|  |  | **Missing** | 6 (0·2%) | 2194 (0·3%) | 94·1% | 6 (0·2%) | 2058 (0·3%) | 2341 [1021, 4545·75] |
|  | **Revascularization** | **No*** | 2427 (99·9%) | 694 907 (94·7%) | 93·4% | 2424 (99·8%) | 646 677 (94·7%) | 2084 [708, 5002] |
|  |  | **Yes** | 170 (7·0%) | 38 583 (5·3%) | 93·5% | 169 (7·0%) | 36 054 (5·3%) | 2001 [729, 4897] |
|  | **# medications** | **>12** | 1185 (48·8%) | 364 730 (49·7%) | 93·2% | 1185 (48·8%) | 338 762 (49·6%) | 2131 [737, 5073] |
|  |  | **≤12*** | 1244 (51·2%) | 368 760 (50·3%) | 93·6% | 1244 (51·2%) | 343 969 (50·4%) | 2030 [681, 4923] |
|  | **NYHA** | **No disease*** | 1399 (57·6%) | 330 707 (45·1%) | 93·7% | 1372 (56·5%) | 308 651 (45·2%) | 2011 [678, 4870] |
|  |  | **No symptoms** | 1157 (47·6%) | 250 910 (34·2%) | 93·2% | 1136 (46·8%) | 233 131 (34·1%) | 2156 [736, 5123] |
|  |  | **Mild symptoms** | 608 (25·0%) | 130 107 (17·7%) | 93·6% | 599 (24·7%) | 121 357 (17·8%) | 2045 [701, 4952] |
|  |  | **Marked/Severe** | 125 (5·1%) | 19 701 (2·7%) | 91·7% | 122 (5·0%) | 17 985 (2·6%) | 2607 [976, 5880] |
|  |  | **Missing** | 31 (1·3%) | 2065 (0·3%) | 79·3% | 31 (1·3%) | 1607 (0·2%) | 1729 [702·5, 3928] |
|  | **CCS Angina grade** | **Asymptomatic*** | 2012 (82·8%) | 553 943 (75·5%) | 93·7% | 2004 (82·5%) | 517 444 (75·8%) | 2031 [689, 4888] |
|  |  | **Strenuous exertion** | 670 (27·6%) | 138 181 (18·8%) | 92·9% | 653 (26·9%) | 127 845 (18·7%) | 2198 [751, 5256] |
|  |  | **Moderate exertion** | 147 (6·1%) | 30 849 (4·2%) | 90·9% | 144 (5·9%) | 27 928 (4·1%) | 2539 [878, 5944·5] |
|  |  | **Mild exertion/At rest** | 50 (2·1%) | 8475 (1·2%) | 94·0% | 50 (2·1%) | 7933 (1·2%) | 2095 [756, 4596] |
|  |  | **Missing** | 33 (1·4%) | 2042 (0·3%) | 79·0% | 33 (1·4%) | 1581 (0·2%) | 2314 [878, 5588] |
| Calendar features | **Days from start** | **≤30*** | 2405 (99·0%) | 72 498 (9·9%) | 94·7% | 2398 (98·7%) | 66 243 (9·7%) | 1966 [711, 4580] |
|  |  | **31–60** | 2350 (96·7%) | 68 868 (9·4%) | 94·0% | 2340 (96·3%) | 64 710 (9·5%) | 2019 [704, 4709] |
|  |  | **61–90** | 2292 (94·4%) | 66 801 (9·1%) | 93·2% | 2285 (94·1%) | 62 252 (9·1%) | 2060·5 [703, 4887] |
|  |  | **91–120** | 2189 (90·1%) | 61 156 (8·3%) | 92·8% | 2172 (89·4%) | 56 770 (8·3%) | 2090 [700, 5027] |
|  |  | **121–150** | 1946 (80·1%) | 55 026 (7·5%) | 92·5% | 1931 (79·5%) | 50 901 (7·5%) | 2127 [711, 5088] |
|  |  | **151–200** | 1790 (73·7%) | 81 487 (11·1%) | 92·6% | 1777 (73·2%) | 75 440 (11·0%) | 2112 [717, 5113·25] |
|  |  | **201–250** | 1541 (63·4%) | 69 156 (9·4%) | 93·1% | 1530 (63·0%) | 64 397 (9·4%) | 2096 [696, 5068] |
|  |  | **251–300** | 1333 (54·9%) | 61 160 (8·3%) | 93·1% | 1323 (54·5%) | 56 955 (8·3%) | 2052 [684, 5121] |
|  |  | **301–350** | 1189 (49·0%) | 51 798 (7·1%) | 93·2% | 1180 (48·6%) | 48 257 (7·1%) | 2126 [718, 5122] |
|  |  | **351–400** | 953 (39·2%) | 40 532 (5·5%) | 94·3% | 947 (39·0%) | 38 203 (5·6%) | 2125 [727, 5263·5] |
|  |  | **401–450** | 749 (30·8%) | 33 741 (4·6%) | 94·5% | 745 (30·7%) | 31 878 (4·7%) | 2154 [739, 5216·75] |
|  |  | **451–500** | 611 (25·2%) | 27 217 (3·7%) | 93·6% | 609 (25·1%) | 25 478 (3·7%) | 2210 [735, 5366] |
|  |  | **>500** | 477 (19·6%) | 44 050 (6·0%) | 93·6% | 475 (19·6%) | 41 247 (6·0%) | 2045 [698, 4963·5] |
|  | **Month** | **January*** | 2049 (84·4%) | 77 354 (10·5%) | 93·8% | 2036 (83·8%) | 72 361 (10·6%) | 2166 [733, 5177] |
|  |  | **February** | 2152 (88·6%) | 75 200 (10·3%) | 93·6% | 2131 (87·7%) | 70 121 (10·3%) | 2054 [686, 5037] |
|  |  | **March** | 2175 (89·5%) | 84 977 (11·6%) | 92·8% | 2158 (88·8%) | 78 699 (11·5%) | 2036 [695, 4895] |
|  |  | **April** | 2065 (85·0%) | 65 950 (9·0%) | 92·1% | 2047 (84·3%) | 60 560 (8·9%) | 2139 [748, 5070·25] |
|  |  | **May** | 1730 (71·2%) | 47 429 (6·5%) | 92·7% | 1702 (70·1%) | 43 762 (6·4%) | 2111 [737·25, 5096] |
|  |  | **June** | 1369 (56·4%) | 39 728 (5·4%) | 94·0% | 1353 (55·7%) | 37 157 (5·4%) | 2095 [712, 4951] |
|  |  | **July** | 1392 (57·3%) | 44 791 (6·1%) | 93·8% | 1385 (57·0%) | 41 867 (6·1%) | 2063 [716, 4928] |
|  |  | **August** | 1496 (61·6%) | 48 453 (6·6%) | 93·7% | 1469 (60·5%) | 45 242 (6·6%) | 2028 [688, 4908·75] |
|  |  | **September** | 1626 (66·9%) | 52 044 (7·1%) | 93·9% | 1602 (66·0%) | 48 611 (7·1%) | 1978 [665, 4778·5] |
|  |  | **October** | 1725 (71·0%) | 60 224 (8·2%) | 94·0% | 1712 (70·5%) | 56 396 (8·3%) | 2074·5 [697, 4968·25] |
|  |  | **November** | 1844 (75·9%) | 64 546 (8·8%) | 93·6% | 1829 (75·3%) | 60 133 (8·8%) | 2000 [680, 4859] |
|  |  | **December** | 1951 (80·3%) | 72 794 (9·9%) | 93·5% | 1940 (79·9%) | 67 822 (9·9%) | 2179·5 [757, 5194·75] |
|  | **Days of the week** | **Monday** | 2428 (100·0%) | 104 782 (14·3%) | 93·6% | 2427 (99·9%) | 97 874 (14·3%) | 2822 [1005, 6436] |
|  |  | **Tuesday** | 2429 (100·0%) | 104 894 (14·3%) | 93·8% | 2424 (99·8%) | 97 897 (14·3%) | 1831 [602, 4544] |
|  |  | **Wednesday*** | 2429 (100·0%) | 104 815 (14·3%) | 93·7% | 2427 (99·9%) | 97 656 (14·3%) | 1715 [566, 4272] |
|  |  | **Thursday** | 2429 (100·0%) | 104 811 (14·3%) | 93·7% | 2427 (99·9%) | 97 682 (14·3%) | 1754 [576, 4308] |
|  |  | **Friday** | 2429 (100·0%) | 104 885 (14·3%) | 93·4% | 2428 (100·0%) | 97 473 (14·3%) | 1833 [612, 4489] |
|  |  | **Saturday** | 2429 (100·0%) | 104 662 (14·3%) | 92·8% | 2426 (99·9%) | 96 995 (14·2%) | 2551 [921, 5872·5] |
|  |  | **Sunday** | 2429 (100·0%) | 104 641 (14·3%) | 92·9% | 2424 (99·8%) | 97 154 (14·2%) | 2260 [847, 5101] |
|  | **Holiday** | **Not holiday*** | 2429 (100·0%) | 704 789 (96·1%) | 93·4% | 2429 (100·0%) | 656 155 (96·1%) | 2066 [702, 4982] |
|  |  | **Holiday** | 2343 (96·5%) | 28 701 (3·9%) | 92·7% | 2316 (95·3%) | 26 576 (3·9%) | 2398 [887, 5402·25] |
|  | **Study Closeout** | **No*** | 2380 (98·0%) | 626 032 (85·3%) | 93·9% | 2375 (97·8%) | 585 291 (85·7%) | 2062 [703, 4953] |
|  |  | **Yes** | 2057 (84·7%) | 107 458 (14·7%) | 90·7% | 2045 (84·2%) | 97 440 (14·3%) | 2191 [748, 5274·25] |
| AEs | **Hospitalized or discontinued** | **No AE*** | 2409 (99·2%) | 693 448 (94·5%) | 93·5% | 2402 (98·9%) | 645 937 (94·6%) | 2089 [712, 5014] |
|  |  | **Neither** | 286 (11·8%) | 28 491 (3·9%) | 95·1% | 277 (11·4%) | 27072 (4·0%) | 1888·5 [636, 4594·25] |
|  |  | **Discontinued only** | 47 (1·9%) | 1619 (0·2%) | 77·9% | 40 (1·6%) | 1260 (0·2%) | 2039·5 [690·25, 4667·5] |
|  |  | **Hospitalized only** | 309 (12·7%) | 7122 (1·0%) | 87·5% | 288 (11·9%) | 6230 (0·9%) | 2050·5 [699, 5024·75] |
|  |  | **Both** | 122 (5·0%) | 2810 (0·4%) | 79·5% | 101 (4·2%) | 2232 (0·3%) | 2049·5 [664·25, 5055·5] |
|  | **7 days after AE** | **No AE*** | 2429 (100·0%) | 729 169 (99·4%) | 93·4% | 2429 (100·0%) | 678 868 (99·4%) | 2079 [708, 4996] |
|  |  | **Not hospitalized or discontinued** | 177 (7·3%) | 1436 (0·2%) | 93·2% | 176 (7·2%) | 1339 (0·2%) | 2311 [773, 5097] |
|  |  | **Not hospitalized but discontinued** | 15 (0·6%) | 108 (0·0%) | 63·9% | 11 (0·5%) | 69 (0·0%) | 3437 [1382, 7477] |
|  |  | **Hospitalized, not discontinued** | 265 (10·9%) | 2246 (0·3%) | 91·5% | 263 (10·8%) | 2053 (0·3%) | 2017 [741, 4884] |
|  |  | **Hospitalized and discontinued** | 73 (3·0%) | 531 (0·1%) | 75·7% | 67 (2·8%) | 402 (0·1%) | 2796 [1020·5, 5845] |
| Behavioural features | **Mobile application use** | **Yes** | 684 (28·2%) | 3375 (0·5%) | 96·2% | 647 (26·6%) | 3130 (0·5%) | 1957·5 [610·75, 4883·75] |
|  |  | **No*** | 2429 (100·0%) | 730 115 (99·5%) | 93·4% | 2429 (100·0%) | 679 601 (99·5%) | 2080 [709, 4997] |
|  | **7 days after mobile application use** | **Yes** | 685 (28·2%) | 23 471 (3·2%) | 95·7% | 682 (28·1%) | 22 447 (3·3%) | 1901 [630·5, 4580] |
|  |  | **No*** | 2429 (100·0%) | 710 019 (96·8%) | 93·3% | 2429 (100·0%) | 660 284 (96·7%) | 2086 [711, 5011] |
|  | **Off-schedule use in last 7 days** | **Yes** | 2258 (93·0%) | 69 019 (9·4%) | 88·0% | 2253 (92·8%) | 60 719 (8·9%) | 2685 [926, 6426] |
|  |  | **No*** | 2429 (100·0%) | 664 471 (90·6%) | 94·0% | 2429 (100·0%) | 622 012 (91·1%) | 2030 [692, 4870] |
|  | **Average time in last 7 days** | **0–4** | 181 (7·5%) | 4436 (0·6%) | 91·5% | 181 (7·5%) | 4049 (0·6%) | 2242 [884, 5122] |
|  |  | **4–8** | 1631 (67·1%) | 211 528 (28·8%) | 95·6% | 1631 (67·1%) | 201 812 (29·6%) | 1571 [483, 4158] |
|  |  | **8–12*** | 2160 (88·9%) | 397 029 (54·1%) | 94·1% | 2160 (88·9%) | 372 077 (54·5%) | 2185 [807, 4944] |
|  |  | **12–16** | 967 (39·8%) | 51 293 (7·0%) | 85·6% | 967 (39·8%) | 43 713 (6·4%) | 4312 [1454, 10514] |
|  |  | **16–20** | 607 (25·0%) | 40 434 (5·5%) | 88·1% | 607 (25·0%) | 35 472 (5·2%) | 2477·5 [772, 6330·75] |
|  |  | **20–24** | 280 (11·5%) | 28 770 (3·9%) | 89·3% | 280 (11·5%) | 25 608 (3·8%) | 1893·5 [691, 4211·25] |
|  | **Average time variance in last 7 days** | **<15 min** | 1376 (56·6%) | 63 391 (8·6%) | 97·2% | 1376 (56·6%) | 61 520 (9·0%) | 451 [137, 1130] |
|  |  | **15–30 min** | 2115 (87·1%) | 119 872 (16·3%) | 96·4% | 2115 (87·1%) | 115 416 (16·9%) | 1270 [515, 2597] |
|  |  | **30–60 min** | 2346 (96·6%) | 231 844 (31·6%) | 95·0% | 2346 (96·6%) | 219 842 (32·2%) | 2040 [786, 4231] |
|  |  | **1–2 h** | 2323 (95·6%) | 198 391 (27·0%) | 93·5% | 2323 (95·6%) | 184 435 (27·0%) | 3201 [1153, 6904] |
|  |  | **>2 h*** | 1941 (79·9%) | 119 992 (16·4%) | 85·1% | 1941 (79·9%) | 101 518 (14·9%) | 5163 [1822, 12 262·75] |
| Disruptions | **7 days after data gap** | **Yes** | 971 (40·0%) | 12 250 (1·7%) | 89·9% | 964 (39·7%) | 10 668 (1·6%) | 2973 [963, 7955·25] |
|  |  | **No*** | 2428 (100·0%) | 721 240 (98·3%) | 93·5% | 2428 (100·0%) | 672 063 (98·4%) | 2069 [705, 4963] |
|  | **7 days after a holiday** | **Yes** | 2343 (96·5%) | 94025 (12·8%) | 93·3% | 2330 (95·9%) | 87 715 (12·8%) | 2153 [746, 5118] |
|  |  | **No*** | 2429 (100·0%) | 639 465 (87·2%) | 93·4% | 2429 (100·0%) | 595 016 (87·2%) | 2069 [704, 4979] |
|  | **After a missed dose** | **No recent non-adherence*** | 2429 (100·0%) | 612 656 (83·5%) | 92·1% | 2423 (99·8%) | 561 939 (82·3%) | 1922 [653, 4557] |
|  |  | **1 day after ≤3 missed** | 2123 (87·4%) | 23225 (3·2%) | 100·0% | 2115 (87·1%) | 23 188 (3·4%) | 4372 [1505, 11 262·25] |
|  |  | **2 days after ≤3 missed** | 2100 (86·5%) | 19 673 (2·7%) | 100·0% | 2100 (86·5%) | 19 673 (2·9%) | 3236 [1115, 7955] |
|  |  | **3 days after ≤3 missed** | 2093 (86·2%) | 17 303 (2·4%) | 100·0% | 2093 (86·2%) | 17 303 (2·5%) | 2861 [992·5, 7007·5] |
|  |  | **4 days after ≤3 missed** | 2082 (85·7%) | 15 539 (2·1%) | 100·0% | 2082 (85·7%) | 15 539 (2·3%) | 2737 [943, 6482] |
|  |  | **5 days after ≤3 missed** | 2073 (85·3%) | 14 167 (1·9%) | 100·0% | 2073 (85·3%) | 14 166 (2·1%) | 2631·5 [924·25, 6381·5] |
|  |  | **6 days after ≤3 missed** | 2066 (85·1%) | 12 960 (1·8%) | 100·0% | 2066 (85·1%) | 12 960 (1·9%) | 2735 [951, 6346·25] |
|  |  | **7 days after ≤3 missed** | 2052 (84·5%) | 11 763 (1·6%) | 100·0% | 2052 (84·5%) | 11 763 (1·7%) | 2706 [962·5, 6432·5] |
|  |  | **1 day after >3 missed** | 722 (29·7%) | 1958 (0·3%) | 100·0% | 720 (29·6%) | 1954 (0·3%) | 6552 [2183·5, 16 909·75] |
|  |  | **2 days after >3 missed** | 630 (25·9%) | 1057 (0·1%) | 100·0% | 630 (25·9%) | 1057 (0·2%) | 3647 [1321, 11 004] |
|  |  | **3 days after >3 missed** | 566 (23·3%) | 810 (0·1%) | 100·0% | 566 (23·3%) | 810 (0·1%) | 2760·5 [857·5, 6953·5] |
|  |  | **4 days after >3 missed** | 514 (21·2%) | 691 (0·1%) | 100·0% | 514 (21·2%) | 691 (0·1%) | 3007 [921·5, 6788] |
|  |  | **5 days after >3 missed** | 472 (19·4%) | 613 (0·1%) | 100·0% | 472 (19·4%) | 613 (0·1%) | 2647 [986, 5981] |
|  |  | **6 days after >3 missed** | 432 (17·8%) | 554 (0·1%) | 100·0% | 432 (17·8%) | 554 (0·1%) | 2727·5 [979·25, 5498·75] |
|  |  | **7 days after >3 missed** | 416 (17·1%) | 521 (0·1%) | 100·0% | 416 (17·1%) | 521 (0·1%) | 2572 [836, 5642] |
| Other | **Lost >5% weight** | **No*** | 1849 (76·1%) | 556 313 (75·8%) | 93·3% | 1849 (76·1%) | 517 456 (75·8%) | 2163 [747, 5154] |
|  |  | **Yes** | 580 (23·9%) | 177 177 (24·2%) | 93·6% | 580 (23·9%) | 165 275 (24·2%) | 1832 [601, 4515] |
|  | **COVID-19 restrictions** | **Yes** | 2159 (88·9%) | 517 467 (70·5%) | 94·0% | 2158 (88·8%) | 484 294 (70·9%) | 2043 [700, 4901] |
|  |  | **No*** | 2125 (87·5%) | 216 023 (29·5%) | 92·0% | 2120 (87·3%) | 198 437 (29·1%) | 2173 [732, 5239] |
|  | **EQ-5D Anxiety** | **No*** | 851 (35·0%) | 251 594 (34·3%) | 94·2% | 851 (35·0%) | 236 420 (34·6%) | 1903 [646, 4668] |
|  |  | **Moderate (3L)** | 155 (6·4%) | 40 156 (5·5%) | 95·5% | 155 (6·4%) | 38 228 (5·6%) | 1842 [620, 4391·5] |
|  |  | **Extreme (3L)** | 12 (0·5%) | 3681 (0·5%) | 95·0% | 12 (0·5%) | 3489 (0·5%) | 2017 [780, 4901] |
|  |  | **Slight (5L)** | 356 (14·7%) | 67 569 (9·2%) | 94·3% | 356 (14·7%) | 63 498 (9·3%) | 2315·5 [789, 5451] |
|  |  | **Moderate (5L)** | 125 (5·1%) | 17 626 (2·4%) | 92·5% | 125 (5·1%) | 16 246 (2·4%) | 2234 [754, 5425·25] |
|  |  | **Severe/Extreme (5L)** | 28 (1·2%) | 5292 (0·7%) | 94·4% | 28 (1·2%) | 4979 (0·7%) | 3165 [1282·5, 6585] |
|  |  | **Missing** | 1309 (53·9%) | 347 572 (47·4%) | 92·4% | 1309 (53·9%) | 319 871 (46·9%) | 2184 [749, 5180] |

Adherence dataset and dose consistency datasets summarized by number of patients, number of observations, adherence rate, as well as median and IQR of dose consistency for all levels (including the missing values) of all independent variables included in the adherence and dose consistency models. Asterisks indicate the reference level used in adherence and dose consistency models.

AE=adverse event; BMI=body mass index; CCS=Canadian Cardiovascular Society; eGFR=estimated glomerular filtration rate; IQR=interquartile range; LVEF=left ventricular ejection fraction; NYHA=New York Heart Association; STEMI=ST-elevation myocardial infarction.

### Supplementary Table 3. Results of adherence model

| Group | Feature | Value | Odds ratio | Estimate | 0·05% CI | 99·95% CI | SE | z value | Pr(>\|z\|) | No. patients | No. observations |
| --- | --- | --- | --- | --- | --- | --- | --- | --- | --- | --- | --- |
| Patient characteristics | **Country** | Sweden | 1·19 | 0·1735 | –0·2005 | 0·5476 | 0·1137 | 1·5 | 0·13 | 534 (22·0%) | 188 420 (25·7%) |
|  | **Sex** | Female | 1·172 | 0·1588 | –0·1076 | 0·4251 | 0·081 | 2 | 0·05 | 484 (19·9%) | 143 659 (19·6%) |
|  | **Age** | <40 | 0·471 | –0·7529 | –1·487 | –0·0189 | 0·2231 | –3·4 | 0·00074 | 51 (2·1%) | 12 629 (1·7%) |
|  |  | 40–50 | 0·653 | –0·4261 | –0·8142 | –0·038 | 0·1179 | –3·6 | 0·0003 | 228 (9·4%) | 63 465 (8·7%) |
|  |  | 50–60 | 0·811 | –0·2099 | –0·4748 | 0·055 | 0·0805 | –2·6 | 0·0091 | 688 (28·3%) | 195 265 (26·6%) |
|  |  | 60–70 (ref) |  |  |  |  |  |  |  | 813 (33·5%) | 257 202 (35·1%) |
|  |  | 70–80 | 0·933 | –0·0696 | –0·3646 | 0·2254 | 0·0897 | –0·8 | 0·44 | 547 (22·5%) | 174 248 (23·8%) |
|  |  | >80 | 0·903 | –0·1017 | –0·6676 | 0·4643 | 0·172 | –0·6 | 0·55 | 102 (4·2%) | 30681 (4·2%) |
|  | **Race** | White (ref) |  |  |  |  |  |  |  | 2316 (95·3%) | 704 122 (96·0%) |
|  |  | Asian | 0·837 | –0·1785 | –0·7837 | 0·4267 | 0·1839 | –1 | 0·33 | 77 (3·2%) | 20712 (2·8%) |
|  |  | Other | 0·617 | –0·4827 | –1·4295 | 0·4641 | 0·2877 | –1·7 | 0·093 | 36 (1·5%) | 8656 (1·2%) |
|  | **Arm** | Dapagliflozin 10 mg | 1·007 | 0·0065 | –0·2006 | 0·2136 | 0·0629 | 0·1 | 0·92 | 1201 (49·4%) | 364 152 (49·6%) |
|  | **Smoking status** | Never (ref) |  |  |  |  |  |  |  | 1044 (43·0%) | 325 894 (44·4%) |
|  |  | Former | 0·972 | –0·0285 | –0·2806 | 0·2235 | 0·0766 | –0·4 | 0·71 | 690 (28·4%) | 212 052 (28·9%) |
|  |  | Current | 0·668 | –0·4041 | –0·6646 | –0·1436 | 0·0792 | –5·1 | 3·3e-07 | 695 (28·6%) | 195 544 (26·7%) |
|  | **Hypertension history** | Yes | 0·941 | –0·0612 | –0·286 | 0·1636 | 0·0683 | –0·9 | 0·37 | 918 (37·8%) | 274 892 (37·5%) |
| Health status | **Systolic pressure** | ≥130 | 0·978 | –0·0219 | –0·3013 | 0·2574 | 0·0849 | –0·3 | 0·8 | 547 (22·5%) | 169 192 (23·1%) |
|  | **Diastolic pressure** | ≥80 | 0·89 | –0·1161 | –0·3864 | 0·1541 | 0·0821 | –1·4 | 0·16 | 595 (24·5%) | 183 300 (25·0%) |
|  | **BMI** | ≥30 | 1·012 | 0·0116 | –0·2163 | 0·2396 | 0·0693 | 0·2 | 0·87 | 772 (31·8%) | 228 181 (31·1%) |
|  | **LVEF** | ≥50 (ref) |  |  |  |  |  |  |  | 505 (20·8%) | 123 664 (16·9%) |
|  |  | 30–49 | 1·058 | 0·0568 | –0·2084 | 0·322 | 0·0806 | 0·7 | 0·48 | 1760 (72·5%) | 565 952 (77·2%) |
|  |  | <30 | 1·116 | 0·1098 | –0·3695 | 0·589 | 0·1456 | 0·8 | 0·45 | 164 (6·8%) | 43 874 (6·0%) |
|  | **eGFR** | <60 | 0·968 | –0·0326 | –0·3968 | 0·3316 | 0·1107 | –0·3 | 0·77 | 240 (9·9%) | 74 657 (10·2%) |
|  | **STEMI** | STEMI | 1·141 | 0·1319 | –0·1167 | 0·3805 | 0·0756 | 1·7 | 0·081 | 1786 (73·5%) | 541 509 (73·8%) |
|  | **Revascularization** | Yes | 1·057 | 0·0551 | –0·1198 | 0·23 | 0·0532 | 1 | 0·3 | 170 (7·0%) | 38 583 (5·3%) |
|  | **# medications** | >12 | 0·9 | –0·1059 | –0·327 | 0·1152 | 0·0672 | –1·6 | 0·11 | 1185 (48·8%) | 364 730 (49·7%) |
|  | **NYHA** | No disease (ref) |  |  |  |  |  |  |  | 1430 (58·9%) | 332 772 (45·4%) |
|  |  | No symptoms | 1·047 | 0·0462 | –0·0559 | 0·1483 | 0·031 | 1·5 | 0·14 | 1157 (47·6%) | 250 910 (34·2%) |
|  |  | Mild symptoms | 1·11 | 0·1044 | –0·0194 | 0·2282 | 0·0376 | 2·8 | 0·0055 | 608 (25·0%) | 130 107 (17·7%) |
|  |  | Marked/Severe | 1·483 | 0·3939 | 0·1669 | 0·6209 | 0·069 | 5·7 | 1·1e-08 | 125 (5·1%) | 19 701 (2·7%) |
|  | **CCS Angina grade** | Asymptomatic (ref) |  |  |  |  |  |  |  | 2045 (84·2%) | 555 985 (75·8%) |
|  |  | Strenuous exertion | 0·999 | –0·0005 | –0·1101 | 0·109 | 0·0333 | 0 | 0·99 | 670 (27·6%) | 138 181 (18·8%) |
|  |  | Moderate exertion | 0·938 | –0·0639 | –0·2576 | 0·1298 | 0·0589 | –1·1 | 0·28 | 147 (6·1%) | 30 849 (4·2%) |
|  |  | Mild exertion/At rest | 1·081 | 0·078 | –0·3846 | 0·5407 | 0·1406 | 0·6 | 0·58 | 50 (2·1%) | 8475 (1·2%) |
| Calendar features | **Days from start** | ≤30 (ref) |  |  |  |  |  |  |  | 2405 (99·0%) | 72 498 (9·9%) |
|  |  | 31–60 | 0·885 | –0·1218 | –0·2099 | –0·0337 | 0·0268 | –4·5 | 5·4e-06 | 2350 (96·7%) | 68 868 (9·4%) |
|  |  | 61–90 | 0·718 | –0·3313 | –0·4194 | –0·2431 | 0·0268 | –12·4 | 4·3e-35 | 2292 (94·4%) | 66 801 (9·1%) |
|  |  | 91–120 | 0·6 | –0·5113 | –0·6015 | –0·4211 | 0·0274 | –18·7 | 1·3e-77 | 2189 (90·1%) | 61 156 (8·3%) |
|  |  | 121–150 | 0·521 | –0·6511 | –0·7438 | –0·5583 | 0·0282 | –23·1 | 4·5e-118 | 1946 (80·1%) | 55 026 (7·5%) |
|  |  | 151–200 | 0·512 | –0·6704 | –0·7587 | –0·582 | 0·0268 | –25 | 1·2e-137 | 1790 (73·7%) | 81 487 (11·1%) |
|  |  | 201–250 | 0·544 | –0·6087 | –0·7031 | –0·5143 | 0·0287 | –21·2 | 5·9e-100 | 1541 (63·4%) | 69 156 (9·4%) |
|  |  | 251–300 | 0·523 | –0·6474 | –0·7471 | –0·5476 | 0·0303 | –21·4 | 3·6e-101 | 1333 (54·9%) | 61 160 (8·3%) |
|  |  | 301–350 | 0·526 | –0·6422 | –0·749 | –0·5355 | 0·0324 | –19·8 | 3·4e-87 | 1189 (49·0%) | 51 798 (7·1%) |
|  |  | 351–400 | 0·625 | –0·4707 | –0·5894 | –0·352 | 0·0361 | –13 | 6·7e-39 | 953 (39·2%) | 40 532 (5·5%) |
|  |  | 401–450 | 0·594 | –0·5209 | –0·65 | –0·3918 | 0·0392 | –13·3 | 3·2e-40 | 749 (30·8%) | 33 741 (4·6%) |
|  |  | 451–500 | 0·504 | –0·6858 | –0·8235 | –0·5481 | 0·0419 | –16·4 | 2·5e-60 | 611 (25·2%) | 27 217 (3·7%) |
|  |  | >500 | 0·483 | –0·7284 | –0·8668 | –0·59 | 0·0421 | –17·3 | 3·4e-67 | 477 (19·6%) | 44 050 (6·0%) |
|  | **Month** | January (ref) |  |  |  |  |  |  |  | 2049 (84·4%) | 77 354 (10·5%) |
|  |  | February | 0·949 | –0·0523 | –0·1322 | 0·0277 | 0·0243 | –2·1 | 0·032 | 2152 (88·6%) | 75 200 (10·3%) |
|  |  | March | 1·073 | 0·0704 | –0·0463 | 0·1871 | 0·0355 | 2 | 0·047 | 2175 (89·5%) | 84 977 (11·6%) |
|  |  | April | 0·888 | –0·1189 | –0·2332 | –0·0046 | 0·0347 | –3·4 | 0·00062 | 2065 (85·0%) | 65 950 (9·0%) |
|  |  | May | 0·797 | –0·2267 | –0·3432 | –0·1101 | 0·0354 | –6·4 | 1·5e-10 | 1730 (71·2%) | 47 429 (6·5%) |
|  |  | June | 0·849 | –0·1638 | –0·2862 | –0·0413 | 0·0372 | –4·4 | 1·1e-05 | 1369 (56·4%) | 39 728 (5·4%) |
|  |  | July | 0·808 | –0·2132 | –0·3342 | –0·0922 | 0·0368 | –5·8 | 6·7e-09 | 1392 (57·3%) | 44 791 (6·1%) |
|  |  | August | 0·811 | –0·2099 | –0·3301 | –0·0898 | 0·0365 | –5·7 | 9e-09 | 1496 (61·6%) | 48 453 (6·6%) |
|  |  | September | 0·817 | –0·2023 | –0·3218 | –0·0827 | 0·0363 | –5·6 | 2·6e-08 | 1626 (66·9%) | 52 044 (7·1%) |
|  |  | October | 0·858 | –0·1536 | –0·2731 | –0·0341 | 0·0363 | –4·2 | 2·3e-05 | 1725 (71·0%) | 60 224 (8·2%) |
|  |  | November | 0·8 | –0·2236 | –0·342 | –0·1053 | 0·036 | –6·2 | 5e-10 | 1844 (75·9%) | 64 546 (8·8%) |
|  |  | December | 0·809 | –0·2118 | –0·3289 | –0·0947 | 0·0356 | –6 | 2·6e-09 | 1951 (80·3%) | 72 794 (9·9%) |
|  | **Days of the week** | Monday | 1 | –0·0001 | –0·0667 | 0·0665 | 0·0202 | 0 | 1·0 | 2428 (100·0%) | 104 782 (14·3%) |
|  |  | Tuesday | 1·035 | 0·0348 | –0·0313 | 0·101 | 0·0201 | 1·7 | 0·083 | 2429 (100·0%) | 104 894 (14·3%) |
|  |  | Wednesday (ref) |  |  |  |  |  |  |  | 2429 (100·0%) | 104 815 (14·3%) |
|  |  | Thursday | 0·996 | –0·0037 | –0·0696 | 0·0621 | 0·02 | –0·2 | 0·85 | 2429 (100·0%) | 104 811 (14·3%) |
|  |  | Friday | 0·951 | –0·0498 | –0·1153 | 0·0157 | 0·0199 | –2·5 | 0·012 | 2429 (100·0%) | 104 885 (14·3%) |
|  |  | Saturday | 0·843 | –0·1708 | –0·2351 | –0·1065 | 0·0195 | –8·7 | 2·2e-18 | 2429 (100·0%) | 104 662 (14·3%) |
|  |  | Sunday | 0·856 | –0·1554 | –0·2201 | –0·0907 | 0·0197 | –7·9 | 2·7e-15 | 2429 (100·0%) | 104 641 (14·3%) |
|  | **Holiday** | Holiday | 0·906 | –0·0991 | –0·1895 | –0·0087 | 0·0275 | –3·6 | 0·00031 | 2343 (96·5%) | 28 701 (3·9%) |
|  | **Study Closeout** | Yes | 0·655 | –0·4232 | –0·537 | –0·3094 | 0·0346 | –12·2 | 2e-34 | 2057 (84·7%) | 107 458 (14·7%) |
| AEs | **Hospitalized or discontinued** | No AE (ref) |  |  |  |  |  |  |  | 2409 (99·2%) | 693 448 (94·5%) |
|  |  | Neither | 0·645 | –0·4389 | –0·6268 | –0·251 | 0·0571 | –7·7 | 1·5e-14 | 286 (11·8%) | 28 491 (3·9%) |
|  |  | Discontinued only | 0·075 | –2·5931 | –3·0156 | –2·1705 | 0·1284 | –20·2 | 1·1e-90 | 47 (1·9%) | 1619 (0·2%) |
|  |  | Hospitalized only | 0·246 | –1·4021 | –1·5962 | –1·2081 | 0·059 | –23·8 | 5·4e-125 | 309 (12·7%) | 7122 (1·0%) |
|  |  | Both | 0·089 | –2·4205 | –2·6892 | –2·1518 | 0·0817 | –29·6 | 4·2e-193 | 122 (5·0%) | 2810 (0·4%) |
|  | **7 days after AE** | No AE (ref) |  |  |  |  |  |  |  | 2429 (100·0%) | 729 169 (99·4%) |
|  |  | Not hospitalized or discontinued | 0·874 | –0·1348 | –0·5262 | 0·2566 | 0·1189 | –1·1 | 0·26 | 177 (7·3%) | 1436 (0·2%) |
|  |  | Not hospitalized but discontinued | 0·09 | –2·4054 | –3·1936 | –1·6172 | 0·2395 | –10 | 1e-23 | 15 (0·6%) | 108 (0·0%) |
|  |  | Hospitalized, not discontinued | 0·726 | –0·3199 | –0·6084 | –0·0315 | 0·0877 | –3·6 | 0·00026 | 265 (10·9%) | 2246 (0·3%) |
|  |  | Hospitalized and discontinued | 0·223 | –1·5027 | –1·8961 | –1·1093 | 0·1196 | –12·6 | 3·1e-36 | 73 (3·0%) | 531 (0·1%) |
| Behavioural features | **Mobile application use** | Yes | 1·347 | 0·2978 | –0·0259 | 0·6215 | 0·0984 | 3 | 0·0025 | 684 (28·2%) | 3375 (0·5%) |
|  | **7 days after mobile application use** | Yes | 1·205 | 0·1861 | 0·0621 | 0·3102 | 0·0377 | 4·9 | 7·9e-07 | 685 (28·2%) | 23 471 (3·2%) |
|  | **Off-schedule use in last 7 days** | Yes | 0·536 | –0·6232 | –0·6739 | –0·5724 | 0·0154 | –40·4 | 0·0 | 2258 (93·0%) | 69 019 (9·4%) |
|  | **Average time in last 7 days** | 0–4 | 0·808 | –0·2136 | –0·4476 | 0·0205 | 0·0711 | –3 | 0·0027 | 181 (7·5%) | 4436 (0·6%) |
|  |  | 4–8 | 1·076 | 0·0732 | 0·011 | 0·1354 | 0·0189 | 3·9 | 0·00011 | 1631 (67·1%) | 211 528 (28·8%) |
|  |  | 8–12 (ref) |  |  |  |  |  |  |  | 2160 (88·9%) | 397 029 (54·1%) |
|  |  | 12–16 | 0·768 | –0·2637 | –0·34 | –0·1875 | 0·0232 | –11·4 | 5·2e-30 | 967 (39·8%) | 51 293 (7·0%) |
|  |  | 16–20 | 0·737 | –0·3045 | –0·4098 | –0·1991 | 0·032 | –9·5 | 1·9e-21 | 607 (25·0%) | 40 434 (5·5%) |
|  |  | 20–24 | 0·593 | –0·5224 | –0·659 | –0·3858 | 0·0415 | –12·6 | 2·6e-36 | 280 (11·5%) | 28 770 (3·9%) |
|  | **Average time variance in last 7 days** | <15 min | 1·993 | 0·6895 | 0·5759 | 0·8031 | 0·0345 | 20 | 1·1e-88 | 1376 (56·6%) | 63 391 (8·6%) |
|  |  | 15–30 min | 1·75 | 0·5597 | 0·4808 | 0·6385 | 0·024 | 23·4 | 1·1e-120 | 2115 (87·1%) | 119 872 (16·3%) |
|  |  | 30–60 min | 1·408 | 0·3425 | 0·2837 | 0·4013 | 0·0179 | 19·2 | 6·5e-82 | 2346 (96·6%) | 231 844 (31·6%) |
|  |  | 1–2 h | 1·318 | 0·2759 | 0·2236 | 0·3281 | 0·0159 | 17·4 | 1·2e-67 | 2323 (95·6%) | 198 391 (27·0%) |
|  |  | >2 h (ref) |  |  |  |  |  |  |  | 1941 (79·9%) | 119 992 (16·4%) |
| Disruptions | **7 days after data gap** | Yes | 1·023 | 0·0228 | –0·0988 | 0·1445 | 0·037 | 0·6 | 0·54 | 971 (40·0%) | 12 250 (1·7%) |
|  | **7 days after a holiday** | Yes | 1·011 | 0·0112 | –0·0453 | 0·0676 | 0·0172 | 0·7 | 0·52 | 2343 (96·5%) | 94 025 (12·8%) |
| Other | **Lost >5% weight** | Yes | 0·936 | –0·0665 | –0·3118 | 0·1787 | 0·0745 | –0·9 | 0·37 | 580 (23·9%) | 177 177 (24·2%) |
|  | **Covid-19 restrictions** | Yes | 1·177 | 0·163 | 0·0478 | 0·2782 | 0·035 | 4·7 | 3·2e-06 | 2159 (88·9%) | 517 467 (70·5%) |
|  | **Intercept** |  | 53·241 | 3·9748 | 3·5426 | 4·4071 | 0·1314 | 30·3 | 3·9e-201 | 2429 (100%) | 733 490 (100%) |

Results of the logistic mixed effects regression model fit on the Adherence Dataset. Variables with multiple values are dichotomized and reference values are indicated by (ref). Error bars represent 99·95% CIs.

AE=adverse event; BMI=body mass index; CCS=Canadian Cardiovascular Society; CI=confidence interval; eGFR=estimated glomerular filtration rate; LVEF=left ventricular ejection fraction; NYHA=New York Heart Association; SE=standard error; STEMI=ST-elevation myocardial infarction.

### Supplementary Table 4. Summary statistics for patient and site random effects for adherence, log-transformed and Box–Cox-transformed dose consistency models

| Model | Group effect (SD) | 0·05% CI | 99·95% CI | Estimate |
| --- | --- | --- | --- | --- |
| Adherence | **Patient** | 1·350816189 | 1·516806659 | 1·431407346 |
|  | **Site** | 0·190601292 | 0·538476667 | 0·320365961 |
|  | **Residual** |  |  |  |
| Dose consistency model (log) | **Patient** | 0·642547758 | 0·709920865 | 0·675394744 |
|  | **Site** | 0·036788667 | 0·222512624 | 0·0904762 |
|  | **Residual** |  |  | 1·379479612 |
| Dose consistency model (Box–Cox) | **Patient** | 0·3978586 | 0·439665625 | 0·418240062 |
|  | **Site** | 0·024249683 | 0·134899642 | 0·057195049 |
|  | **Residual** |  |  | 0·871457878 |

SD, standard deviation.

### Supplementary Table 5. Results of log-transformed dose consistency

| Group | Feature | Value | Dose consistency model | Estimate | 0·05% CI | 99·95% CI | SE | t value | Pr(>\|t\|) | No. patients | No. observations |
| --- | --- | --- | --- | --- | --- | --- | --- | --- | --- | --- | --- |
| Patient characteristics | Country | Sweden | 0·973 | –0·0278 | –0·1683 | 0·1127 | 0·0427 | –0·7 | 0·51 | 534 (22·0%) | 178 078 (26·1%) |
|  | Sex | Female | 0·929 | –0·0739 | –0·1926 | 0·0447 | 0·0361 | –2·1 | 0·04 | 484 (19·9%) | 134 505 (19·7%) |
|  | Age | <40 | 1·183 | 0·168 | –0·1719 | 0·508 | 0·1033 | 1·6 | 0·1 | 51 (2·1%) | 11 186 (1·6%) |
|  |  | 40–50 | 1·228 | 0·2054 | 0·0284 | 0·3823 | 0·0538 | 3·8 | 0·00013 | 228 (9·4%) | 57 877 (8·5%) |
|  |  | 50–60 | 1·17 | 0·1571 | 0·0374 | 0·2768 | 0·0364 | 4·3 | 1·6e-05 | 688 (28·3%) | 179 659 (26·3%) |
|  |  | 60–70 (ref) |  |  |  |  |  |  |  | 813 (33·5%) | 241 157 (35·3%) |
|  |  | 70–80 | 0·839 | –0·1754 | –0·3075 | –0·0434 | 0·0401 | –4·4 | 1·2e-05 | 547 (22·5%) | 163 909 (24·0%) |
|  |  | >80 | 0·762 | –0·2715 | –0·523 | –0·02 | 0·0764 | –3·6 | 0·00038 | 102 (4·2%) | 28 943 (4·2%) |
|  | Race | White (ref) |  |  |  |  |  |  |  | 2316 (95·3%) | 656 587 (96·2%) |
|  |  | Asian | 1·181 | 0·1662 | –0·1092 | 0·4415 | 0·0837 | 2 | 0·047 | 77 (3·2%) | 18 382 (2·7%) |
|  |  | Other | 1·203 | 0·185 | –0·2208 | 0·5907 | 0·1233 | 1·5 | 0·13 | 36 (1·5%) | 7762 (1·1%) |
|  | Arm | Dapagliflozin 10 mg | 0·974 | –0·0267 | –0·1201 | 0·0667 | 0·0284 | –0·9 | 0·35 | 1201 (49·4%) | 339 180 (49·7%) |
|  | Smoking status | Never (ref) |  |  |  |  |  |  |  | 1044 (43·0%) | 305 880 (44·8%) |
|  |  | Former | 1·044 | 0·0432 | –0·0697 | 0·156 | 0·0343 | 1·3 | 0·21 | 690 (28·4%) | 198 520 (29·1%) |
|  |  | Current | 1·164 | 0·1521 | 0·0343 | 0·2698 | 0·0358 | 4·3 | 2·1e-05 | 695 (28·6%) | 178 331 (26·1%) |
|  | Hypertension history | Yes | 1·024 | 0·0237 | –0·0776 | 0·1249 | 0·0308 | 0·8 | 0·44 | 918 (37·8%) | 257 228 (37·7%) |
| Health status | Systolic pressure | ≥130 | 1·056 | 0·0541 | –0·0719 | 0·18 | 0·0383 | 1·4 | 0·16 | 547 (22·5%) | 157 791 (23·1%) |
|  | Diastolic pressure | ≥80 | 0·994 | –0·0058 | –0·1274 | 0·1158 | 0·037 | –0·2 | 0·87 | 595 (24·5%) | 170 256 (24·9%) |
|  | BMI | ≥30 | 1·083 | 0·08 | –0·0228 | 0·1827 | 0·0312 | 2·6 | 0·01 | 772 (31·8%) | 211 257 (30·9%) |
|  | LVEF | ≥50 (ref) |  |  |  |  |  |  |  | 505 (20·8%) | 113 901 (16·7%) |
|  |  | 30–49 | 0·977 | –0·0238 | –0·142 | 0·0944 | 0·0359 | –0·7 | 0·51 | 1760 (72·5%) | 527 927 (77·3%) |
|  |  | <30 | 0·962 | –0·0391 | –0·2521 | 0·1739 | 0·0647 | –0·6 | 0·55 | 164 (6·8%) | 40 903 (6·0%) |
|  | eGFR | <60 | 0·997 | –0·003 | –0·1664 | 0·1605 | 0·0497 | –0·1 | 0·95 | 240 (9·9%) | 69 834 (10·2%) |
|  | STEMI | STEMI | 0·995 | –0·0052 | –0·1151 | 0·1047 | 0·0334 | –0·2 | 0·88 | 1786 (73·5%) | 504 219 (73·9%) |
|  | Revascularization | Yes | 0·979 | –0·0208 | –0·0768 | 0·0353 | 0·017 | –1·2 | 0·22 | 169 (7·0%) | 36 054 (5·3%) |
|  | # medications | >12 | 1·031 | 0·0304 | –0·0684 | 0·1291 | 0·03 | 1 | 0·31 | 1185 (48·8%) | 338 762 (49·6%) |
|  | NYHA | No disease (ref) |  |  |  |  |  |  |  | 1403 (57·8%) | 310 258 (45·4%) |
|  |  | No symptoms | 1·021 | 0·021 | –0·0103 | 0·0523 | 0·0095 | 2·2 | 0·027 | 1136 (46·8%) | 233 131 (34·1%) |
|  |  | Mild symptoms | 1·025 | 0·0249 | –0·0139 | 0·0637 | 0·0118 | 2·1 | 0·035 | 599 (24·7%) | 121 357 (17·8%) |
|  |  | Marked/Severe | 1·036 | 0·0354 | –0·0464 | 0·1172 | 0·0248 | 1·4 | 0·15 | 122 (5·0%) | 17 985 (2·6%) |
|  | CCS Angina grade | Asymptomatic (ref) |  |  |  |  |  |  |  | 2037 (83·9%) | 519 025 (76·0%) |
|  |  | Strenuous exertion | 1·006 | 0·0062 | –0·0302 | 0·0427 | 0·0111 | 0·6 | 0·57 | 653 (26·9%) | 127 845 (18·7%) |
|  |  | Moderate exertion | 0·944 | –0·0581 | –0·1292 | 0·013 | 0·0216 | –2·7 | 0·0071 | 144 (5·9%) | 27 928 (4·1%) |
|  |  | Mild exertion/At rest | 0·834 | –0·181 | –0·3038 | –0·0583 | 0·0373 | –4·9 | 1·2e-06 | 50 (2·1%) | 7933 (1·2%) |
| Calendar features | Days from start | ≤30 (ref) |  |  |  |  |  |  |  | 2398 (98·7%) | 66 243 (9·7%) |
|  |  | 31–60 | 0·995 | –0·0048 | –0·0303 | 0·0207 | 0·0077 | –0·6 | 0·53 | 2340 (96·3%) | 64 710 (9·5%) |
|  |  | 61–90 | 1·033 | 0·0325 | 0·0064 | 0·0585 | 0·0079 | 4·1 | 4·1e-05 | 2285 (94·1%) | 62 252 (9·1%) |
|  |  | 91–120 | 1·078 | 0·0747 | 0·0478 | 0·1016 | 0·0082 | 9·1 | 6·9e-20 | 2172 (89·4%) | 56 770 (8·3%) |
|  |  | 121–150 | 1·093 | 0·0888 | 0·0608 | 0·1169 | 0·0085 | 10·4 | 2·1e-25 | 1931 (79·5%) | 50 901 (7·5%) |
|  |  | 151–200 | 1·123 | 0·1163 | 0·0901 | 0·1424 | 0·0079 | 14·6 | 1·4e-48 | 1777 (73·2%) | 75 440 (11·0%) |
|  |  | 201–250 | 1·115 | 0·1092 | 0·0815 | 0·137 | 0·0084 | 13 | 1·8e-38 | 1530 (63·0%) | 64 397 (9·4%) |
|  |  | 251–300 | 1·128 | 0·1205 | 0·0911 | 0·1499 | 0·0089 | 13·5 | 1·8e-41 | 1323 (54·5%) | 56 955 (8·3%) |
|  |  | 301–350 | 1·155 | 0·1437 | 0·1121 | 0·1753 | 0·0096 | 15 | 9·9e-51 | 1180 (48·6%) | 48 257 (7·1%) |
|  |  | 351–400 | 1·164 | 0·1518 | 0·1174 | 0·1862 | 0·0104 | 14·5 | 7·5e-48 | 947 (39·0%) | 38 203 (5·6%) |
|  |  | 401–450 | 1·174 | 0·1607 | 0·1234 | 0·1981 | 0·0113 | 14·2 | 1·4e-45 | 745 (30·7%) | 31 878 (4·7%) |
|  |  | 451–500 | 1·191 | 0·1745 | 0·1334 | 0·2155 | 0·0125 | 14 | 2·1e-44 | 609 (25·1%) | 25 478 (3·7%) |
|  |  | >500 | 1·186 | 0·171 | 0·1305 | 0·2114 | 0·0123 | 13·9 | 5·2e-44 | 475 (19·6%) | 41 247 (6·0%) |
|  | Month | January (ref) |  |  |  |  |  |  |  | 2036 (83·8%) | 72 361 (10·6%) |
|  |  | February | 0·988 | –0·012 | –0·0368 | 0·0128 | 0·0075 | –1·6 | 0·11 | 2131 (87·7%) | 70 121 (10·3%) |
|  |  | March | 0·975 | –0·0253 | –0·06 | 0·0094 | 0·0105 | –2·4 | 0·016 | 2158 (88·8%) | 78 699 (11·5%) |
|  |  | April | 0·992 | –0·0084 | –0·0426 | 0·0259 | 0·0104 | –0·8 | 0·42 | 2047 (84·3%) | 60 560 (8·9%) |
|  |  | May | 1·038 | 0·0375 | 0·0025 | 0·0725 | 0·0106 | 3·5 | 0·00042 | 1702 (70·1%) | 43 762 (6·4%) |
|  |  | June | 1·046 | 0·0446 | 0·0085 | 0·0808 | 0·011 | 4·1 | 4·8e-05 | 1353 (55·7%) | 37 157 (5·4%) |
|  |  | July | 1·03 | 0·0298 | –0·006 | 0·0656 | 0·0109 | 2·7 | 0·0062 | 1385 (57·0%) | 41 867 (6·1%) |
|  |  | August | 1·007 | 0·0073 | –0·0282 | 0·0428 | 0·0108 | 0·7 | 0·5 | 1469 (60·5%) | 45 242 (6·6%) |
|  |  | September | 0·987 | –0·0128 | –0·0479 | 0·0223 | 0·0107 | –1·2 | 0·23 | 1602 (66·0%) | 48 611 (7·1%) |
|  |  | October | 0·994 | –0·0056 | –0·0405 | 0·0294 | 0·0106 | –0·5 | 0·6 | 1712 (70·5%) | 56 396 (8·3%) |
|  |  | November | 0·98 | –0·0205 | –0·0552 | 0·0142 | 0·0105 | –1·9 | 0·052 | 1829 (75·3%) | 60 133 (8·8%) |
|  |  | December | 1·041 | 0·0405 | 0·0062 | 0·0748 | 0·0104 | 3·9 | 0·0001 | 1940 (79·9%) | 67 822 (9·9%) |
|  | Days of the week | Monday | 1·578 | 0·456 | 0·4352 | 0·4768 | 0·0063 | 72·2 | 0·0 | 2427 (99·9%) | 97 874 (14·3%) |
|  |  | Tuesday | 1·06 | 0·0581 | 0·0375 | 0·0786 | 0·0062 | 9·3 | 1·5e-20 | 2424 (99·8%) | 97 897 (14·3%) |
|  |  | Wednesday (ref) |  |  |  |  |  |  |  | 2427 (99·9%) | 97 656 (14·3%) |
|  |  | Thursday | 1·016 | 0·0163 | –0·0043 | 0·0368 | 0·0062 | 2·6 | 0·0092 | 2427 (99·9%) | 97 682 (14·3%) |
|  |  | Friday | 1·067 | 0·0644 | 0·0438 | 0·0851 | 0·0063 | 10·3 | 7·9e-25 | 2428 (100·0%) | 97 473 (14·3%) |
|  |  | Saturday | 1·469 | 0·3843 | 0·3636 | 0·4049 | 0·0063 | 61·3 | 0·0 | 2426 (99·9%) | 96 995 (14·2%) |
|  |  | Sunday | 1·321 | 0·2787 | 0·258 | 0·2994 | 0·0063 | 44·3 | 0·0 | 2424 (99·8%) | 97 154 (14·2%) |
|  | Holiday | Holiday | 1·021 | 0·0204 | –0·0095 | 0·0503 | 0·0091 | 2·2 | 0·025 | 2316 (95·3%) | 26 576 (3·9%) |
|  | Study Closeout | Yes | 1·028 | 0·0276 | –0·0073 | 0·0626 | 0·0106 | 2·6 | 0·0093 | 2045 (84·2%) | 97 440 (14·3%) |
| AEs | Hospitalized or discontinued | No AE (ref) |  |  |  |  |  |  |  | 2402 (98·9%) | 645 937 (94·6%) |
|  |  | Neither | 1·152 | 0·1411 | 0·085 | 0·1972 | 0·0171 | 8·3 | 1·3e-16 | 277 (11·4%) | 27 072 (4·0%) |
|  |  | Discontinued only | 1·165 | 0·1524 | –0·0313 | 0·3361 | 0·0558 | 2·7 | 0·0063 | 40 (1·6%) | 1260 (0·2%) |
|  |  | Hospitalized only | 1·232 | 0·2083 | 0·1299 | 0·2866 | 0·0238 | 8·7 | 2·2e-18 | 288 (11·9%) | 6230 (0·9%) |
|  |  | Both | 1·2 | 0·1825 | 0·0437 | 0·3213 | 0·0422 | 4·3 | 1·5e-05 | 101 (4·2%) | 2232 (0·3%) |
|  | 7 days after AE | No AE (ref) |  |  |  |  |  |  |  | 2429 (100·0%) | 678 868 (99·4%) |
|  |  | Not hospitalized or discontinued | 0·981 | –0·0187 | –0·1453 | 0·1079 | 0·0385 | –0·5 | 0·63 | 176 (7·2%) | 1339 (0·2%) |
|  |  | Not hospitalized but discontinued | 1·692 | 0·5258 | –0·0307 | 1·0824 | 0·1691 | 3·1 | 0·0019 | 11 (0·5%) | 69 (0·0%) |
|  |  | Hospitalized, not discontinued | 1·036 | 0·0353 | –0·0676 | 0·1381 | 0·0313 | 1·1 | 0·26 | 263 (10·8%) | 2053 (0·3%) |
|  |  | Hospitalized and discontinued | 1·151 | 0·1402 | –0·0906 | 0·371 | 0·0701 | 2 | 0·046 | 67 (2·8%) | 402 (0·1%) |
| Behavioural features | Mobile application use | Yes | 1·092 | 0·0877 | 0·0054 | 0·1701 | 0·025 | 3·5 | 0·00045 | 647 (26·6%) | 3130 (0·5%) |
|  | 7 days after mobile application use | Yes | 1·014 | 0·014 | –0·0193 | 0·0473 | 0·0101 | 1·4 | 0·17 | 682 (28·1%) | 22 447 (3·3%) |
|  | Off-schedule use in last 7 days | Yes | 1·098 | 0·0932 | 0·0728 | 0·1137 | 0·0062 | 15 | 6·4e-51 | 2253 (92·8%) | 60 719 (8·9%) |
|  | Average time in last 7 days | 0–4 | 1·079 | 0·0763 | –0·0093 | 0·1618 | 0·026 | 2·9 | 0·0034 | 181 (7·5%) | 4049 (0·6%) |
|  |  | 4–8 | 0·88 | –0·1279 | –0·1465 | –0·1093 | 0·0056 | –22·6 | 1·4e-113 | 1631 (67·1%) | 201 812 (29·6%) |
|  |  | 8–12 (ref) |  |  |  |  |  |  |  | 2160 (88·9%) | 372 077 (54·5%) |
|  |  | 12–16 | 1·414 | 0·3462 | 0·3136 | 0·3787 | 0·0099 | 35 | 3·3e-268 | 967 (39·8%) | 43 713 (6·4%) |
|  |  | 16–20 | 1·234 | 0·2105 | 0·1627 | 0·2583 | 0·0145 | 14·5 | 1·1e-47 | 607 (25·0%) | 35 472 (5·2%) |
|  |  | 20–24 | 0·939 | –0·0627 | –0·1246 | –0·0007 | 0·0188 | –3·3 | 0·00088 | 280 (11·5%) | 25 608 (3·8%) |
| Disruptions | 7 days after data gap | Yes | 1·379 | 0·3213 | 0·275 | 0·3676 | 0·0141 | 22·8 | 1·7e-115 | 964 (39·7%) | 10 668 (1·6%) |
|  | 7 days after a holiday | Yes | 1·044 | 0·0428 | 0·0249 | 0·0608 | 0·0055 | 7·9 | 4e-15 | 2330 (95·9%) | 87 715 (12·8%) |
|  | After a missed dose | No recent non–adherence (ref) |  |  |  |  |  |  |  | 2423 (99·8%) | 561 939 (82·3%) |
|  |  | 1 day after ≤3 missed | 1·587 | 0·4621 | 0·4302 | 0·4941 | 0·0097 | 47·6 | 0·0 | 2115 (87·1%) | 23 188 (3·4%) |
|  |  | 2 days after ≤3 missed | 1·147 | 0·1375 | 0·1033 | 0·1716 | 0·0104 | 13·3 | 4e-40 | 2100 (86·5%) | 19 673 (2·9%) |
|  |  | 3 days after ≤3 missed | 1·061 | 0·0589 | 0·0229 | 0·0949 | 0·0109 | 5·4 | 7·3e-08 | 2093 (86·2%) | 17 303 (2·5%) |
|  |  | 4 days after ≤3 missed | 1·028 | 0·0274 | –0·0103 | 0·0651 | 0·0115 | 2·4 | 0·017 | 2082 (85·7%) | 15 539 (2·3%) |
|  |  | 5 days after ≤3 missed | 1·008 | 0·0084 | –0·0308 | 0·0477 | 0·0119 | 0·7 | 0·48 | 2073 (85·3%) | 14 166 (2·1%) |
|  |  | 6 days after ≤3 missed | 1·034 | 0·0334 | –0·0075 | 0·0743 | 0·0124 | 2·7 | 0·0072 | 2066 (85·1%) | 12 960 (1·9%) |
|  |  | 7 days after ≤3 missed | 1·055 | 0·054 | 0·0113 | 0·0967 | 0·013 | 4·2 | 3·2e-05 | 2052 (84·5%) | 11 763 (1·7%) |
|  |  | 1 day after >3 missed | 2·177 | 0·7779 | 0·6696 | 0·8862 | 0·0329 | 23·6 | 1·6e-123 | 720 (29·6%) | 1954 (0·3%) |
|  |  | 2 days after >3 missed | 1·577 | 0·4553 | 0·3143 | 0·5963 | 0·0429 | 10·6 | 2·3e-26 | 630 (25·9%) | 1057 (0·2%) |
|  |  | 3 days after >3 missed | 1·121 | 0·1146 | –0·046 | 0·2751 | 0·0488 | 2·3 | 0·019 | 566 (23·3%) | 810 (0·1%) |
|  |  | 4 days after >3 missed | 1·146 | 0·1363 | –0·0374 | 0·31 | 0·0528 | 2·6 | 0·0098 | 514 (21·2%) | 691 (0·1%) |
|  |  | 5 days after >3 missed | 1·051 | 0·0493 | –0·1349 | 0·2335 | 0·056 | 0·9 | 0·38 | 472 (19·4%) | 613 (0·1%) |
|  |  | 6 days after >3 missed | 1·007 | 0·007 | –0·1867 | 0·2007 | 0·0589 | 0·1 | 0·91 | 432 (17·8%) | 554 (0·1%) |
|  |  | 7 days after >3 missed | 1·036 | 0·0351 | –0·1646 | 0·2348 | 0·0607 | 0·6 | 0·56 | 416 (17·1%) | 521 (0·1%) |
| Other | Lost >5% weight | Yes | 0·912 | –0·092 | –0·2026 | 0·0185 | 0·0336 | –2·7 | 0·0062 | 580 (23·9%) | 165 275 (24·2%) |
|  | Covid-19 restrictions | Yes | 1·005 | 0·0052 | –0·0285 | 0·0389 | 0·0102 | 0·5 | 0·61 | 2158 (88·8%) | 484 294 (70·9%) |
|  | Intercept |  | 1205·67 | 7·0948 | 6·9134 | 7·2762 | 0·0551 | 128·7 | 0·0 | 2429 (100%) | 682 731 (100%) |

Results of the linear mixed effects regression model fit on the log-transformed dose consistency (the absolute value of adherence time differences on consecutive days). Variables with multiple values are dichotomized and reference values are indicated by (ref).

AE=adverse event; BMI=body mass index; CCS=Canadian Cardiovascular Society; CI=confidence interval; eGFR=estimated glomerular filtration rate; LVEF=left ventricular ejection fraction; NYHA=New York Heart Association; SE=standard error; STEMI=ST-elevation myocardial infarction.

### Supplementary Table 6. Results of Box–Cox-transformed dose consistency model

| Group | Feature | Value | Estimate | 0·05% CI | 99·95% CI | SE | t value | Pr(> \|t\|) | No. patients | No. observations |
| --- | --- | --- | --- | --- | --- | --- | --- | --- | --- | --- |
| Patient characteristics | **Country** | **Sweden** | –0·0156 | –0·1031 | 0·072 | 0·0266 | –0·6 | 0·56 | 534 (22·0%) | 178 078 (26·1%) |
|  | **Sex** | **Female** | –0·0498 | –0·1234 | 0·0237 | 0·0224 | –2·2 | 0·026 | 484 (19·9%) | 134 505 (19·7%) |
|  | **Age** | **<40** | 0·1328 | –0·078 | 0·3435 | 0·064 | 2·1 | 0·038 | 51 (2·1%) | 11 186 (1·6%) |
|  |  | **40–50** | 0·1451 | 0·0355 | 0·2548 | 0·0333 | 4·4 | 1·3e-05 | 228 (9·4%) | 57 877 (8·5%) |
|  |  | **50–60** | 0·1093 | 0·0351 | 0·1834 | 0·0225 | 4·8 | 1·3e-06 | 688 (28·3%) | 179 659 (26·3%) |
|  |  | **60–70 (ref)** |  |  |  |  |  |  | 813 (33·5%) | 241 157 (35·3%) |
|  |  | **70–80** | –0·1188 | –0·2006 | –0·037 | 0·0249 | –4·8 | 1·8e-06 | 547 (22·5%) | 163 909 (24·0%) |
|  |  | **>80** | –0·182 | –0·3378 | –0·0261 | 0·0474 | –3·8 | 0·00012 | 102 (4·2%) | 28 943 (4·2%) |
|  | **Race** | **White (ref)** |  |  |  |  |  |  | 2316 (95·3%) | 656 587 (96·2%) |
|  |  | **Asian** | 0·107 | –0·0637 | 0·2778 | 0·0519 | 2·1 | 0·039 | 77 (3·2%) | 18 382 (2·7%) |
|  |  | **Other** | 0·1189 | –0·133 | 0·3707 | 0·0765 | 1·6 | 0·12 | 36 (1·5%) | 7762 (1·1%) |
|  | **Arm** | **Dapagliflozin 10 mg** | –0·0125 | –0·0704 | 0·0453 | 0·0176 | –0·7 | 0·48 | 1201 (49·4%) | 339 180 (49·7%) |
|  | **Smoking status** | **Never (ref)** |  |  |  |  |  |  | 1044 (43·0%) | 305 880 (44·8%) |
|  |  | **Former** | 0·0292 | –0·0407 | 0·0992 | 0·0213 | 1·4 | 0·17 | 690 (28·4%) | 198 520 (29·1%) |
|  |  | **Current** | 0·1072 | 0·0343 | 0·1802 | 0·0222 | 4·8 | 1·3e-06 | 695 (28·6%) | 178 331 (26·1%) |
|  | **Hypertension history** | **Yes** | 0·0127 | –0·05 | 0·0755 | 0·0191 | 0·7 | 0·5 | 918 (37·8%) | 257 228 (37·7%) |
| Health status | **Systolic pressure** | **≥130** | 0·0309 | –0·0471 | 0·1089 | 0·0237 | 1·3 | 0·19 | 547 (22·5%) | 157 791 (23·1%) |
|  | **Diastolic pressure** | **≥80** | 0·0056 | –0·0698 | 0·0809 | 0·0229 | 0·2 | 0·81 | 595 (24·5%) | 170 256 (24·9%) |
|  | **BMI** | **≥30** | 0·0492 | –0·0144 | 0·1129 | 0·0193 | 2·5 | 0·011 | 772 (31·8%) | 211 257 (30·9%) |
|  | **LVEF** | **≥50 (ref)** |  |  |  |  |  |  | 505 (20·8%) | 113 901 (16·7%) |
|  |  | **30–49** | –0·0194 | –0·0927 | 0·0539 | 0·0223 | –0·9 | 0·38 | 1760 (72·5%) | 527 927 (77·3%) |
|  |  | **<30** | –0·0377 | –0·1697 | 0·0944 | 0·0401 | –0·9 | 0·35 | 164 (6·8%) | 40 903 (6·0%) |
|  | **eGFR** | **<60** | –0·0028 | –0·104 | 0·0985 | 0·0308 | –0·1 | 0·93 | 240 (9·9%) | 69 834 (10·2%) |
|  | **STEMI** | **STEMI** | –0·0048 | –0·073 | 0·0633 | 0·0207 | –0·2 | 0·82 | 1786 (73·5%) | 504 219 (73·9%) |
|  | **Revascularization** | **Yes** | –0·0188 | –0·0542 | 0·0166 | 0·0108 | –1·7 | 0·08 | 169 (7·0%) | 36 054 (5·3%) |
|  | **# medications** | **>12** | 0·0151 | –0·0461 | 0·0764 | 0·0186 | 0·8 | 0·42 | 1185 (48·8%) | 338 762 (49·6%) |
|  | **NYHA** | **No disease (ref)** |  |  |  |  |  |  | 1403 (57·8%) | 310 258 (45·4%) |
|  |  | **No symptoms** | 0·0137 | –0·006 | 0·0334 | 0·006 | 2·3 | 0·022 | 1136 (46·8%) | 233 131 (34·1%) |
|  |  | **Mild symptoms** | 0·0182 | –0·0063 | 0·0427 | 0·0074 | 2·4 | 0·015 | 599 (24·7%) | 121 357 (17·8%) |
|  |  | **Marked/Severe** | 0·0295 | –0·0221 | 0·081 | 0·0157 | 1·9 | 0·06 | 122 (5·0%) | 17 985 (2·6%) |
|  | **CCS Angina grade** | **Asymptomatic (ref)** |  |  |  |  |  |  | 2037 (83·9%) | 519 025 (76·0%) |
|  |  | **Strenuous exertion** | 0·0069 | –0·0161 | 0·0299 | 0·007 | 1 | 0·33 | 653 (26·9%) | 127 845 (18·7%) |
|  |  | **Moderate exertion** | –0·0427 | –0·0876 | 0·0021 | 0·0136 | –3·1 | 0·0017 | 144 (5·9%) | 27 928 (4·1%) |
|  |  | **Mild exertion/At rest** | –0·1085 | –0·1859 | –0·0311 | 0·0235 | –4·6 | 4e-06 | 50 (2·1%) | 7933 (1·2%) |
| Calendar features | **Days from start** | **≤30 (ref)** |  |  |  |  |  |  | 2398 (98·7%) | 66 243 (9·7%) |
|  |  | **31–60** | –0·0023 | –0·0184 | 0·0138 | 0·0049 | –0·5 | 0·64 | 2340 (96·3%) | 64 710 (9·5%) |
|  |  | **61–90** | 0·0237 | 0·0072 | 0·0401 | 0·005 | 4·7 | 2·1e-06 | 2285 (94·1%) | 62 252 (9·1%) |
|  |  | **91–120** | 0·055 | 0·0379 | 0·072 | 0·0052 | 10·6 | 2·2e-26 | 2172 (89·4%) | 56 770 (8·3%) |
|  |  | **121–150** | 0·0647 | 0·047 | 0·0824 | 0·0054 | 12 | 3·2e-33 | 1931 (79·5%) | 50 901 (7·5%) |
|  |  | **151–200** | 0·0814 | 0·0649 | 0·0979 | 0·005 | 16·2 | 2·9e-59 | 1777 (73·2%) | 75 440 (11·0%) |
|  |  | **201–250** | 0·0794 | 0·0619 | 0·0969 | 0·0053 | 14·9 | 2·5e-50 | 1530 (63·0%) | 64 397 (9·4%) |
|  |  | **251–300** | 0·0862 | 0·0676 | 0·1048 | 0·0056 | 15·3 | 9·9e-53 | 1323 (54·5%) | 56 955 (8·3%) |
|  |  | **301–350** | 0·1021 | 0·0821 | 0·122 | 0·0061 | 16·8 | 1·1e-63 | 1180 (48·6%) | 48 257 (7·1%) |
|  |  | **351–400** | 0·107 | 0·0853 | 0·1287 | 0·0066 | 16·2 | 3·8e-59 | 947 (39·0%) | 38 203 (5·6%) |
|  |  | **401–450** | 0·1138 | 0·0902 | 0·1374 | 0·0072 | 15·9 | 7·9e-57 | 745 (30·7%) | 31 878 (4·7%) |
|  |  | **451–500** | 0·1231 | 0·0971 | 0·149 | 0·0079 | 15·6 | 5·7e-55 | 609 (25·1%) | 25 478 (3·7%) |
|  |  | **>500** | 0·1212 | 0·0957 | 0·1467 | 0·0078 | 15·6 | 4·9e-55 | 475 (19·6%) | 41 247 (6·0%) |
|  | **Month** | **January (ref)** |  |  |  |  |  |  | 2036 (83·8%) | 72 361 (10·6%) |
|  |  | **February** | –0·0083 | –0·0239 | 0·0074 | 0·0048 | –1·7 | 0·083 | 2131 (87·7%) | 70 121 (10·3%) |
|  |  | **March** | –0·0206 | –0·0425 | 0·0013 | 0·0067 | –3·1 | 0·002 | 2158 (88·8%) | 78 699 (11·5%) |
|  |  | **April** | –0·0085 | –0·0302 | 0·0131 | 0·0066 | –1·3 | 0·2 | 2047 (84·3%) | 60 560 (8·9%) |
|  |  | **May** | 0·0217 | –0·0004 | 0·0439 | 0·0067 | 3·2 | 0·0012 | 1702 (70·1%) | 43 762 (6·4%) |
|  |  | **June** | 0·0284 | 0·0056 | 0·0512 | 0·0069 | 4·1 | 4·3e-05 | 1353 (55·7%) | 37 157 (5·4%) |
|  |  | **July** | 0·017 | –0·0056 | 0·0396 | 0·0069 | 2·5 | 0·013 | 1385 (57·0%) | 41 867 (6·1%) |
|  |  | **August** | 0·0008 | –0·0216 | 0·0232 | 0·0068 | 0·1 | 0·91 | 1469 (60·5%) | 45 242 (6·6%) |
|  |  | **September** | –0·0121 | –0·0343 | 0·0101 | 0·0067 | –1·8 | 0·073 | 1602 (66·0%) | 48 611 (7·1%) |
|  |  | **October** | –0·0062 | –0·0283 | 0·0159 | 0·0067 | –0·9 | 0·35 | 1712 (70·5%) | 56 396 (8·3%) |
|  |  | **November** | –0·018 | –0·0399 | 0·0039 | 0·0067 | –2·7 | 0·0068 | 1829 (75·3%) | 60 133 (8·8%) |
|  |  | **December** | 0·0214 | –0·0003 | 0·0431 | 0·0066 | 3·3 | 0·0012 | 1940 (79·9%) | 67 822 (9·9%) |
|  | **Days of the week** | **Monday** | 0·2952 | 0·2821 | 0·3084 | 0·004 | 74 | 0·0 | 2427 (99·9%) | 97 874 (14·3%) |
|  |  | **Tuesday** | 0·0367 | 0·0237 | 0·0496 | 0·0039 | 9·3 | 1·5e-20 | 2424 (99·8%) | 97 897 (14·3%) |
|  |  | **Wednesday (ref)** |  |  |  |  |  |  | 2427 (99·9%) | 97 656 (14·3%) |
|  |  | **Thursday** | 0·0105 | –0·0025 | 0·0235 | 0·0039 | 2·7 | 0·0079 | 2427 (99·9%) | 97 682 (14·3%) |
|  |  | **Friday** | 0·0402 | 0·0272 | 0·0532 | 0·004 | 10·2 | 3·1e-24 | 2428 (100·0%) | 97 473 (14·3%) |
|  |  | **Saturday** | 0·2452 | 0·2322 | 0·2583 | 0·004 | 61·9 | 0·0 | 2426 (99·9%) | 96 995 (14·2%) |
|  |  | **Sunday** | 0·1706 | 0·1575 | 0·1836 | 0·004 | 42·9 | 0·0 | 2424 (99·8%) | 97 154 (14·2%) |
|  | **Holiday** | **Holiday** | 0·0079 | –0·011 | 0·0268 | 0·0057 | 1·4 | 0·17 | 2316 (95·3%) | 26 576 (3·9%) |
|  | **Study Closeout** | **Yes** | 0·0197 | –0·0024 | 0·0418 | 0·0067 | 2·9 | 0·0034 | 2045 (84·2%) | 97 440 (14·3%) |
| Adverse events | **Hospitalized or discontinued** | **No AE (ref)** |  |  |  |  |  |  | 2402 (98·9%) | 645 937 (94·6%) |
|  |  | **Neither** | 0·0909 | 0·0555 | 0·1263 | 0·0108 | 8·4 | 3·1e-17 | 277 (11·4%) | 27 072 (4·0%) |
|  |  | **Discontinued only** | 0·0999 | –0·0161 | 0·2159 | 0·0353 | 2·8 | 0·0046 | 40 (1·6%) | 1260 (0·2%) |
|  |  | **Hospitalized only** | 0·1326 | 0·0831 | 0·1821 | 0·015 | 8·8 | 1·2e-18 | 288 (11·9%) | 6230 (0·9%) |
|  |  | **Both** | 0·1248 | 0·0372 | 0·2125 | 0·0266 | 4·7 | 2·8e-06 | 101 (4·2%) | 2232 (0·3%) |
|  | **7 days after AE** | **No AE (ref)** |  |  |  |  |  |  | 2429 (100·0%) | 678 868 (99·4%) |
|  |  | **Not hospitalized or discontinued** | –0·0061 | –0·0862 | 0·0739 | 0·0243 | –0·3 | 0·8 | 176 (7·2%) | 1339 (0·2%) |
|  |  | **Not hospitalized but discontinued** | 0·3335 | –0·018 | 0·6851 | 0·1068 | 3·1 | 0·0018 | 11 (0·5%) | 69 (0·0%) |
|  |  | **Hospitalized, not discontinued** | 0·019 | –0·046 | 0·084 | 0·0198 | 1 | 0·34 | 263 (10·8%) | 2053 (0·3%) |
|  |  | **Hospitalized and discontinued** | 0·0854 | –0·0603 | 0·2312 | 0·0443 | 1·9 | 0·054 | 67 (2·8%) | 402 (0·1%) |
| Behavioural features | **Mobile application use** | **Yes** | 0·0625 | 0·0105 | 0·1145 | 0·0158 | 4 | 7·7e-05 | 647 (26·6%) | 3130 (0·5%) |
|  | **7 days after mobile application use** | **Yes** | 0·0071 | –0·014 | 0·0281 | 0·0064 | 1·1 | 0·27 | 682 (28·1%) | 22447 (3·3%) |
|  | **Off-schedule use in last 7 days** | **Yes** | 0·068 | 0·0551 | 0·0809 | 0·0039 | 17·3 | 2·8e-67 | 2253 (92·8%) | 60 719 (8·9%) |
|  | **Average time in last 7 days** | **0–4** | 0·0789 | 0·0249 | 0·133 | 0·0164 | 4·8 | 1·5e-06 | 181 (7·5%) | 4049 (0·6%) |
|  |  | **4–8** | –0·0757 | –0·0874 | –0·064 | 0·0036 | –21·2 | 6e-100 | 1631 (67·1%) | 201 812 (29·6%) |
|  |  | **8–12 (ref)** |  |  |  |  |  |  | 2160 (88·9%) | 372 077 (54·5%) |
|  |  | **12–16** | 0·2626 | 0·242 | 0·2832 | 0·0062 | 42 | 0·0 | 967 (39·8%) | 43 713 (6·4%) |
|  |  | **16–20** | 0·1611 | 0·131 | 0·1912 | 0·0092 | 17·6 | 2·9e-69 | 607 (25·0%) | 35 472 (5·2%) |
|  |  | **20–24** | –0·0586 | –0·0977 | –0·0195 | 0·0119 | –4·9 | 8·2e-07 | 280 (11·5%) | 25 608 (3·8%) |
| Disruptions | **7 days after data gap** | **Yes** | 0·2374 | 0·2082 | 0·2666 | 0·0089 | 26·7 | 3e-157 | 964 (39·7%) | 10 668 (1·6%) |
|  | **7 days after a holiday** | **Yes** | 0·0261 | 0·0148 | 0·0374 | 0·0034 | 7·6 | 3·4e-14 | 2330 (95·9%) | 87 715 (12·8%) |
|  | **After a missed dose** | **No recent non–adherence (ref)** |  |  |  |  |  |  | 2423 (99·8%) | 561 939 (82·3%) |
|  |  | **1 day after ≤3 missed** | 0·3532 | 0·333 | 0·3734 | 0·0061 | 57·6 | 0·0 | 2115 (87·1%) | 23 188 (3·4%) |
|  |  | **2 days after ≤3 missed** | 0·1027 | 0·0812 | 0·1243 | 0·0065 | 15·7 | 2e-55 | 2100 (86·5%) | 19 673 (2·9%) |
|  |  | **3 days after ≤3 missed** | 0·0425 | 0·0197 | 0·0652 | 0·0069 | 6·1 | 7·8e-10 | 2093 (86·2%) | 17 303 (2·5%) |
|  |  | **4 days after ≤3 missed** | 0·0173 | –0·0065 | 0·0411 | 0·0072 | 2·4 | 0·017 | 2082 (85·7%) | 15 539 (2·3%) |
|  |  | **5 days after ≤3 missed** | 0·0058 | –0·019 | 0·0306 | 0·0075 | 0·8 | 0·44 | 2073 (85·3%) | 14 166 (2·1%) |
|  |  | **6 days after ≤3 missed** | 0·0205 | –0·0053 | 0·0464 | 0·0078 | 2·6 | 0·0089 | 2066 (85·1%) | 12 960 (1·9%) |
|  |  | **7 days after ≤3 missed** | 0·0363 | 0·0093 | 0·0633 | 0·0082 | 4·4 | 9·7e-06 | 2052 (84·5%) | 11 763 (1·7%) |
|  |  | **1 day after >3 missed** | 0·5821 | 0·5137 | 0·6505 | 0·0208 | 28 | 1·4e-172 | 720 (29·6%) | 1954 (0·3%) |
|  |  | **2 days after >3 missed** | 0·325 | 0·2359 | 0·4141 | 0·0271 | 12 | 3·3e-33 | 630 (25·9%) | 1057 (0·2%) |
|  |  | **3 days after >3 missed** | 0·0806 | –0·0209 | 0·182 | 0·0308 | 2·6 | 0·009 | 566 (23·3%) | 810 (0·1%) |
|  |  | **4 days after >3 missed** | 0·0964 | –0·0134 | 0·2061 | 0·0333 | 2·9 | 0·0039 | 514 (21·2%) | 691 (0·1%) |
|  |  | **5 days after >3 missed** | 0·0401 | –0·0762 | 0·1565 | 0·0354 | 1·1 | 0·26 | 472 (19·4%) | 613 (0·1%) |
|  |  | **6 days after >3 missed** | –0·001 | –0·1233 | 0·1214 | 0·0372 | 0 | 0·98 | 432 (17·8%) | 554 (0·1%) |
|  |  | **7 days after >3 missed** | 0·028 | –0·0981 | 0·1542 | 0·0383 | 0·7 | 0·46 | 416 (17·1%) | 521 (0·1%) |
| Other | **Lost >5% weight** | **Yes** | –0·058 | –0·1265 | 0·0105 | 0·0208 | –2·8 | 0·0053 | 580 (23·9%) | 165 275 (24·2%) |
|  | **COVID-19 restrictions** | **Yes** | 0·0057 | –0·0156 | 0·027 | 0·0065 | 0·9 | 0·38 | 2158 (88·8%) | 484 294 (70·9%) |
|  | **Intercept** |  | –0·2301 | –0·3427 | –0·1174 | 0·0342 | –6·7 | 1·8e-11 | 2429 (100%) | 682 731 (100%) |

Results of the linear mixed effects regression model fit on the Box-Cox-transformed dose consistency (the absolute value of adherence time differences on consecutive days). Variables with multiple values are dichotomized and reference values are indicated by (ref). Error bars represent 99·95% CIs (see corresponding Figure 6 and random effects table in Table 7).

AE=adverse event; BMI=body mass index; CCS=Canadian Cardiovascular Society; CI=confidence interval; LVEF=left ventricular ejection fraction; NYHA=New York Heart Association; SE=standard error; STEMI=ST-elevation myocardial infarction.

### Supplementary Table 7. Results of adherence model for the anxiety subset

| Feature | Value | Odds ratio | Estimate | 0·05% CI | 99·95% CI | SE | z value | Pr(>\|z\|) | No. patients | No. observations |
| --- | --- | --- | --- | --- | --- | --- | --- | --- | --- | --- |
| Country | **Sweden** | 1·085 | 0·0818 | –0·3733 | 0·5369 | 0·1383 | 0·6 | 0·55 | 504 (45·0%) | 182 948 (47·4%) |
| Sex | **Female** | 1·363 | 0·3098 | –0·0802 | 0·6997 | 0·1185 | 2·6 | 0·009 | 196 (17·5%) | 69 977 (18·1%) |
| Age | **<40** | 0·286 | –1·2526 | –2·3483 | –0·1569 | 0·333 | -3·8 | 0·00017 | 19 (1·7%) | 5186 (1·3%) |
|  | **40–50** | 0·731 | –0·3137 | –0·8533 | 0·2259 | 0·164 | -1·9 | 0·056 | 102 (9·1%) | 33 664 (8·7%) |
|  | **50–60** | 0·729 | –0·3157 | –0·6795 | 0·0482 | 0·1106 | -2·9 | 0·0043 | 309 (27·6%) | 101 254 (26·2%) |
|  | **60–70 (ref)** |  |  |  |  |  |  |  | 383 (34·2%) | 136 464 (35·4%) |
|  | **70–80** | 0·882 | –0·1255 | –0·5301 | 0·2792 | 0·123 | -1 | 0·31 | 255 (22·8%) | 91 930 (23·8%) |
|  | **>80** | 0·786 | –0·2408 | –0·9954 | 0·5137 | 0·2293 | -1·1 | 0·29 | 52 (4·6%) | 17 420 (4·5%) |
| Race | **White (ref)** |  |  |  |  |  |  |  | 1073 (95·8%) | 371 936 (96·4%) |
|  | **Asian** | 1·126 | 0·1183 | –0·7996 | 1·0363 | 0·279 | 0·4 | 0·67 | 28 (2·5%) | 9181 (2·4%) |
|  | **Other** | 0·459 | –0·7787 | –2·0833 | 0·526 | 0·3965 | -2 | 0·05 | 19 (1·7%) | 4801 (1·2%) |
| Arm | **Dapagliflozin 10 mg** | 0·976 | –0·0239 | –0·3098 | 0·262 | 0·0869 | -0·3 | 0·78 | 544 (48·6%) | 187 717 (48·6%) |
| Smoking status | **Never (ref)** |  |  |  |  |  |  |  | 520 (46·4%) | 180 245 (46·7%) |
|  | **Former** | 1·069 | 0·0663 | –0·2702 | 0·4029 | 0·1023 | 0·6 | 0·52 | 345 (30·8%) | 121 227 (31·4%) |
|  | **Current** | 0·648 | –0·4337 | –0·8052 | –0·0621 | 0·1129 | -3·8 | 0·00012 | 255 (22·8%) | 84 446 (21·9%) |
| Hypertension history | **Yes** | 0·969 | –0·032 | –0·3372 | 0·2733 | 0·0928 | -0·3 | 0·73 | 443 (39·6%) | 153 487 (39·8%) |
| Systolic pressure | **≥130** | 1·013 | 0·0131 | –0·362 | 0·3882 | 0·114 | 0·1 | 0·91 | 271 (24·2%) | 96 328 (25·0%) |
| Diastolic pressure | **≥80** | 1·001 | 0·0013 | –0·3588 | 0·3614 | 0·1094 | 0 | 0·99 | 311 (27·8%) | 109 963 (28·5%) |
| BMI | **≥30** | 0·966 | –0·0342 | –0·3548 | 0·2865 | 0·0975 | -0·4 | 0·73 | 330 (29·5%) | 113 380 (29·4%) |
| LVEF | **≥50 (ref)** |  |  |  |  |  |  |  | 243 (21·7%) | 64 612 (16·7%) |
|  | **30–49** | 0·96 | –0·0412 | –0·3992 | 0·3169 | 0·1088 | -0·4 | 0·71 | 817 (72·9%) | 301 992 (78·3%) |
|  | **<30** | 1·05 | 0·0484 | –0·6578 | 0·7546 | 0·2146 | 0·2 | 0·82 | 60 (5·4%) | 19 314 (5·0%) |
| eGFR | **<60** | 0·984 | –0·0164 | –0·5376 | 0·5048 | 0·1584 | -0·1 | 0·92 | 98 (8·8%) | 36 361 (9·4%) |
| STEMI | **STEMI** | 1·321 | 0·2781 | –0·0517 | 0·6078 | 0·1002 | 2·8 | 0·0055 | 824 (73·6%) | 285 349 (73·9%) |
| Revascularization | **Yes** | 0·79 | –0·2361 | –0·5034 | 0·0311 | 0·0812 | -2·9 | 0·0036 | 69 (6·2%) | 17 287 (4·5%) |
| # medications | **>12** | 0·918 | –0·0852 | –0·3879 | 0·2176 | 0·092 | -0·9 | 0·35 | 473 (42·2%) | 170 859 (44·3%) |
| NYHA | **No disease (ref)** |  |  |  |  |  |  |  | 744 (66·4%) | 193 265 (50·1%) |
|  | **No symptoms** | 0·97 | –0·0301 | –0·1736 | 0·1134 | 0·0436 | -0·7 | 0·49 | 494 (44·1%) | 115 036 (29·8%) |
|  | **Mild symptoms** | 1·225 | 0·2027 | 0·0401 | 0·3654 | 0·0494 | 4·1 | 4·1e-05 | 295 (26·3%) | 69 903 (18·1%) |
|  | **Marked/Severe** | 1·22 | 0·199 | –0·1485 | 0·5466 | 0·1056 | 1·9 | 0·059 | 44 (3·9%) | 7714 (2·0%) |
| CCS Angina grade | **Asymptomatic (ref)** |  |  |  |  |  |  |  | 995 (88·8%) | 311 785 (80·8%) |
|  | **Strenuous exertion** | 1·073 | 0·0701 | –0·0996 | 0·2398 | 0·0516 | 1·4 | 0·17 | 257 (22·9%) | 56 220 (14·6%) |
|  | **Moderate exertion** | 0·954 | –0·047 | –0·3319 | 0·2379 | 0·0866 | -0·5 | 0·59 | 60 (5·4%) | 15 252 (4·0%) |
|  | **Mild exertion/At rest** | 1·15 | 0·1401 | –0·7472 | 1·0273 | 0·2696 | 0·5 | 0·6 | 16 (1·4%) | 2661 (0·7%) |
| Days from start | **≤30 (ref)** |  |  |  |  |  |  |  | 1111 (99·2%) | 33 524 (8·7%) |
|  | **31–60** | 0·963 | –0·0376 | –0·174 | 0·0987 | 0·0414 | -0·9 | 0·36 | 1093 (97·6%) | 32 283 (8·4%) |
|  | **61–90** | 0·812 | –0·2082 | –0·3433 | –0·0731 | 0·0411 | -5·1 | 3·9e-07 | 1076 (96·1%) | 31 773 (8·2%) |
|  | **91–120** | 0·704 | –0·3515 | –0·4882 | –0·2147 | 0·0416 | -8·5 | 2·7e-17 | 1049 (93·7%) | 29 721 (7·7%) |
|  | **121–150** | 0·599 | –0·5132 | –0·6516 | –0·3749 | 0·042 | -12·2 | 2·8e-34 | 951 (84·9%) | 27 173 (7·0%) |
|  | **151–200** | 0·583 | –0·5404 | –0·67 | –0·4107 | 0·0394 | -13·7 | 8·7e-43 | 880 (78·6%) | 40 938 (10·6%) |
|  | **201–250** | 0·634 | –0·4559 | –0·5917 | –0·3201 | 0·0413 | -11 | 2·2e-28 | 786 (70·2%) | 36 391 (9·4%) |
|  | **251–300** | 0·62 | –0·4786 | –0·6196 | –0·3377 | 0·0428 | -11·2 | 5·5e-29 | 716 (63·9%) | 33 136 (8·6%) |
|  | **301–350** | 0·604 | –0·5034 | –0·6512 | –0·3556 | 0·0449 | -11·2 | 3·8e-29 | 647 (57·8%) | 29 037 (7·5%) |
|  | **351–400** | 0·68 | –0·3852 | –0·5454 | –0·225 | 0·0487 | -7·9 | 2·5e-15 | 539 (48·1%) | 23 504 (6·1%) |
|  | **401–450** | 0·694 | –0·3646 | –0·5378 | –0·1914 | 0·0526 | -6·9 | 4·3e-12 | 441 (39·4%) | 20 180 (5·2%) |
|  | **451–500** | 0·592 | –0·5249 | –0·7077 | –0·342 | 0·0556 | -9·4 | 3·5e-21 | 367 (32·8%) | 16 657 (4·3%) |
|  | **>500** | 0·601 | –0·5084 | –0·6898 | –0·327 | 0·0551 | -9·2 | 2·9e-20 | 295 (26·3%) | 31 601 (8·2%) |
| Month | **January (ref)** |  |  |  |  |  |  |  | 1000 (89·3%) | 40 045 (10·4%) |
|  | **February** | 1·005 | 0·0049 | –0·1128 | 0·1227 | 0·0358 | 0·1 | 0·89 | 1035 (92·4%) | 38 966 (10·1%) |
|  | **March** | 1·125 | 0·1175 | –0·0412 | 0·2762 | 0·0482 | 2·4 | 0·015 | 1041 (92·9%) | 43 873 (11·4%) |
|  | **April** | 1·017 | 0·017 | –0·1388 | 0·1728 | 0·0474 | 0·4 | 0·72 | 992 (88·6%) | 33 530 (8·7%) |
|  | **May** | 0·998 | –0·0018 | –0·1621 | 0·1585 | 0·0487 | 0 | 0·97 | 831 (74·2%) | 24 612 (6·4%) |
|  | **June** | 0·995 | –0·0047 | –0·169 | 0·1596 | 0·0499 | -0·1 | 0·92 | 705 (62·9%) | 22 220 (5·8%) |
|  | **July** | 0·925 | –0·078 | –0·2391 | 0·0831 | 0·049 | -1·6 | 0·11 | 717 (64·0%) | 24 591 (6·4%) |
|  | **August** | 0·903 | –0·1024 | –0·2622 | 0·0574 | 0·0486 | -2·1 | 0·035 | 768 (68·6%) | 26 424 (6·8%) |
|  | **September** | 0·928 | –0·0749 | –0·2343 | 0·0844 | 0·0484 | -1·5 | 0·12 | 819 (73·1%) | 28 043 (7·3%) |
|  | **October** | 1·013 | 0·013 | –0·1472 | 0·1733 | 0·0487 | 0·3 | 0·79 | 862 (77·0%) | 32 079 (8·3%) |
|  | **November** | 0·905 | –0·0996 | –0·2563 | 0·0572 | 0·0476 | -2·1 | 0·037 | 911 (81·3%) | 33 856 (8·8%) |
|  | **December** | 0·887 | –0·1202 | –0·2749 | 0·0345 | 0·047 | -2·6 | 0·011 | 955 (85·3%) | 37 679 (9·8%) |
| Days of the week | **Monday** | 0·97 | –0·03 | –0·1266 | 0·0665 | 0·0293 | -1 | 0·31 | 1120 (100·0%) | 55 147 (14·3%) |
|  | **Tuesday** | 1·01 | 0·0095 | –0·0869 | 0·1058 | 0·0293 | 0·3 | 0·75 | 1120 (100·0%) | 55 207 (14·3%) |
|  | **Wednesday (ref)** |  |  |  |  |  |  |  | 1120 (100·0%) | 55 165 (14·3%) |
|  | **Thursday** | 0·99 | –0·01 | –0·1062 | 0·0861 | 0·0292 | -0·3 | 0·73 | 1120 (100·0%) | 55 112 (14·3%) |
|  | **Friday** | 0·929 | –0·0731 | –0·1684 | 0·0222 | 0·029 | -2·5 | 0·012 | 1120 (100·0%) | 55 168 (14·3%) |
|  | **Saturday** | 0·815 | –0·2041 | –0·2974 | –0·1108 | 0·0284 | -7·2 | 6·2e-13 | 1120 (100·0%) | 55 059 (14·3%) |
|  | **Sunday** | 0·825 | –0·1919 | –0·2857 | –0·098 | 0·0285 | -6·7 | 1·7e-11 | 1120 (100·0%) | 55 060 (14·3%) |
| Holiday | **Holiday** | 0·917 | –0·0871 | –0·2161 | 0·042 | 0·0392 | -2·2 | 0·026 | 1096 (97·9%) | 15 531 (4·0%) |
| Study Closeout | **Yes** | 0·591 | –0·5257 | –0·6848 | –0·3665 | 0·0484 | -10·9 | 1·6e-27 | 982 (87·7%) | 49 707 (12·9%) |
| Hospitalized or discontinued | **No AE (ref)** |  |  |  |  |  |  |  | 1114 (99·5%) | 367 483 (95·2%) |
|  | **Neither** | 0·849 | –0·1638 | –0·4816 | 0·1539 | 0·0966 | -1·7 | 0·09 | 101 (9·0%) | 12 845 (3·3%) |
|  | **Discontinued only** | 0·116 | –2·1567 | –2·7589 | –1·5545 | 0·183 | -11·8 | 4·7e-32 | 19 (1·7%) | 696 (0·2%) |
|  | **Hospitalized only** | 0·302 | –1·1981 | –1·4694 | –0·9269 | 0·0824 | -14·5 | 7·5e-48 | 149 (13·3%) | 3987 (1·0%) |
|  | **Both** | 0·183 | –1·6982 | –2·0948 | –1·3016 | 0·1205 | -14·1 | 4·4e-45 | 47 (4·2%) | 907 (0·2%) |
| 7 days after AE | **No AE (ref)** |  |  |  |  |  |  |  | 1120 (100·0%) | 384 037 (99·5%) |
|  | **Not hospitalized or discontinued** | 1·119 | 0·1125 | –0·6078 | 0·8329 | 0·2189 | 0·5 | 0·61 | 60 (5·4%) | 548 (0·1%) |
|  | **Not hospitalized but discontinued** | 0·578 | –0·5487 | –3·9324 | 2·835 | 1·0283 | -0·5 | 0·59 | 5 (0·4%) | 33 (0·0%) |
|  | **Hospitalized, not discontinued** | 0·719 | –0·3292 | –0·7678 | 0·1094 | 0·1333 | -2·5 | 0·014 | 133 (11·9%) | 1093 (0·3%) |
|  | **Hospitalized and discontinued** | 0·283 | –1·2633 | –1·9247 | –0·6018 | 0·201 | -6·3 | 3·3e-10 | 30 (2·7%) | 207 (0·1%) |
| Mobile application use | **Yes** | 1·387 | 0·3273 | 0·0036 | 0·6509 | 0·0984 | 3·3 | 0·00088 | 684 (61·1%) | 3375 (0·9%) |
| 7 days after mobile application use | **Yes** | 1·243 | 0·2179 | 0·0935 | 0·3423 | 0·0378 | 5·8 | 8·2e-09 | 685 (61·2%) | 23 471 (6·1%) |
| Off-schedule use in last 7 days | **Yes** | 0·52 | –0·6531 | –0·7255 | –0·5806 | 0·022 | -29·7 | 2·7e-193 | 1062 (94·8%) | 35 953 (9·3%) |
| Average time in last 7 days | **0–4** | 1·097 | 0·0926 | –0·2681 | 0·4534 | 0·1096 | 0·8 | 0·4 | 113 (10·1%) | 2617 (0·7%) |
|  | **4–8** | 1·252 | 0·2248 | 0·1419 | 0·3077 | 0·0252 | 8·9 | 4·6e-19 | 868 (77·5%) | 134 248 (34·8%) |
|  | **8–12 (ref)** |  |  |  |  |  |  |  | 1031 (92·1%) | 201 598 (52·2%) |
|  | **12–16** | 0·816 | –0·2028 | –0·3172 | –0·0884 | 0·0348 | -5·8 | 5·4e-09 | 418 (37·3%) | 21 302 (5·5%) |
|  | **16–20** | 0·83 | –0·1868 | –0·3464 | –0·0272 | 0·0485 | -3·9 | 0·00012 | 261 (23·3%) | 15 119 (3·9%) |
|  | **20–24** | 0·501 | –0·6913 | –0·8979 | –0·4847 | 0·0628 | -11 | 3·4e-28 | 121 (10·8%) | 11 034 (2·9%) |
| Average time variance in last 7 days | **<15 min** | 1·696 | 0·5285 | 0·3771 | 0·68 | 0·046 | 11·5 | 1·6e-30 | 702 (62·7%) | 34 906 (9·0%) |
|  | **15–30 min** | 1·668 | 0·5115 | 0·4024 | 0·6207 | 0·0332 | 15·4 | 1·1e-53 | 1019 (91·0%) | 66 635 (17·3%) |
|  | **30–60 min** | 1·444 | 0·3677 | 0·2835 | 0·4519 | 0·0256 | 14·4 | 8·7e-47 | 1092 (97·5%) | 123 018 (31·9%) |
|  | **1–2 h** | 1·285 | 0·251 | 0·1755 | 0·3266 | 0·023 | 10·9 | 7·9e-28 | 1081 (96·5%) | 102 973 (26·7%) |
|  | **>2 h (ref)** |  |  |  |  |  |  |  | 903 (80·6%) | 58 386 (15·1%) |
| 7 days after data gap | **Yes** | 0·957 | –0·044 | –0·2183 | 0·1303 | 0·053 | -0·8 | 0·41 | 497 (44·4%) | 6426 (1·7%) |
| 7 days after a holiday | **Yes** | 1·022 | 0·022 | –0·0592 | 0·1032 | 0·0247 | 0·9 | 0·37 | 1095 (97·8%) | 51 929 (13·5%) |
| Lost >5% weight | **Yes** | 0·92 | –0·083 | –0·4178 | 0·2518 | 0·1017 | -0·8 | 0·41 | 277 (24·7%) | 94 069 (24·4%) |
| COVID-19 restrictions | **Yes** | 0·969 | –0·0313 | –0·1844 | 0·1217 | 0·0465 | -0·7 | 0·5 | 1020 (91·1%) | 282 864 (73·3%) |
| EQ-5D Anxiety | **No (ref)** |  |  |  |  |  |  |  | 851 (76·0%) | 251 594 (65·2%) |
|  | **Moderate (3L)** | 1·012 | 0·0122 | –0·1966 | 0·2211 | 0·0635 | 0·2 | 0·85 | 155 (13·8%) | 40 156 (10·4%) |
|  | **Extreme (3L)** | 1·871 | 0·6265 | 0·0166 | 1·2365 | 0·1854 | 3·4 | 0·00072 | 12 (1·1%) | 3681 (1·0%) |
|  | **Slight (5L)** | 0·943 | –0·0583 | –0·2 | 0·0833 | 0·043 | -1·4 | 0·18 | 356 (31·8%) | 67 569 (17·5%) |
|  | **Moderate (5L)** | 1·079 | 0·0763 | –0·1618 | 0·3143 | 0·0723 | 1·1 | 0·29 | 125 (11·2%) | 17 626 (4·6%) |
|  | **Severe/Extreme (5L)** | 1·632 | 0·4896 | 0·0752 | 0·9041 | 0·126 | 3·9 | 0·0001 | 28 (2·5%) | 5292 (1·4%) |
| Intercept |  | 48·768 | 3·8871 | 3·2999 | 4·4743 | 0·1784 | 21·8 | 3·4e-105 | 1120 (100%) | 385 918 (100%) |

Results of the logistic mixed effects regression model fit on the subset of adherence dataset including only patients who provided responses to EQ-5D Anxiety/Depression question. Variables with multiple values are dichotomized and reference values are indicated by (ref). Error bars represent 99·95% confidence intervals (see corresponding Supplementary Figure 5).

AE=adverse event; BMI=body mass index; CCS=Canadian Cardiovascular Society; CI=confidence interval; eGFR=estimated glomerular filtration rate; LVEF=left ventricular ejection fraction; NYHA=New York Heart Association; SE=standard error; STEMI=ST-elevation myocardial infarction.

### Supplementary Table 8. Results of Box–Cox-transformed dose consistency for the anxiety subset

| Value | Estimate | 0·05% CI | 99·95% CI | SE | t value | Pr(>\|t\|) | No. patients | No. observations |
| --- | --- | --- | --- | --- | --- | --- | --- | --- |
| Sweden | 0·0339 | –0·068 | 0·1358 | 0·031 | 1·1 | 0·27 | 504 (45·0%) | 173 118 (47·7%) |
| Female | –0·0925 | –0·1993 | 0·0143 | 0·0325 | –2·9 | 0·0044 | 196 (17·5%) | 66 512 (18·3%) |
| <40 | 0·1303 | –0·1893 | 0·45 | 0·0971 | 1·3 | 0·18 | 19 (1·7%) | 4691 (1·3%) |
| 40–50 | 0·1833 | 0·0312 | 0·3354 | 0·0462 | 4 | 7·3e-05 | 102 (9·1%) | 31 439 (8·7%) |
| 50–60 | 0·1504 | 0·0484 | 0·2525 | 0·031 | 4·9 | 1·2e-06 | 309 (27·6%) | 93 885 (25·9%) |
| 60–70 (ref) |  |  |  |  |  |  | 383 (34·2%) | 129 462 (35·7%) |
| 70–80 | –0·102 | –0·2141 | 0·01 | 0·0341 | –3 | 0·0027 | 255 (22·8%) | 87 047 (24·0%) |
| >80 | –0·1482 | –0·3559 | 0·0595 | 0·0631 | –2·3 | 0·019 | 52 (4·6%) | 16 336 (4·5%) |
| White (ref) |  |  |  |  |  |  | 1073 (95·8%) | 350 160 (96·5%) |
| Asian | 0·1466 | –0·1109 | 0·4041 | 0·0783 | 1·9 | 0·061 | 28 (2·5%) | 8489 (2·3%) |
| Other | 0·2221 | –0·1033 | 0·5475 | 0·0989 | 2·2 | 0·025 | 19 (1·7%) | 4211 (1·2%) |
| Dapagliflozin 10 mg | 0·0027 | –0·0773 | 0·0827 | 0·0243 | 0·1 | 0·91 | 544 (48·6%) | 176 613 (48·7%) |
| Never (ref) |  |  |  |  |  |  | 520 (46·4%) | 169 741 (46·8%) |
| Former | 0·0078 | –0·086 | 0·1016 | 0·0285 | 0·3 | 0·78 | 345 (30·8%) | 114 832 (31·6%) |
| Current | 0·0954 | –0·0088 | 0·1997 | 0·0317 | 3 | 0·0026 | 255 (22·8%) | 78 287 (21·6%) |
| Yes | 0·0115 | –0·0736 | 0·0966 | 0·0259 | 0·4 | 0·66 | 443 (39·6%) | 144 707 (39·9%) |
| ≥130 | –0·0121 | –0·1167 | 0·0925 | 0·0318 | –0·4 | 0·7 | 271 (24·2%) | 91 141 (25·1%) |
| ≥80 | 0·0164 | –0·0838 | 0·1166 | 0·0305 | 0·5 | 0·59 | 311 (27·8%) | 103 244 (28·5%) |
| ≥30 | 0·0895 | 0 | 0·1791 | 0·0272 | 3·3 | 0·001 | 330 (29·5%) | 106 047 (29·2%) |
| ≥50 (ref) |  |  |  |  |  |  | 243 (21·7%) | 60 447 (16·7%) |
| 30–49 | –0·0315 | –0·1296 | 0·0666 | 0·0298 | –1·1 | 0·29 | 817 (72·9%) | 283 965 (78·3%) |
| <30 | –0·0732 | –0·2679 | 0·1216 | 0·0592 | –1·2 | 0·22 | 60 (5·4%) | 18 448 (5·1%) |
| <60 | –0·0201 | –0·1653 | 0·1251 | 0·0441 | –0·5 | 0·65 | 98 (8·8%) | 34 090 (9·4%) |
| STEMI | –0·0429 | –0·134 | 0·0481 | 0·0277 | –1·6 | 0·12 | 824 (73·6%) | 268 746 (74·1%) |
| Yes | –0·0358 | –0·0872 | 0·0155 | 0·0156 | –2·3 | 0·022 | 69 (6·2%) | 16 272 (4·5%) |
| >12 | 0·0321 | –0·0516 | 0·1159 | 0·0255 | 1·3 | 0·21 | 473 (42·2%) | 160 238 (44·2%) |
| No disease (ref) |  |  |  |  |  |  | 729 (65·1%) | 181 956 (50·1%) |
| No symptoms | 0·0481 | 0·0222 | 0·0739 | 0·0078 | 6·1 | 9·2e-10 | 484 (43·2%) | 107 234 (29·6%) |
| Mild symptoms | 0·0329 | 0·0032 | 0·0626 | 0·009 | 3·6 | 0·00027 | 293 (26·2%) | 66 530 (18·3%) |
| Marked/Severe | 0·0603 | –0·0162 | 0·1368 | 0·0232 | 2·6 | 0·0095 | 43 (3·8%) | 7140 (2·0%) |
| Asymptomatic (ref) |  |  |  |  |  |  | 991 (88·5%) | 293 598 (80·9%) |
| Strenuous exertion | –0·0148 | –0·0481 | 0·0186 | 0·0101 | –1·5 | 0·15 | 247 (22·1%) | 52 418 (14·4%) |
| Moderate exertion | –0·0268 | –0·0892 | 0·0356 | 0·019 | –1·4 | 0·16 | 60 (5·4%) | 14 309 (3·9%) |
| Mild exertion/At rest | –0·0243 | –0·1632 | 0·1146 | 0·0422 | –0·6 | 0·57 | 16 (1·4%) | 2535 (0·7%) |
| ≤30 (ref) |  |  |  |  |  |  | 1108 (98·9%) | 30 870 (8·5%) |
| 31–60 | 0·0001 | –0·0235 | 0·0236 | 0·0072 | 0 | 0·99 | 1088 (97·1%) | 30 648 (8·4%) |
| 61–90 | 0·0146 | –0·0093 | 0·0385 | 0·0073 | 2 | 0·045 | 1074 (95·9%) | 29 971 (8·3%) |
| 91–120 | 0·0436 | 0·0191 | 0·068 | 0·0074 | 5·9 | 4·6e-09 | 1045 (93·3%) | 27 965 (7·7%) |
| 121–150 | 0·0532 | 0·0279 | 0·0785 | 0·0077 | 6·9 | 4·4e-12 | 945 (84·4%) | 25 455 (7·0%) |
| 151–200 | 0·0646 | 0·0412 | 0·0879 | 0·0071 | 9·1 | 9·4e-20 | 877 (78·3%) | 38 294 (10·6%) |
| 201–250 | 0·0618 | 0·0374 | 0·0861 | 0·0074 | 8·3 | 7e-17 | 782 (69·8%) | 34 202 (9·4%) |
| 251–300 | 0·0764 | 0·051 | 0·1018 | 0·0077 | 9·9 | 3·9e-23 | 712 (63·6%) | 31 145 (8·6%) |
| 301–350 | 0·0884 | 0·0616 | 0·1152 | 0·0082 | 10·8 | 2·1e-27 | 646 (57·7%) | 27 295 (7·5%) |
| 351–400 | 0·0898 | 0·061 | 0·1186 | 0·0088 | 10·3 | 1·1e-24 | 537 (47·9%) | 22 262 (6·1%) |
| 401–450 | 0·1044 | 0·0735 | 0·1353 | 0·0094 | 11·1 | 9·3e-29 | 441 (39·4%) | 19 173 (5·3%) |
| 451–500 | 0·1044 | 0·0709 | 0·138 | 0·0102 | 10·2 | 1·4e-24 | 366 (32·7%) | 15 684 (4·3%) |
| >500 | 0·1098 | 0·0775 | 0·1421 | 0·0098 | 11·2 | 4·5e-29 | 294 (26·2%) | 29 896 (8·2%) |
| January (ref) |  |  |  |  |  |  | 993 (88·7%) | 37 803 (10·4%) |
| February | –0·0192 | –0·041 | 0·0025 | 0·0066 | –2·9 | 0·0036 | 1031 (92·1%) | 36 761 (10·1%) |
| March | –0·0328 | –0·0621 | –0·0035 | 0·0089 | –3·7 | 0·00023 | 1035 (92·4%) | 41 052 (11·3%) |
| April | –0·023 | –0·0519 | 0·006 | 0·0088 | –2·6 | 0·0091 | 986 (88·0%) | 31 362 (8·6%) |
| May | 0·0187 | –0·0109 | 0·0483 | 0·009 | 2·1 | 0·038 | 826 (73·8%) | 23 144 (6·4%) |
| June | 0·0274 | –0·0028 | 0·0575 | 0·0092 | 3 | 0·0028 | 701 (62·6%) | 20 971 (5·8%) |
| July | 0·022 | –0·008 | 0·052 | 0·0091 | 2·4 | 0·016 | 711 (63·5%) | 23 123 (6·4%) |
| August | 0·0011 | –0·0287 | 0·0309 | 0·009 | 0·1 | 0·9 | 754 (67·3%) | 24 792 (6·8%) |
| September | –0·0249 | –0·0543 | 0·0045 | 0·0089 | –2·8 | 0·0054 | 807 (72·1%) | 26 393 (7·3%) |
| October | –0·0174 | –0·0466 | 0·0119 | 0·0089 | –2 | 0·051 | 860 (76·8%) | 30 330 (8·4%) |
| November | –0·0289 | –0·0579 | 0·0001 | 0·0088 | –3·3 | 0·001 | 907 (81·0%) | 31 785 (8·8%) |
| December | 0·0045 | –0·0242 | 0·0332 | 0·0087 | 0·5 | 0·61 | 950 (84·8%) | 35 344 (9·7%) |
| Monday | 0·3248 | 0·3068 | 0·3428 | 0·0055 | 59·4 | 0·0 | 1120 (100·0%) | 52 005 (14·3%) |
| Tuesday | 0·0336 | 0·0157 | 0·0514 | 0·0054 | 6·2 | 6·1e-10 | 1119 (99·9%) | 52 040 (14·3%) |
| Wednesday (ref) |  |  |  |  |  |  | 1120 (100·0%) | 51 989 (14·3%) |
| Thursday | 0·0042 | –0·0137 | 0·022 | 0·0054 | 0·8 | 0·44 | 1120 (100·0%) | 51 913 (14·3%) |
| Friday | 0·0404 | 0·0225 | 0·0583 | 0·0054 | 7·4 | 1·2e-13 | 1120 (100·0%) | 51 780 (14·3%) |
| Saturday | 0·2793 | 0·2613 | 0·2972 | 0·0055 | 51·2 | 0·0 | 1119 (99·9%) | 51 522 (14·2%) |
| Sunday | 0·17 | 0·152 | 0·188 | 0·0055 | 31·1 | 2·9e-212 | 1119 (99·9%) | 51 611 (14·2%) |
| Holiday | 0·0084 | –0·0172 | 0·034 | 0·0078 | 1·1 | 0·28 | 1085 (96·9%) | 14 549 (4·0%) |
| Yes | 0·0305 | 0·0004 | 0·0605 | 0·0091 | 3·3 | 0·00086 | 980 (87·5%) | 45 912 (12·7%) |
| No AE (ref) |  |  |  |  |  |  | 1110 (99·1%) | 345 718 (95·3%) |
| Neither | 0·1044 | 0·0429 | 0·1659 | 0·0187 | 5·6 | 2·3e-08 | 99 (8·8%) | 12 349 (3·4%) |
| Discontinued only | 0·1032 | –0·0581 | 0·2645 | 0·049 | 2·1 | 0·035 | 16 (1·4%) | 540 (0·1%) |
| Hospitalized only | 0·125 | 0·0592 | 0·1908 | 0·02 | 6·3 | 4e-10 | 137 (12·2%) | 3539 (1·0%) |
| Both | 0·1921 | 0·0667 | 0·3174 | 0·0381 | 5 | 4·6e-07 | 41 (3·7%) | 714 (0·2%) |
| No AE (ref) |  |  |  |  |  |  | 1120 (100·0%) | 361 127 (99·5%) |
| Not hospitalized or discontinued | –0·0094 | –0·1378 | 0·119 | 0·039 | –0·2 | 0·81 | 60 (5·4%) | 523 (0·1%) |
| Not hospitalized but discontinued | 0·1471 | –0·3648 | 0·659 | 0·1556 | 0·9 | 0·34 | 5 (0·4%) | 32 (0·0%) |
| Hospitalized, not discontinued | 0·0283 | –0·0643 | 0·121 | 0·0282 | 1 | 0·31 | 132 (11·8%) | 1017 (0·3%) |
| Hospitalized and discontinued | 0·0524 | –0·177 | 0·2817 | 0·0697 | 0·8 | 0·45 | 28 (2·5%) | 161 (0·0%) |
| Yes | 0·0603 | 0·0081 | 0·1125 | 0·0159 | 3·8 | 0·00014 | 647 (57·8%) | 3130 (0·9%) |
| Yes | 0·003 | –0·0182 | 0·0242 | 0·0064 | 0·5 | 0·64 | 682 (60·9%) | 22 447 (6·2%) |
| Yes | 0·0805 | 0·0626 | 0·0983 | 0·0054 | 14·8 | 8·2e-50 | 1061 (94·7%) | 32 033 (8·8%) |
| 0–4 | 0·0736 | 0·0049 | 0·1424 | 0·0209 | 3·5 | 0·00042 | 113 (10·1%) | 2474 (0·7%) |
| 4–8 | –0·0802 | –0·0953 | –0·065 | 0·0046 | –17·4 | 4·3e-68 | 868 (77·5%) | 128 914 (35·5%) |
| 8–12 (ref) |  |  |  |  |  |  | 1031 (92·1%) | 189 822 (52·3%) |
| 12–16 | 0·2976 | 0·2676 | 0·3276 | 0·0091 | 32·6 | 1·7e-233 | 418 (37·3%) | 18 449 (5·1%) |
| 16–20 | 0·1534 | 0·1093 | 0·1975 | 0·0134 | 11·4 | 2·4e-30 | 261 (23·3%) | 13 401 (3·7%) |
| 20–24 | –0·0664 | –0·124 | –0·0089 | 0·0175 | –3·8 | 0·00015 | 121 (10·8%) | 9800 (2·7%) |
| Yes | 0·2405 | 0·2004 | 0·2806 | 0·0122 | 19·7 | 1e-86 | 494 (44·1%) | 5653 (1·6%) |
| Yes | 0·0262 | 0·0109 | 0·0415 | 0·0047 | 5·6 | 1·8e-08 | 1090 (97·3%) | 49 064 (13·5%) |
| No recent non-adherence (ref) |  |  |  |  |  |  | 1118 (99·8%) | 303 829 (83·7%) |
| 1 day after ≤3 missed | 0·371 | 0·342 | 0·4 | 0·0088 | 42·1 | 0·0 | 988 (88·2%) | 11090 (3·1%) |
| 2 days after ≤3 missed | 0·0958 | 0·0649 | 0·1268 | 0·0094 | 10·2 | 2e-24 | 984 (87·9%) | 9507 (2·6%) |
| 3 days after ≤3 missed | 0·0414 | 0·0089 | 0·0739 | 0·0099 | 4·2 | 2·8e-05 | 982 (87·7%) | 8465 (2·3%) |
| 4 days after ≤3 missed | 0·0207 | –0·0132 | 0·0546 | 0·0103 | 2 | 0·045 | 979 (87·4%) | 7677 (2·1%) |
| 5 days after ≤3 missed | –0·002 | –0·0373 | 0·0332 | 0·0107 | –0·2 | 0·85 | 975 (87·1%) | 7041 (1·9%) |
| 6 days after ≤3 missed | 0·0375 | 0·0009 | 0·074 | 0·0111 | 3·4 | 0·00075 | 973 (86·9%) | 6487 (1·8%) |
| 7 days after ≤3 missed | 0·0475 | 0·0093 | 0·0857 | 0·0116 | 4·1 | 4·2e-05 | 967 (86·3%) | 5893 (1·6%) |
| 1 day after >3 missed | 0·6323 | 0·53 | 0·7345 | 0·0311 | 20·3 | 5·1e-92 | 326 (29·1%) | 871 (0·2%) |
| 2 days after >3 missed | 0·3349 | 0·206 | 0·4638 | 0·0392 | 8·5 | 1·3e-17 | 295 (26·3%) | 506 (0·1%) |
| 3 days after >3 missed | 0·0638 | –0·0829 | 0·2106 | 0·0446 | 1·4 | 0·15 | 271 (24·2%) | 388 (0·1%) |
| 4 days after >3 missed | 0·1225 | –0·037 | 0·282 | 0·0485 | 2·5 | 0·011 | 248 (22·1%) | 328 (0·1%) |
| 5 days after >3 missed | 0·0129 | –0·158 | 0·1839 | 0·0519 | 0·2 | 0·8 | 227 (20·3%) | 285 (0·1%) |
| 6 days after >3 missed | –0·0285 | –0·2081 | 0·1511 | 0·0546 | –0·5 | 0·6 | 209 (18·7%) | 258 (0·1%) |
| 7 days after >3 missed | 0·0712 | –0·1169 | 0·2593 | 0·0572 | 1·2 | 0·21 | 198 (17·7%) | 235 (0·1%) |
| Yes | –0·0558 | –0·1491 | 0·0375 | 0·0283 | –2 | 0·049 | 277 (24·7%) | 88 626 (24·4%) |
| Yes | 0·0156 | –0·0123 | 0·0436 | 0·0085 | 1·8 | 0·066 | 1019 (91·0%) | 266 622 (73·5%) |
| No (ref) |  |  |  |  |  |  | 851 (76·0%) | 236 420 (65·2%) |
| Moderate (3L) | –0·0854 | –0·1214 | –0·0494 | 0·0109 | –7·8 | 5·9e-15 | 155 (13·8%) | 38 228 (10·5%) |
| Extreme (3L) | –0·0836 | –0·1901 | 0·0229 | 0·0324 | –2·6 | 0·0098 | 12 (1·1%) | 3489 (1·0%) |
| Slight (5L) | –0·0044 | –0·0306 | 0·0219 | 0·008 | –0·5 | 0·58 | 356 (31·8%) | 63 498 (17·5%) |
| Moderate (5L) | –0·0601 | –0·1046 | –0·0157 | 0·0135 | –4·5 | 8·5e-06 | 125 (11·2%) | 16 246 (4·5%) |
| Severe/Extreme (5L) | 0·0307 | –0·0553 | 0·1168 | 0·0261 | 1·2 | 0·24 | 28 (2·5%) | 4979 (1·4%) |

Results of the linear mixed effects regression model fit on the subset of the Box-Cox-transformed dose consistency (the absolute value of adherence time differences on consecutive days) including only patients who provided responses to EQ-5D Anxiety/Depression question. Variables with multiple values are dichotomized and reference values are indicated by (ref). Error bars represent 99·95% CIs (see corresponding Supplementary Figure 6).

AE=adverse event; BMI=body mass index; CCS=Canadian Cardiovascular Society; CI=confidence interval; eGFR=estimated glomerular filtration rate; LVEF=left ventricular ejection fraction; NYHA=New York Heart Association; SE=standard error; STEMI=ST-elevation myocardial infarction.

### Supplementary Table 9. Results of adherence model for patients with low initial dose consistency

| Feature | Value | Odds ratio | Estimate | 0·05% CI | 99·95% CI | SE | t value | Pr(>\|t\|) | No. patients | No. observations |
| --- | --- | --- | --- | --- | --- | --- | --- | --- | --- | --- |
| Country | **Sweden** | 1·145 | 0·1356 | –0·2769 | 0·5481 | 0·1254 | 1·1 | 0·28 | 299 (25·4%) | 109 867 (29·7%) |
| Sex | **Female** | 1·428 | 0·3563 | 0·0097 | 0·7029 | 0·1053 | 3·4 | 0·00072 | 262 (22·2%) | 81 629 (22·1%) |
| Age | **<40** | 0·825 | –0·1918 | –1·6572 | 1·2735 | 0·4453 | –0·4 | 0·67 | 11 (0·9%) | 3121 (0·8%) |
|  | **40–50** | 0·973 | –0·0273 | –0·6784 | 0·6238 | 0·1979 | –0·1 | 0·89 | 68 (5·8%) | 18 582 (5·0%) |
|  | **50–60** | 0·977 | –0·0229 | –0·4075 | 0·3616 | 0·1169 | –0·2 | 0·84 | 256 (21·7%) | 75 043 (20·3%) |
|  | **60–70 (ref)** |  |  |  |  |  |  |  | 425 (36·1%) | 137 521 (37·2%) |
|  | **70–80** | 0·924 | –0·0787 | –0·4413 | 0·2839 | 0·1102 | –0·7 | 0·48 | 344 (29·2%) | 112 879 (30·5%) |
|  | **>80** | 0·88 | –0·1283 | –0·7743 | 0·5177 | 0·1963 | –0·7 | 0·51 | 74 (6·3%) | 22 524 (6·1%) |
| Race | **White (ref)** |  |  |  |  |  |  |  | 1139 (96·7%) | 359 014 (97·1%) |
|  | **Asian** | 1·003 | 0·0031 | –1·0169 | 1·0231 | 0·31 | 0 | 0·99 | 24 (2·0%) | 6451 (1·7%) |
|  | **Other** | 0·533 | –0·6287 | –1·9095 | 0·6521 | 0·3892 | –1·6 | 0·11 | 15 (1·3%) | 4205 (1·1%) |
| Arm | **Dapagliflozin 10 mg** | 0·952 | –0·0496 | –0·3299 | 0·2307 | 0·0852 | –0·6 | 0·56 | 590 (50·1%) | 183 618 (49·7%) |
| Smoking status | **Never (ref)** |  |  |  |  |  |  |  | 567 (48·1%) | 180 290 (48·8%) |
|  | **Former** | 0·964 | –0·0366 | –0·3598 | 0·2866 | 0·0982 | –0·4 | 0·71 | 365 (31·0%) | 117 844 (31·9%) |
|  | **Current** | 0·716 | –0·3346 | –0·7193 | 0·0501 | 0·1169 | –2·9 | 0·0042 | 246 (20·9%) | 71 536 (19·4%) |
| Hypertension history | **Yes** | 0·873 | –0·136 | –0·4361 | 0·1641 | 0·0912 | –1·5 | 0·14 | 466 (39·6%) | 147 860 (40·0%) |
| Systolic pressure | **≥130** | 0·995 | –0·0051 | –0·3748 | 0·3645 | 0·1123 | 0 | 0·96 | 272 (23·1%) | 87 708 (23·7%) |
| Diastolic pressure | **≥80** | 0·897 | –0·1081 | –0·4845 | 0·2682 | 0·1144 | –0·9 | 0·34 | 258 (21·9%) | 82 197 (22·2%) |
| BMI | **≥30** | 1·074 | 0·0711 | –0·2477 | 0·3899 | 0·0969 | 0·7 | 0·46 | 339 (28·8%) | 103 860 (28·1%) |
| LVEF | **≥50 (ref)** |  |  |  |  |  |  |  | 237 (20·1%) | 58 156 (15·7%) |
|  | **30–49** | 0·887 | –0·1195 | –0·4815 | 0·2426 | 0·11 | –1·1 | 0·28 | 858 (72·8%) | 288 627 (78·1%) |
|  | **<30** | 0·894 | –0·1125 | –0·7496 | 0·5246 | 0·1936 | –0·6 | 0·56 | 83 (7·0%) | 22 887 (6·2%) |
| eGFR | **<60** | 0·987 | –0·0129 | –0·4636 | 0·4377 | 0·137 | –0·1 | 0·92 | 147 (12·5%) | 45 213 (12·2%) |
| STEMI | **STEMI** | 1·203 | 0·185 | –0·1366 | 0·5067 | 0·0978 | 1·9 | 0·058 | 848 (72·0%) | 267 331 (72·3%) |
| Revascularization | **Yes** | 0·987 | –0·013 | –0·3006 | 0·2747 | 0·0874 | –0·1 | 0·88 | 96 (8·1%) | 20 461 (5·5%) |
| # medications | **> 12** | 0·937 | –0·0649 | –0·3626 | 0·2329 | 0·0905 | –0·7 | 0·47 | 567 (48·1%) | 178 329 (48·2%) |
| NYHA | **No disease (ref)** |  |  |  |  |  |  |  | 720 (61·1%) | 170 384 (46·1%) |
|  | **No symptoms** | 1·11 | 0·1044 | –0·0765 | 0·2853 | 0·055 | 1·9 | 0·058 | 561 (47·6%) | 123 216 (33·3%) |
|  | **Mild symptoms** | 1·073 | 0·0704 | –0·1361 | 0·2769 | 0·0628 | 1·1 | 0·26 | 300 (25·5%) | 67 932 (18·4%) |
|  | **Marked/Severe** | 1·488 | 0·3973 | –0·0048 | 0·7994 | 0·1222 | 3·3 | 0·0011 | 55 (4·7%) | 8138 (2·2%) |
| CCS Angina grade | **Asymptomatic (ref)** |  |  |  |  |  |  |  | 1012 (85·9%) | 285 897 (77·3%) |
|  | **Strenuous exertion** | 1·227 | 0·2043 | 0·0107 | 0·3978 | 0·0588 | 3·5 | 0·00052 | 321 (27·2%) | 66 274 (17·9%) |
|  | **Moderate exertion** | 0·66 | –0·4161 | –0·7894 | –0·0428 | 0·1134 | –3·7 | 0·00024 | 64 (5·4%) | 12 837 (3·5%) |
|  | **Mild exertion/At rest** | 0·955 | –0·0461 | –0·7563 | 0·6642 | 0·2158 | –0·2 | 0·83 | 23 (2·0%) | 4662 (1·3%) |
| Days from start | **≤30 (ref)** |  |  |  |  |  |  |  | 1178 (100·0%) | 36 038 (9·7%) |
|  | **31–60** | 0·962 | –0·0387 | –0·2009 | 0·1236 | 0·0493 | –0·8 | 0·43 | 1164 (98·8%) | 34 294 (9·3%) |
|  | **61–90** | 0·818 | –0·2004 | –0·3637 | –0·0371 | 0·0496 | –4 | 5·4e-05 | 1124 (95·4%) | 33 014 (8·9%) |
|  | **91–120** | 0·708 | –0·3454 | –0·5122 | –0·1787 | 0·0507 | –6·8 | 9·3e-12 | 1081 (91·8%) | 30 442 (8·2%) |
|  | **121–150** | 0·653 | –0·4255 | –0·5958 | –0·2553 | 0·0517 | –8·2 | 2e-16 | 973 (82·6%) | 27 727 (7·5%) |
|  | **151–200** | 0·647 | –0·4358 | –0·5953 | –0·2764 | 0·0485 | –9 | 2·4e-19 | 897 (76·1%) | 41 360 (11·2%) |
|  | **201–250** | 0·675 | –0·3936 | –0·562 | –0·2253 | 0·0512 | –7·7 | 1·4e-14 | 783 (66·5%) | 35 389 (9·6%) |
|  | **251–300** | 0·641 | –0·4444 | –0·621 | –0·2677 | 0·0537 | –8·3 | 1·3e-16 | 675 (57·3%) | 31 245 (8·5%) |
|  | **301–350** | 0·667 | –0·4052 | –0·5946 | –0·2159 | 0·0575 | –7 | 1·9e-12 | 599 (50·8%) | 26 509 (7·2%) |
|  | **351–400** | 0·807 | –0·2141 | –0·4258 | –0·0023 | 0·0644 | –3·3 | 0·00088 | 474 (40·2%) | 20 035 (5·4%) |
|  | **401–450** | 0·717 | –0·3332 | –0·5627 | –0·1038 | 0·0697 | –4·8 | 1·8e-06 | 363 (30·8%) | 16 471 (4·5%) |
|  | **451–500** | 0·578 | –0·5482 | –0·7881 | –0·3084 | 0·0729 | –7·5 | 5·5e-14 | 298 (25·3%) | 13 164 (3·6%) |
|  | **>500** | 0·491 | –0·7107 | –0·9441 | –0·4773 | 0·0709 | –10 | 1·2e-23 | 226 (19·2%) | 23 982 (6·5%) |
| Month | **January (ref)** |  |  |  |  |  |  |  | 1020 (86·6%) | 38 648 (10·5%) |
|  | **February** | 0·874 | –0·1348 | –0·2858 | 0·0162 | 0·0459 | –2·9 | 0·0033 | 1076 (91·3%) | 37 791 (10·2%) |
|  | **March** | 1·286 | 0·2516 | 0·0407 | 0·4625 | 0·0641 | 3·9 | 8·6e-05 | 1081 (91·8%) | 42 662 (11·5%) |
|  | **April** | 1·012 | 0·0117 | –0·1936 | 0·217 | 0·0624 | 0·2 | 0·85 | 1007 (85·5%) | 32 541 (8·8%) |
|  | **May** | 0·829 | –0·1872 | –0·394 | 0·0195 | 0·0628 | –3 | 0·0029 | 852 (72·3%) | 23 537 (6·4%) |
|  | **June** | 0·783 | –0·2446 | –0·4567 | –0·0326 | 0·0644 | –3·8 | 0·00015 | 675 (57·3%) | 20 406 (5·5%) |
|  | **July** | 1·003 | 0·003 | –0·2154 | 0·2214 | 0·0664 | 0 | 0·96 | 698 (59·3%) | 23 031 (6·2%) |
|  | **August** | 0·904 | –0·1009 | –0·3146 | 0·1128 | 0·0649 | –1·6 | 0·12 | 759 (64·4%) | 24 829 (6·7%) |
|  | **September** | 0·989 | –0·0115 | –0·2269 | 0·2038 | 0·0654 | –0·2 | 0·86 | 822 (69·8%) | 26 927 (7·3%) |
|  | **October** | 0·997 | –0·0027 | –0·2166 | 0·2111 | 0·065 | 0 | 0·97 | 864 (73·3%) | 30 733 (8·3%) |
|  | **November** | 0·94 | –0·0617 | –0·2738 | 0·1504 | 0·0645 | –1 | 0·34 | 921 (78·2%) | 32 313 (8·7%) |
|  | **December** | 0·979 | –0·0216 | –0·2321 | 0·1889 | 0·064 | –0·3 | 0·74 | 974 (82·7%) | 36 252 (9·8%) |
| Days of the week | **Monday** | 0·965 | –0·036 | –0·1573 | 0·0854 | 0·0369 | –1 | 0·33 | 1178 (100·0%) | 52 821 (14·3%) |
|  | **Tuesday** | 1·053 | 0·0513 | –0·0706 | 0·1732 | 0·037 | 1·4 | 0·17 | 1178 (100·0%) | 52 873 (14·3%) |
|  | **Wednesday (ref)** |  |  |  |  |  |  |  | 1178 (100·0%) | 52 824 (14·3%) |
|  | **Thursday** | 0·965 | –0·0352 | –0·1554 | 0·0851 | 0·0366 | –1 | 0·34 | 1178 (100·0%) | 52 830 (14·3%) |
|  | **Friday** | 0·925 | –0·0776 | –0·1972 | 0·0421 | 0·0364 | –2·1 | 0·033 | 1178 (100·0%) | 52 837 (14·3%) |
|  | **Saturday** | 0·871 | –0·138 | –0·2563 | –0·0198 | 0·0359 | –3·8 | 0·00012 | 1178 (100·0%) | 52 746 (14·3%) |
|  | **Sunday** | 0·857 | –0·1548 | –0·2732 | –0·0365 | 0·036 | –4·3 | 1·7e-05 | 1178 (100·0%) | 52 739 (14·3%) |
| Holiday | **Holiday** | 0·966 | –0·0345 | –0·2005 | 0·1316 | 0·0505 | –0·7 | 0·49 | 1146 (97·3%) | 14 434 (3·9%) |
| Study Closeout | **Yes** | 0·458 | –0·7807 | –0·9847 | –0·5768 | 0·062 | –12·6 | 2·1e-36 | 1022 (86·8%) | 52 694 (14·3%) |
| Hospitalized or discontinued | **No AE (ref)** |  |  |  |  |  |  |  | 1169 (99·2%) | 347 326 (94·0%) |
|  | **Neither** | 0·571 | –0·5598 | –0·8806 | –0·239 | 0·0975 | –5·7 | 9·4e-09 | 136 (11·5%) | 15 896 (4·3%) |
|  | **Discontinued only** | 0·029 | –3·5353 | –4·2809 | –2·7897 | 0·2266 | –15·6 | 7e-55 | 20 (1·7%) | 613 (0·2%) |
|  | **Hospitalized only** | 0·129 | –2·0468 | –2·3434 | –1·7501 | 0·0901 | –22·7 | 4e-114 | 164 (13·9%) | 4223 (1·1%) |
|  | **Both** | 0·059 | –2·8274 | –3·2101 | –2·4448 | 0·1163 | –24·3 | 1·4e-130 | 59 (5·0%) | 1612 (0·4%) |
| 7 days after AE | **No AE (ref)** |  |  |  |  |  |  |  | 1178 (100·0%) | 367 518 (99·4%) |
|  | **Not hospitalized or discontinued** | 1·13 | 0·1219 | –0·7034 | 0·9471 | 0·2508 | 0·5 | 0·63 | 85 (7·2%) | 657 (0·2%) |
|  | **Not hospitalized but discontinued** | 0·137 | –1·9868 | –3·3954 | –0·5783 | 0·4281 | –4·6 | 3·5e-06 | 4 (0·3%) | 40 (0·0%) |
|  | **Hospitalized, not discontinued** | 0·776 | –0·2534 | –0·7506 | 0·2439 | 0·1511 | –1·7 | 0·094 | 136 (11·5%) | 1196 (0·3%) |
|  | **Hospitalized and discontinued** | 0·218 | –1·5224 | –2·1186 | –0·9263 | 0·1812 | –8·4 | 4·4e-17 | 34 (2·9%) | 259 (0·1%) |
| Mobile application use | **Yes** | 1·118 | 0·1112 | –0·4001 | 0·6226 | 0·1554 | 0·7 | 0·47 | 328 (27·8%) | 1801 (0·5%) |
| 7 days after mobile application use | **Yes** | 1·234 | 0·2101 | –0·0046 | 0·4247 | 0·0652 | 3·2 | 0·0013 | 328 (27·8%) | 12 496 (3·4%) |
| Off-schedule use in last 7 days | **Yes** | 0·421 | –0·8651 | –0·9607 | –0·7695 | 0·0291 | –29·8 | 9·1e-195 | 1115 (94·7%) | 26 057 (7·0%) |
| Average time in last 7 days | **0–4** | 0·913 | –0·0913 | –0·6161 | 0·4335 | 0·1595 | –0·6 | 0·57 | 57 (4·8%) | 2028 (0·5%) |
|  | **4–8** | 1·111 | 0·1054 | –0·0011 | 0·2119 | 0·0324 | 3·3 | 0·0011 | 835 (70·9%) | 130 225 (35·2%) |
|  | **8–12 (ref)** |  |  |  |  |  |  |  | 1032 (87·6%) | 202 436 (54·8%) |
|  | **12–16** | 0·849 | –0·1637 | –0·4308 | 0·1034 | 0·0812 | –2 | 0·044 | 215 (18·3%) | 7868 (2·1%) |
|  | **16–20** | 0·723 | –0·3246 | –0·6389 | –0·0104 | 0·0955 | –3·4 | 0·00068 | 169 (14·3%) | 14 908 (4·0%) |
|  | **20–24** | 0·385 | –0·9543 | –1·3248 | –0·5838 | 0·1126 | –8·5 | 2·3e-17 | 87 (7·4%) | 12 205 (3·3%) |
| Average time variance in last 7 days | **<15 min** | 2·293 | 0·8297 | 0·6609 | 0·9985 | 0·0513 | 16·2 | 8·1e-59 | 1009 (85·7%) | 58 744 (15·9%) |
|  | **15–30 min** | 1·974 | 0·6801 | 0·5334 | 0·8269 | 0·0446 | 15·2 | 1·7e-52 | 1171 (99·4%) | 100 195 (27·1%) |
|  | **30–60 min** | 1·605 | 0·4733 | 0·3327 | 0·614 | 0·0427 | 11·1 | 1·7e-28 | 1162 (98·6%) | 143 156 (38·7%) |
|  | **1–2 h** | 1·636 | 0·492 | 0·3404 | 0·6437 | 0·0461 | 10·7 | 1·3e-26 | 1102 (93·5%) | 55 421 (15·0%) |
|  | **>2 h (ref)** |  |  |  |  |  |  |  | 736 (62·5%) | 12 154 (3·3%) |
| 7 days after data gap | **Yes** | 0·669 | –0·4015 | –0·625 | –0·1779 | 0·0679 | –5·9 | 3·4e-09 | 402 (34·1%) | 4581 (1·2%) |
| 7 days after a holiday | **Yes** | 1·054 | 0·0523 | –0·0514 | 0·1559 | 0·0315 | 1·7 | 0·097 | 1145 (97·2%) | 47 703 (12·9%) |
| Lost >5% weight | **Yes** | 0·893 | –0·1135 | –0·4363 | 0·2092 | 0·0981 | –1·2 | 0·25 | 308 (26·1%) | 100 039 (27·1%) |
| COVID-19 restrictions | **Yes** | 0·875 | –0·1336 | –0·3349 | 0·0678 | 0·0612 | –2·2 | 0·029 | 1061 (90·1%) | 262 121 (70·9%) |
| Intercept |  | 80·388 | 4·3869 | 3·8046 | 4·9691 | 0·1769 | 24·8 | 1·1e-135 | 1178 (100%) | 369 670 (100%) |

Results of the logistic mixed effects regression model fit on low-initial dose consistency subset of patients of the adherence dataset. Patients included in this dataset had below-median average dose consistency between days 7 and 35 after randomization. Only patients who had at least 10 days of data during that period were included in this analysis. The coefficients are represented by the blue colour in corresponding Supplementary Figure 7. Variables with multiple values are dichotomized and reference values are indicated by (ref). Error bars represent 99·95% CIs.

AE=adverse event; BMI=body mass index; CCS=Canadian Cardiovascular Society; CI=confidence interval; eGFR=estimated glomerular filtration rate; LVEF=left ventricular ejection fraction; NYHA=New York Heart Association; SE=standard error; STEMI=ST-elevation myocardial infarction.

### Supplementary Table 10. Results of adherence model for patients with high initial dose consistency

| Group | Feature | Value | Odds ratio | Estimate | 0·05% CI | 99·95% CI | SE | t value | Pr(> \|t\|) | No. patients | No. observations |
| --- | --- | --- | --- | --- | --- | --- | --- | --- | --- | --- | --- |
| Patient characteristics | **Country** | **Sweden** | 1·15 | 0·1399 | –0·3163 | 0·5961 | 0·1386 | 1 | 0·31 | 221 (18·8%) | 76 565 (21·7%) |
|  | **Sex** | **Female** | 0·854 | –0·1575 | –0·5417 | 0·2268 | 0·1168 | –1·3 | 0·18 | 203 (17·2%) | 60 134 (17·0%) |
|  | **Age** | **<40** | 0·564 | –0·5736 | –1·4072 | 0·26 | 0·2533 | –2·3 | 0·024 | 39 (3·3%) | 9494 (2·7%) |
|  |  | **40–50** | 0·666 | –0·4069 | –0·8803 | 0·0665 | 0·1439 | –2·8 | 0·0047 | 152 (12·9%) | 43 499 (12·3%) |
|  |  | **50–60** | 0·876 | –0·1327 | –0·4826 | 0·2173 | 0·1063 | –1·2 | 0·21 | 413 (35·1%) | 116 965 (33·2%) |
|  |  | **60–70 (ref)** |  |  |  |  |  |  |  | 363 (30·8%) | 116 028 (32·9%) |
|  |  | **70–80** | 0·778 | –0·2517 | –0·7047 | 0·2014 | 0·1377 | –1·8 | 0·068 | 186 (15·8%) | 59 362 (16·8%) |
|  |  | **>80** | 0·6 | –0·5104 | –1·5529 | 0·5322 | 0·3168 | –1·6 | 0·11 | 24 (2·0%) | 7480 (2·1%) |
|  | **Race** | **White (ref)** |  |  |  |  |  |  |  | 1107 (94·1%) | 334 902 (94·9%) |
|  |  | **Asian** | 0·827 | –0·1905 | –0·9111 | 0·53 | 0·219 | –0·9 | 0·38 | 51 (4·3%) | 13 604 (3·9%) |
|  |  | **Other** | 0·554 | –0·591 | –1·8082 | 0·6263 | 0·3699 | –1·6 | 0·11 | 19 (1·6%) | 4322 (1·2%) |
|  | **Arm** | **Dapagliflozin 10 mg** | 0·995 | –0·0047 | –0·2894 | 0·28 | 0·0865 | –0·1 | 0·96 | 574 (48·8%) | 175 178 (49·6%) |
|  | **Smoking status** | **Never (ref)** |  |  |  |  |  |  |  | 452 (38·4%) | 142 092 (40·3%) |
|  |  | **Former** | 1·002 | 0·0017 | –0·3582 | 0·3617 | 0·1094 | 0 | 0·99 | 303 (25·7%) | 91 414 (25·9%) |
|  |  | **Current** | 0·816 | –0·2034 | –0·5436 | 0·1368 | 0·1034 | –2 | 0·049 | 422 (35·9%) | 119 322 (33·8%) |
|  | **Hypertension history** | **Yes** | 1·013 | 0·0131 | –0·2981 | 0·3243 | 0·0946 | 0·1 | 0·89 | 420 (35·7%) | 123 111 (34·9%) |
| Health status | **Systolic pressure** | **≥130** | 0·989 | –0·0108 | –0·3999 | 0·3784 | 0·1183 | –0·1 | 0·93 | 259 (22·0%) | 79 184 (22·4%) |
|  | **Diastolic pressure** | **≥80** | 0·932 | –0·0703 | –0·4341 | 0·2934 | 0·1105 | –0·6 | 0·52 | 321 (27·3%) | 97 539 (27·6%) |
|  | **BMI** | **≥30** | 1·018 | 0·0183 | –0·2845 | 0·3211 | 0·092 | 0·2 | 0·84 | 414 (35·2%) | 121 317 (34·4%) |
|  | **LVEF** | **≥50 (ref)** |  |  |  |  |  |  |  | 255 (21·7%) | 63 851 (18·1%) |
|  |  | **30–49** | 1·214 | 0·1937 | –0·1641 | 0·5514 | 0·1087 | 1·8 | 0·075 | 847 (72·0%) | 268 739 (76·2%) |
|  |  | **<30** | 1·174 | 0·1603 | –0·5124 | 0·8331 | 0·2044 | 0·8 | 0·43 | 75 (6·4%) | 20 238 (5·7%) |
|  | **eGFR** | **<60** | 0·937 | –0·0649 | –0·6156 | 0·4857 | 0·1673 | –0·4 | 0·7 | 88 (7·5%) | 28 898 (8·2%) |
|  | **STEMI** | **STEMI** | 1·148 | 0·1379 | –0·2091 | 0·485 | 0·1055 | 1·3 | 0·19 | 888 (75·4%) | 266 062 (75·4%) |
|  | **Revascularization** | **Yes** | 1·164 | 0·1517 | –0·0794 | 0·3828 | 0·0702 | 2·2 | 0·031 | 71 (6·0%) | 17 599 (5·0%) |
|  | **# medications** | **>12** | 0·991 | –0·0088 | –0·3107 | 0·2932 | 0·0918 | –0·1 | 0·92 | 575 (48·9%) | 178 919 (50·7%) |
|  | **NYHA** | **No disease (ref)** |  |  |  |  |  |  |  | 671 (57·0%) | 159 251 (45·1%) |
|  |  | **No symptoms** | 1·035 | 0·0341 | –0·0899 | 0·1581 | 0·0377 | 0·9 | 0·37 | 564 (47·9%) | 122 576 (34·7%) |
|  |  | **Mild symptoms** | 1·156 | 0·1447 | –0·0108 | 0·3003 | 0·0473 | 3·1 | 0·0022 | 295 (25·1%) | 59 924 (17·0%) |
|  |  | **Marked/Severe** | 1·659 | 0·5059 | 0·2171 | 0·7948 | 0·0878 | 5·8 | 8·2e-09 | 67 (5·7%) | 11 077 (3·1%) |
|  | **CCS Angina grade** | **Asymptomatic (ref)** |  |  |  |  |  |  |  | 977 (83·0%) | 264 317 (74·9%) |
|  |  | **Strenuous exertion** | 0·847 | –0·1657 | –0·3036 | –0·0279 | 0·0419 | –4 | 7·6e-05 | 327 (27·8%) | 68 145 (19·3%) |
|  |  | **Moderate exertion** | 1·003 | 0·0028 | –0·2375 | 0·2431 | 0·073 | 0 | 0·97 | 75 (6·4%) | 16 556 (4·7%) |
|  |  | **Mild exertion/At rest** | 0·981 | –0·0192 | –0·6302 | 0·5919 | 0·1857 | –0·1 | 0·92 | 24 (2·0%) | 3810 (1·1%) |
| Calendar features | **Days from start** | **≤30 (ref)** |  |  |  |  |  |  |  | 1177 (100·0%) | 35 941 (10·2%) |
|  |  | **31–60** | 0·852 | –0·16 | –0·2654 | –0·0547 | 0·032 | –5 | 5·7e-07 | 1166 (99·1%) | 34 266 (9·7%) |
|  |  | **61–90** | 0·682 | –0·382 | –0·4878 | –0·2763 | 0·0321 | –11·9 | 1·3e-32 | 1134 (96·3%) | 32 901 (9·3%) |
|  |  | **91–120** | 0·55 | –0·5982 | –0·7066 | –0·4899 | 0·0329 | –18·2 | 1e-73 | 1074 (91·2%) | 29 784 (8·4%) |
|  |  | **121–150** | 0·46 | –0·7762 | –0·888 | –0·6645 | 0·034 | –22·9 | 1·2e-115 | 940 (79·9%) | 26 402 (7·5%) |
|  |  | **151–200** | 0·449 | –0·7998 | –0·907 | –0·6925 | 0·0326 | –24·5 | 5·7e-133 | 859 (73·0%) | 38 649 (11·0%) |
|  |  | **201–250** | 0·48 | –0·734 | –0·8493 | –0·6187 | 0·035 | –21 | 1·7e-97 | 729 (61·9%) | 32 472 (9·2%) |
|  |  | **251–300** | 0·474 | –0·7459 | –0·8682 | –0·6235 | 0·0372 | –20·1 | 1·7e-89 | 633 (53·8%) | 28 862 (8·2%) |
|  |  | **301–350** | 0·474 | –0·7461 | –0·8773 | –0·615 | 0·0399 | –18·7 | 3·4e-78 | 569 (48·3%) | 24 336 (6·9%) |
|  |  | **351–400** | 0·573 | –0·5576 | –0·7032 | –0·412 | 0·0443 | –12·6 | 2·2e-36 | 460 (39·1%) | 19 723 (5·6%) |
|  |  | **401–450** | 0·564 | –0·5727 | –0·7314 | –0·4139 | 0·0482 | –11·9 | 1·7e-32 | 371 (31·5%) | 16 640 (4·7%) |
|  |  | **451–500** | 0·483 | –0·7276 | –0·8979 | –0·5573 | 0·0518 | –14·1 | 7·1e-45 | 303 (25·7%) | 13 578 (3·8%) |
|  |  | **>500** | 0·493 | –0·707 | –0·8815 | –0·5325 | 0·053 | –13·3 | 1·6e-40 | 242 (20·6%) | 19 274 (5·5%) |
|  | **Month** | **January (ref)** |  |  |  |  |  |  |  | 991 (84·2%) | 37 478 (10·6%) |
|  |  | **February** | 0·988 | –0·0117 | –0·1078 | 0·0843 | 0·0292 | –0·4 | 0·69 | 1042 (88·5%) | 36 315 (10·3%) |
|  |  | **March** | 1·002 | 0·0019 | –0·1408 | 0·1446 | 0·0434 | 0 | 0·97 | 1055 (89·6%) | 41 099 (11·6%) |
|  |  | **April** | 0·843 | –0·1705 | –0·3106 | –0·0303 | 0·0426 | –4 | 6·3e-05 | 1019 (86·6%) | 32 467 (9·2%) |
|  |  | **May** | 0·778 | –0·2509 | –0·3945 | –0·1074 | 0·0436 | –5·8 | 8·8e-09 | 848 (72·0%) | 23 231 (6·6%) |
|  |  | **June** | 0·865 | –0·1449 | –0·2975 | 0·0076 | 0·0464 | –3·1 | 0·0018 | 670 (56·9%) | 18 728 (5·3%) |
|  |  | **July** | 0·722 | –0·3255 | –0·4738 | –0·1772 | 0·0451 | –7·2 | 5·1e-13 | 667 (56·7%) | 21 110 (6·0%) |
|  |  | **August** | 0·748 | –0·2904 | –0·4383 | –0·1426 | 0·0449 | –6·5 | 1e-10 | 711 (60·4%) | 22 892 (6·5%) |
|  |  | **September** | 0·724 | –0·3228 | –0·4694 | –0·1763 | 0·0445 | –7·2 | 4·2e-13 | 773 (65·7%) | 24 343 (6·9%) |
|  |  | **October** | 0·778 | –0·2511 | –0·3978 | –0·1045 | 0·0446 | –5·6 | 1·8e-08 | 824 (70·0%) | 28 476 (8·1%) |
|  |  | **November** | 0·712 | –0·3401 | –0·485 | –0·1952 | 0·044 | –7·7 | 1·1e-14 | 889 (75·5%) | 31 265 (8·9%) |
|  |  | **December** | 0·717 | –0·3329 | –0·4762 | –0·1896 | 0·0436 | –7·6 | 2·1e-14 | 942 (80·0%) | 35 424 (10·0%) |
|  | **Days of the week** | **Monday** | 1·014 | 0·0134 | –0·0675 | 0·0944 | 0·0246 | 0·5 | 0·58 | 1177 (100·0%) | 50 398 (14·3%) |
|  |  | **Tuesday** | 1·024 | 0·0239 | –0·0561 | 0·104 | 0·0243 | 1 | 0·33 | 1177 (100·0%) | 50 453 (14·3%) |
|  |  | **Wednesday (ref)** |  |  |  |  |  |  |  | 1177 (100·0%) | 50 424 (14·3%) |
|  |  | **Thursday** | 1·004 | 0·004 | –0·0759 | 0·0839 | 0·0243 | 0·2 | 0·87 | 1177 (100·0%) | 50 404 (14·3%) |
|  |  | **Friday** | 0·953 | –0·0482 | –0·1276 | 0·0312 | 0·0241 | –2 | 0·046 | 1177 (100·0%) | 50 468 (14·3%) |
|  |  | **Saturday** | 0·822 | –0·1955 | –0·2733 | –0·1177 | 0·0236 | –8·3 | 1·3e-16 | 1177 (100·0%) | 50 347 (14·3%) |
|  |  | **Sunday** | 0·854 | –0·158 | –0·2365 | –0·0795 | 0·0239 | –6·6 | 3·6e-11 | 1177 (100·0%) | 50 334 (14·3%) |
|  | **Holiday** | **Holiday** | 0·892 | –0·1145 | –0·2241 | –0·0048 | 0·0333 | –3·4 | 0·00059 | 1150 (97·7%) | 13 850 (3·9%) |
|  | **Study Closeout** | **Yes** | 0·762 | –0·2713 | –0·4108 | –0·1318 | 0·0424 | –6·4 | 1·6e-10 | 1000 (85·0%) | 53 281 (15·1%) |
| Adverse events | **Hospitalized or discontinued** | **No AE (ref)** |  |  |  |  |  |  |  | 1170 (99·4%) | 336 332 (95·3%) |
|  |  | **Neither** | 0·692 | –0·3682 | –0·6015 | –0·1349 | 0·0709 | –5·2 | 2·1e-07 | 143 (12·1%) | 11 747 (3·3%) |
|  |  | **Discontinued only** | 0·121 | –2·116 | –2·618 | –1·6139 | 0·1526 | –13·9 | 9·8e-44 | 24 (2·0%) | 868 (0·2%) |
|  |  | **Hospitalized only** | 0·363 | –1·0127 | –1·2698 | –0·7555 | 0·0781 | –13 | 2·1e-38 | 139 (11·8%) | 2725 (0·8%) |
|  |  | **Both** | 0·129 | –2·0484 | –2·4318 | –1·665 | 0·1165 | –17·6 | 3·5e-69 | 54 (4·6%) | 1156 (0·3%) |
|  | **7 days after AE** | **No AE (ref)** |  |  |  |  |  |  |  | 1177 (100·0%) | 350 701 (99·4%) |
|  |  | **Not hospitalized or discontinued** | 0·835 | –0·1806 | –0·6306 | 0·2695 | 0·1368 | –1·3 | 0·19 | 91 (7·7%) | 777 (0·2%) |
|  |  | **Not hospitalized but discontinued** | 0·07 | –2·6588 | –3·6381 | –1·6795 | 0·2976 | –8·9 | 4·1e-19 | 11 (0·9%) | 68 (0·0%) |
|  |  | **Hospitalized, not discontinued** | 0·691 | –0·3702 | –0·7303 | –0·01 | 0·1094 | –3·4 | 0·00072 | 125 (10·6%) | 1022 (0·3%) |
|  |  | **Hospitalized and discontinued** | 0·222 | –1·5042 | –2·0364 | –0·9721 | 0·1617 | –9·3 | 1·4e-20 | 37 (3·1%) | 260 (0·1%) |
| Behavioural features | **Mobile application use** | **Yes** | 1·547 | 0·4362 | 0·0137 | 0·8586 | 0·1284 | 3·4 | 0·00068 | 345 (29·3%) | 1551 (0·4%) |
|  | **7 days after mobile application use** | **Yes** | 1·224 | 0·2017 | 0·0475 | 0·3559 | 0·0469 | 4·3 | 1·7e-05 | 346 (29·4%) | 10819 (3·1%) |
|  | **Off-schedule use in last 7 days** | **Yes** | 0·585 | –0·5364 | –0·5965 | –0·4764 | 0·0183 | –29·4 | 8·8e-190 | 1104 (93·8%) | 42 252 (12·0%) |
|  | **Average time in last 7 days** | **0–4** | 0·803 | –0·2189 | –0·4858 | 0·048 | 0·0811 | –2·7 | 0·007 | 122 (10·4%) | 2349 (0·7%) |
|  |  | **4–8** | 1·063 | 0·0613 | –0·0163 | 0·139 | 0·0236 | 2·6 | 0·0094 | 766 (65·1%) | 78 253 (22·2%) |
|  |  | **8–12 (ref)** |  |  |  |  |  |  |  | 1067 (90·7%) | 188 012 (53·3%) |
|  |  | **12–16** | 0·782 | –0·246 | –0·3268 | –0·1652 | 0·0246 | –10 | 1·2e-23 | 731 (62·1%) | 42 808 (12·1%) |
|  |  | **16–20** | 0·746 | –0·2933 | –0·4058 | –0·1808 | 0·0342 | –8·6 | 9·5e-18 | 429 (36·4%) | 25 110 (7·1%) |
|  |  | **20–24** | 0·663 | –0·4106 | –0·5589 | –0·2623 | 0·0451 | –9·1 | 8·3e-20 | 190 (16·1%) | 16 296 (4·6%) |
|  | **Average time variance in last 7 days** | **<15 min** | 1·573 | 0·4531 | 0·2029 | 0·7033 | 0·076 | 6 | 2·5e-09 | 346 (29·4%) | 3522 (1·0%) |
|  |  | **15–30 min** | 1·581 | 0·4582 | 0·3349 | 0·5816 | 0·0375 | 12·2 | 2·2e-34 | 901 (76·6%) | 17 817 (5·0%) |
|  |  | **30–60 min** | 1·353 | 0·3026 | 0·234 | 0·3712 | 0·0208 | 14·5 | 1e-47 | 1130 (96·0%) | 84 970 (24·1%) |
|  |  | **1–2h** | 1·27 | 0·2393 | 0·1831 | 0·2955 | 0·0171 | 14 | 1·2e-44 | 1162 (98·7%) | 140 267 (39·8%) |
|  |  | **>2 h (ref)** |  |  |  |  |  |  |  | 1165 (99·0%) | 106 252 (30·1%) |
| Disruptions | **7 days after data gap** | **Yes** | 1·198 | 0·1807 | 0·0326 | 0·3288 | 0·045 | 4 | 6e-05 | 523 (44·4%) | 7147 (2·0%) |
|  | **7 days after a holiday** | **Yes** | 1·003 | 0·0031 | –0·0653 | 0·0715 | 0·0208 | 0·1 | 0·88 | 1151 (97·8%) | 44 967 (12·7%) |
| Other | **Lost >5% weight** | **Yes** | 0·918 | –0·0856 | –0·4294 | 0·2581 | 0·1045 | –0·8 | 0·41 | 262 (22·3%) | 75 437 (21·4%) |
|  | **COVID-19 restrictions** | **Yes** | 1·391 | 0·3297 | 0·187 | 0·4723 | 0·0434 | 7·6 | 2·9e-14 | 1033 (87·8%) | 247 470 (70·1%) |
|  | **Intercept** |  | 27·361 | 3·3091 | 2·729 | 3·8892 | 0·1763 | 18·8 | 1·3e-78 | 1177 (100%) | 352 828 (100%) |

Results of the logistic mixed effects regression model fit on high-initial dose consistency subset of patients of the adherence dataset. Patients included in this dataset had above-median average dose consistency between days 7 and 35 after randomization. Only patients who had at least 10 days of data during that period were included in this analysis. The coefficients are represented by the red colour in corresponding Supplementary Figure 7. Variables with multiple values are dichotomized and reference values are indicated by (ref). Error bars represent 99·95% CIs.

AE=adverse event; BMI=body mass index; CCS=Canadian Cardiovascular Society; CI=confidence interval; eGFR=estimated glomerular filtration rate; LVEF=left ventricular ejection fraction; NYHA=New York Heart Association; SE=standard error; STEMI=ST-elevation myocardial infarction.

### Supplementary Table 11. Results of Box–Cox-transformed dose consistency model for patients with low initial dose consistency

| Group | Feature | Value | Estimate | 0·05 % | 99·95 % | SE | z value | Pr(> \|z\|) | No. patients | No. observations |
| --- | --- | --- | --- | --- | --- | --- | --- | --- | --- | --- |
| Patient characteristics | **Country** | **Sweden** | 0·0076 | –0·0931 | 0·1083 | 0·0306 | 0·2 | 0·8 | 299 (25·4%) | 106109 (29·8%) |
|  | **Sex** | **Female** | –0·0409 | –0·1268 | 0·0451 | 0·0261 | –1·6 | 0·12 | 262 (22·2%) | 79 256 (22·3%) |
|  | **Age** | **<40** | –0·0964 | –0·4678 | 0·2751 | 0·1129 | –0·9 | 0·39 | 11 (0·9%) | 2982 (0·8%) |
|  |  | **40–50** | 0·0911 | –0·0721 | 0·2543 | 0·0496 | 1·8 | 0·066 | 68 (5·8%) | 17 841 (5·0%) |
|  |  | **50–60** | 0·065 | –0·0317 | 0·1616 | 0·0294 | 2·2 | 0·027 | 256 (21·7%) | 71 998 (20·2%) |
|  |  | **60–70 (ref)** |  |  |  |  |  |  | 425 (36·1%) | 132 711 (37·3%) |
|  |  | **70–80** | –0·0924 | –0·1837 | –0·0012 | 0·0277 | –3·3 | 0·00086 | 344 (29·2%) | 108 616 (30·5%) |
|  |  | **>80** | –0·1312 | –0·2924 | 0·0301 | 0·049 | –2·7 | 0·0074 | 74 (6·3%) | 21 667 (6·1%) |
|  | **Race** | **White (ref)** |  |  |  |  |  |  | 1139 (96·7%) | 345 618 (97·1%) |
|  |  | **Asian** | 0·0866 | –0·1694 | 0·3426 | 0·0778 | 1·1 | 0·27 | 24 (2·0%) | 6192 (1·7%) |
|  |  | **Other** | 0·1069 | –0·219 | 0·4328 | 0·099 | 1·1 | 0·28 | 15 (1·3%) | 4005 (1·1%) |
|  | **Arm** | **Dapagliflozin 10 mg** | –0·0243 | –0·0949 | 0·0463 | 0·0215 | –1·1 | 0·26 | 590 (50·1%) | 176579 (49· 6%) |
|  | **Smoking status** | **Never (ref)** |  |  |  |  |  |  | 567 (48·1%) | 174 042 (48·9%) |
|  |  | **Former** | 0·0111 | –0·0702 | 0·0924 | 0·0247 | 0·4 | 0·65 | 365 (31·0%) | 113 410 (31·9%) |
|  |  | **Current** | –0·0208 | –0·1178 | 0·0763 | 0·0295 | –0·7 | 0·48 | 246 (20·9%) | 68 363 (19·2%) |
|  | **Hypertension history** | **Yes** | 0·0191 | –0·0564 | 0·0947 | 0·023 | 0·8 | 0·41 | 466 (39·6%) | 142 291 (40·0%) |
| Health status | **Systolic pressure** | **≥130** | 0·023 | –0·0703 | 0·1162 | 0·0283 | 0·8 | 0·42 | 272 (23·1%) | 84 469 (23·7%) |
|  | **Diastolic pressure** | **≥80** | –0·0629 | –0·157 | 0·0312 | 0·0286 | –2·2 | 0·028 | 258 (21·9%) | 78 770 (22·1%) |
|  | **BMI** | **≥30** | 0·0425 | –0·0375 | 0·1225 | 0·0243 | 1·7 | 0·08 | 339 (28·8%) | 100 124 (28·1%) |
|  | **LVEF** | **≥50 (ref)** |  |  |  |  |  |  | 237 (20·1%) | 56 000 (15·7%) |
|  |  | **30–49** | 0·0209 | –0·0686 | 0·1104 | 0·0272 | 0·8 | 0·44 | 858 (72·8%) | 277 715 (78·1%) |
|  |  | **<30** | 0·0556 | –0·1027 | 0·2139 | 0·0481 | 1·2 | 0·25 | 83 (7·0%) | 22 100 (6·2%) |
|  | **eGFR** | **<60** | –0·0068 | –0·1199 | 0·1063 | 0·0344 | –0·2 | 0·84 | 147 (12·5%) | 43 441 (12·2%) |
|  | **STEMI** | **STEMI** | –0·0123 | –0·0936 | 0·0691 | 0·0247 | –0·5 | 0·62 | 848 (72·0%) | 257 491 (72·4%) |
|  | **Revascularization** | **Yes** | –0·0193 | –0·0632 | 0·0245 | 0·0133 | –1·5 | 0·15 | 95 (8·1%) | 19 625 (5·5%) |
|  | **# medications** | **>12** | 0·0121 | –0·0626 | 0·0869 | 0·0227 | 0·5 | 0·59 | 567 (48·1%) | 171 113 (48·1%) |
|  | **NYHA** | **No disease (ref)** |  |  |  |  |  |  | 711 (60·4%) | 163929 (46·1%) |
|  |  | **No symptoms** | 0·0127 | –0·0127 | 0·038 | 0·0077 | 1·6 | 0·1 | 554 (47·0%) | 118 696 (33·4%) |
|  |  | **Mild symptoms** | 0·0111 | –0·0209 | 0·0432 | 0·0097 | 1·1 | 0·25 | 296 (25·1%) | 65 461 (18·4%) |
|  |  | **Marked/Severe** | –0·0582 | –0·1341 | 0·0176 | 0·023 | –2·5 | 0·011 | 55 (4·7%) | 7729 (2·2%) |
|  | **CCS Angina grade** | **Asymptomatic (ref)** |  |  |  |  |  |  | 1009 (85·7%) | 275 533 (77·4%) |
|  |  | **Strenuous exertion** | –0·0017 | –0·0321 | 0·0288 | 0·0092 | –0·2 | 0·86 | 317 (26·9%) | 63 565 (17·9%) |
|  |  | **Moderate exertion** | –0·1075 | –0·1754 | –0·0396 | 0·0206 | –5·2 | 1·9e-07 | 63 (5·3%) | 12 179 (3·4%) |
|  |  | **Mild exertion/At rest** | –0·1999 | –0·2963 | –0·1036 | 0·0293 | –6·8 | 8·6e-12 | 23 (2·0%) | 4538 (1·3%) |
| Calendar features | **Days from start** | **≤30 (ref)** |  |  |  |  |  |  | 1178 (100·0%) | 33 852 (9·5%) |
|  |  | **31–60** | –0·0404 | –0·0611 | –0·0196 | 0·0063 | –6·4 | 1·5e-10 | 1162 (98·6%) | 33 234 (9·3%) |
|  |  | **61–90** | –0·0245 | –0·0457 | –0·0034 | 0·0064 | –3·8 | 0·00013 | 1120 (95·1%) | 31 916 (9·0%) |
|  |  | **91–120** | –0·0064 | –0·0282 | 0·0154 | 0·0066 | –1 | 0·33 | 1074 (91·2%) | 29 408 (8·3%) |
|  |  | **121–150** | 0·015 | –0·0076 | 0·0376 | 0·0069 | 2·2 | 0·029 | 969 (82·3%) | 26 771 (7·5%) |
|  |  | **151–200** | 0·0307 | 0·0096 | 0·0518 | 0·0064 | 4·8 | 1·7e-06 | 893 (75·8%) | 39 896 (11·2%) |
|  |  | **201–250** | 0·025 | 0·0026 | 0·0473 | 0·0068 | 3·7 | 0·00024 | 782 (66·4%) | 34 142 (9·6%) |
|  |  | **251–300** | 0·0273 | 0·0035 | 0·051 | 0·0072 | 3·8 | 0·00016 | 672 (57·0%) | 30 133 (8·5%) |
|  |  | **301–350** | 0·05 | 0·0245 | 0·0755 | 0·0077 | 6·4 | 1·1e-10 | 597 (50·7%) | 25 566 (7·2%) |
|  |  | **351–400** | 0·0477 | 0·0197 | 0·0758 | 0·0085 | 5·6 | 2·1e-08 | 472 (40·1%) | 19 388 (5·4%) |
|  |  | **401–450** | 0·0575 | 0·027 | 0·0879 | 0·0092 | 6·2 | 5·2e-10 | 363 (30·8%) | 15 943 (4·5%) |
|  |  | **451–500** | 0·0633 | 0·03 | 0·0967 | 0·0101 | 6·2 | 4·3e-10 | 298 (25·3%) | 12 645 (3·6%) |
|  |  | **>500** | 0·0603 | 0·0281 | 0·0924 | 0·0098 | 6·2 | 6·9e-10 | 225 (19·1%) | 22 921 (6·4%) |
|  | **Month** | **January (ref)** |  |  |  |  |  |  | 1014 (86·1%) | 37 453 (10·5%) |
|  |  | **February** | –0·0271 | –0·0472 | –0·0071 | 0·0061 | –4·4 | 8·7e-06 | 1067 (90·6%) | 36 407 (10·2%) |
|  |  | **March** | –0·0241 | –0·0524 | 0·0042 | 0·0086 | –2·8 | 0·0051 | 1075 (91·3%) | 40 944 (11·5%) |
|  |  | **April** | –0·0127 | –0·0406 | 0·0153 | 0·0085 | –1·5 | 0·14 | 1004 (85·2%) | 31 118 (8·7%) |
|  |  | **May** | 0·0142 | –0·0143 | 0·0427 | 0·0087 | 1·6 | 0·1 | 841 (71·4%) | 22 497 (6·3%) |
|  |  | **June** | 0·0249 | –0·0044 | 0·0542 | 0·0089 | 2·8 | 0·0052 | 668 (56·7%) | 19 525 (5·5%) |
|  |  | **July** | 0·0075 | –0·0215 | 0·0365 | 0·0088 | 0·8 | 0·4 | 696 (59·1%) | 22 229 (6·2%) |
|  |  | **August** | 0·0077 | –0·0211 | 0·0365 | 0·0088 | 0·9 | 0·38 | 743 (63·1%) | 23 853 (6·7%) |
|  |  | **September** | –0·0071 | –0·0355 | 0·0214 | 0·0086 | –0·8 | 0·41 | 813 (69·0%) | 25 996 (7·3%) |
|  |  | **October** | –0·0036 | –0·032 | 0·0248 | 0·0086 | –0·4 | 0·67 | 861 (73·1%) | 29 651 (8·3%) |
|  |  | **November** | –0·0154 | –0·0437 | 0·0129 | 0·0086 | –1·8 | 0·074 | 917 (77·8%) | 31 145 (8·8%) |
|  |  | **December** | 0·0304 | 0·0024 | 0·0584 | 0·0085 | 3·6 | 0·00035 | 973 (82·6%) | 34 997 (9·8%) |
|  | **Days of the week** | **Monday** | 0·2553 | 0·2386 | 0·2721 | 0·0051 | 50·2 | 0·0 | 1178 (100·0%) | 50 927 (14·3%) |
|  |  | **Tuesday** | 0·0306 | 0·014 | 0·0472 | 0·005 | 6·1 | 1·2e-09 | 1178 (100·0%) | 50 957 (14·3%) |
|  |  | **Wednesday (ref)** |  |  |  |  |  |  | 1178 (100·0%) | 50 854 (14·3%) |
|  |  | **Thursday** | 0·0124 | –0·0042 | 0·029 | 0·005 | 2·5 | 0·014 | 1178 (100·0%) | 50 831 (14·3%) |
|  |  | **Friday** | 0·0351 | 0·0185 | 0·0517 | 0·0051 | 7 | 3·6e-12 | 1178 (100·0%) | 50 744 (14·3%) |
|  |  | **Saturday** | 0·2016 | 0·185 | 0·2182 | 0·005 | 39·9 | 0·0 | 1178 (100·0%) | 50 738 (14·3%) |
|  |  | **Sunday** | 0·1414 | 0·1247 | 0·1581 | 0·0051 | 27·9 | 2·9e-171 | 1178 (100·0%) | 50 764 (14·3%) |
|  | **Holiday** | **Holiday** | 0·0098 | –0·0143 | 0·0338 | 0·0073 | 1·3 | 0·18 | 1141 (96·9%) | 13 890 (3·9%) |
|  | **Study Closeout** | **Yes** | 0·0248 | –0·0037 | 0·0533 | 0·0087 | 2·9 | 0·0042 | 1018 (86·4%) | 50 175 (14·1%) |
| Adverse events | **Hospitalized or discontinued** | **No AE (ref)** |  |  |  |  |  |  | 1166 (99·0%) | 334 707 (94·1%) |
|  |  | **Neither** | 0·0699 | 0·0241 | 0·1157 | 0·0139 | 5 | 5·2e-07 | 132 (11·2%) | 15 464 (4·3%) |
|  |  | **Discontinued only** | 0·0488 | –0·1373 | 0·235 | 0·0566 | 0·9 | 0·39 | 18 (1·5%) | 522 (0·1%) |
|  |  | **Hospitalized only** | 0·2234 | 0·1614 | 0·2853 | 0·0188 | 11·9 | 1·9e-32 | 156 (13·2%) | 3804 (1·1%) |
|  |  | **Both** | 0·1861 | 0·074 | 0·2982 | 0·0341 | 5·5 | 4·7e-08 | 48 (4·1%) | 1318 (0·4%) |
|  | **7 days after AE** | **No AE (ref)** |  |  |  |  |  |  | 1178 (100·0%) | 353 798 (99·4%) |
|  |  | **Not hospitalized or discontinued** | –0·0109 | –0·1174 | 0·0956 | 0·0324 | –0·3 | 0·74 | 85 (7·2%) | 639 (0·2%) |
|  |  | **Not hospitalized but discontinued** | 0·3527 | –0·1375 | 0·8429 | 0·149 | 2·4 | 0·018 | 4 (0·3%) | 30 (0·0%) |
|  |  | **Hospitalized, not discontinued** | 0·0481 | –0·0328 | 0·1289 | 0·0246 | 2 | 0·05 | 135 (11·5%) | 1137 (0·3%) |
|  |  | **Hospitalized and discontinued** | 0·1527 | –0·0333 | 0·3388 | 0·0565 | 2·7 | 0·0069 | 31 (2·6%) | 211 (0·1%) |
| Behavioural features | **Mobile application use** | **Yes** | 0·0491 | –0·0162 | 0·1144 | 0·0198 | 2·5 | 0·013 | 312 (26·5%) | 1689 (0·5%) |
|  | **7 days after mobile application use** | **Yes** | 0·0048 | –0·0217 | 0·0312 | 0·008 | 0·6 | 0·55 | 327 (27·8%) | 12 175 (3·4%) |
|  | **Off-schedule use in last 7 days** | **Yes** | 0·0653 | 0·047 | 0·0836 | 0·0056 | 11·7 | 9·3e-32 | 1114 (94·6%) | 24 105 (6·8%) |
|  | **Average time in last 7 days** | **0–4** | 0·0765 | –0·0015 | 0·1545 | 0·0237 | 3·2 | 0·0013 | 57 (4·8%) | 1914 (0·5%) |
|  |  | **4–8** | –0·0398 | –0·0548 | –0·0249 | 0·0045 | –8·8 | 2e-18 | 835 (70·9%) | 126 134 (35·4%) |
|  |  | **8–12 (ref)** |  |  |  |  |  |  | 1032 (87·6%) | 195 019 (54·8%) |
|  |  | **12–16** | 0·305 | 0·2564 | 0·3537 | 0·0148 | 20·6 | 1·4e-94 | 215 (18·3%) | 7414 (2·1%) |
|  |  | **16–20** | 0·1075 | 0·0462 | 0·1688 | 0·0186 | 5·8 | 7·9e-09 | 169 (14·3%) | 13 923 (3·9%) |
|  |  | **20–24** | 0·0199 | –0·0565 | 0·0963 | 0·0232 | 0·9 | 0·39 | 87 (7·4%) | 11 411 (3·2%) |
| Disruptions | **7 days after data gap** | **Yes** | 0·2459 | 0·2031 | 0·2887 | 0·013 | 18·9 | 1·1e-79 | 400 (34·0%) | 4123 (1·2%) |
|  | **7 days after a holiday** | **Yes** | 0·023 | 0·0086 | 0·0374 | 0·0044 | 5·2 | 1·5e-07 | 1142 (96·9%) | 46 079 (13·0%) |
|  | **After a missed dose** | **No recent non–adherence (ref)** |  |  |  |  |  |  | 1178 (100·0%) | 318 968 (89·6%) |
|  |  | **1 day after ≤3 missed** | 0·2915 | 0·2569 | 0·326 | 0·0105 | 27·8 | 1·6e-169 | 970 (82·3%) | 6192 (1·7%) |
|  |  | **2 days after ≤3 missed** | 0·1192 | 0·0829 | 0·1555 | 0·011 | 10·8 | 3e-27 | 967 (82·1%) | 5558 (1·6%) |
|  |  | **3 days after ≤3 missed** | 0·0454 | 0·0079 | 0·083 | 0·0114 | 4 | 6·8e-05 | 967 (82·1%) | 5159 (1·4%) |
|  |  | **4 days after ≤3 missed** | 0·0173 | –0·0213 | 0·0559 | 0·0117 | 1·5 | 0·14 | 967 (82·1%) | 4856 (1·4%) |
|  |  | **5 days after ≤3 missed** | –0·0022 | –0·0417 | 0·0374 | 0·012 | –0·2 | 0·86 | 965 (81·9%) | 4602 (1·3%) |
|  |  | **6 days after ≤3 missed** | 0·0261 | –0·0145 | 0·0667 | 0·0123 | 2·1 | 0·035 | 965 (81·9%) | 4357 (1·2%) |
|  |  | **7 days after ≤3 missed** | 0·031 | –0·0107 | 0·0727 | 0·0127 | 2·4 | 0·014 | 960 (81·5%) | 4117 (1·2%) |
|  |  | **1 day after >3 missed** | 0·6429 | 0·5174 | 0·7683 | 0·0381 | 16·9 | 9·2e-64 | 241 (20·5%) | 450 (0·1%) |
|  |  | **2 days after >3 missed** | 0·4189 | 0·2721 | 0·5657 | 0·0446 | 9·4 | 6·1e-21 | 218 (18·5%) | 327 (0·1%) |
|  |  | **3 days after >3 missed** | 0·1449 | –0·0131 | 0·303 | 0·048 | 3 | 0·0026 | 208 (17·7%) | 282 (0·1%) |
|  |  | **4 days after >3 missed** | 0·0848 | –0·0805 | 0·25 | 0·0502 | 1·7 | 0·091 | 200 (17·0%) | 258 (0·1%) |
|  |  | **5 days after >3 missed** | 0·0832 | –0·0877 | 0·2541 | 0·0519 | 1·6 | 0·11 | 191 (16·2%) | 241 (0·1%) |
|  |  | **6 days after >3 missed** | 0·0695 | –0·1062 | 0·2452 | 0·0534 | 1·3 | 0·19 | 181 (15·4%) | 228 (0·1%) |
|  |  | **7 days after >3 missed** | 0·0151 | –0·1637 | 0·1939 | 0·0543 | 0·3 | 0·78 | 178 (15·1%) | 220 (0·1%) |
| Other | **Lost >5% weight** | **Yes** | –0·0472 | –0·1287 | 0·0343 | 0·0248 | –1·9 | 0·056 | 308 (26·1%) | 96 038 (27·0%) |
|  | **COVID-19 restrictions** | **Yes** | –0·006 | –0·0333 | 0·0212 | 0·0083 | –0·7 | 0·47 | 1061 (90·1%) | 252 525 (71·0%) |
|  | **Intercept** |  | –0·4139 | –0·5483 | –0·2795 | 0·0408 | –10·1 | 3·8e-24 | 1178 (100%) | 355 815 (100%) |

Results of the linear mixed effects regression model fit on low-initial dose consistency subset of patients of the Box–Cox-transformed dose consistency (the absolute value of adherence time differences on consecutive days). Patients included in this dataset had below-median average dose consistency between days 7 and 35 after randomization. Only patients who had at least 10 days of data during that period were included in this analysis. The coefficients are represented by the blue colour in corresponding Supplementary Figure 8. Variables with multiple values are dichotomized and reference values are indicated by (ref). Error bars represent 99·95% CIs.

AE=adverse event; BMI=body mass index; CCS=Canadian Cardiovascular Society; CI=confidence interval; eGFR=estimated glomerular filtration rate; LVEF=left ventricular ejection fraction; NYHA=New York Heart Association; SE=standard error; STEMI=ST-elevation myocardial infarction.

### Supplementary Table 12. Results of Box–Cox-transformed dose consistency model for patients with high initial dose consistency

| Group | Feature | Value | Estimate | 0·05 % | 99·95 % | SE | z value | Pr(> \|z\|) | No. patients | No. observations |
| --- | --- | --- | --- | --- | --- | --- | --- | --- | --- | --- |
| Patient characteristics | **Country** | **Sweden** | –0·0228 | –0·0931 | 0·0476 | 0·0214 | –1·1 | 0·29 | 221 (18·8%) | 70 037 (22·1%) |
|  | **Sex** | **Female** | –0·0052 | –0·0746 | 0·0643 | 0·0211 | –0·2 | 0·81 | 203 (17·2%) | 53 746 (16·9%) |
|  | **Age** | **<40** | –0·0237 | –0·179 | 0·1316 | 0·0472 | –0·5 | 0·61 | 39 (3·3%) | 8196 (2·6%) |
|  |  | **40–50** | –0·0142 | –0·1006 | 0·0722 | 0·0263 | –0·5 | 0·59 | 152 (12·9%) | 38 890 (12·3%) |
|  |  | **50–60** | 0·006 | –0·057 | 0·069 | 0·0191 | 0·3 | 0·75 | 413 (35·1%) | 104 881 (33·1%) |
|  |  | **60–70 (ref)** |  |  |  |  |  |  | 363 (30·8%) | 105 100 (33·1%) |
|  |  | **70–80** | –0·0399 | –0·1211 | 0·0414 | 0·0247 | –1·6 | 0·11 | 186 (15·8%) | 53 340 (16·8%) |
|  |  | **>80** | 0·0194 | –0·1706 | 0·2093 | 0·0577 | 0·3 | 0·74 | 24 (2·0%) | 6708 (2·1%) |
|  | **Race** | **White (ref)** |  |  |  |  |  |  | 1107 (94·1%) | 301 872 (95·2%) |
|  |  | **Asian** | 0·0609 | –0·0695 | 0·1914 | 0·0397 | 1·5 | 0·12 | 51 (4·3%) | 11 610 (3·7%) |
|  |  | **Other** | 0·09 | –0·1193 | 0·2994 | 0·0636 | 1·4 | 0·16 | 19 (1·6%) | 3633 (1·1%) |
|  | **Arm** | **Dapagliflozin 10 mg** | –0·0007 | –0·0523 | 0·0508 | 0·0157 | 0 | 0·96 | 574 (48·8%) | 157 990 (49·8%) |
|  | **Smoking status** | **Never (ref)** |  |  |  |  |  |  | 452 (38·4%) | 128 615 (40·6%) |
|  |  | **Former** | 0·0419 | –0·0229 | 0·1067 | 0·0197 | 2·1 | 0·033 | 303 (25·7%) | 82 521 (26·0%) |
|  |  | **Current** | 0·0824 | 0·0212 | 0·1436 | 0·0186 | 4·4 | 9·3e-06 | 422 (35·9%) | 105 979 (33·4%) |
|  | **Hypertension history** | **Yes** | 0·0184 | –0·0377 | 0·0744 | 0·017 | 1·1 | 0·28 | 420 (35·7%) | 111 483 (35·2%) |
| Health status | **Systolic pressure** | **≥130** | 0·0285 | –0·042 | 0·099 | 0·0214 | 1·3 | 0·18 | 259 (22·0%) | 71 354 (22·5%) |
|  | **Diastolic pressure** | **≥80** | 0·0123 | –0·0532 | 0·0778 | 0·0199 | 0·6 | 0·54 | 321 (27·3%) | 88 331 (27·9%) |
|  | **BMI** | **≥30** | 0·0313 | –0·0234 | 0·086 | 0·0166 | 1·9 | 0·059 | 414 (35·2%) | 108 576 (34·2%) |
|  | **LVEF** | **≥50 (ref)** |  |  |  |  |  |  | 255 (21·7%) | 56 461 (17·8%) |
|  |  | **30–49** | –0·05 | –0·1139 | 0·0139 | 0·0194 | –2·6 | 0·01 | 847 (72·0%) | 242 583 (76·5%) |
|  |  | **<30** | –0·0467 | –0·1663 | 0·0728 | 0·0363 | –1·3 | 0·2 | 75 (6·4%) | 18 071 (5·7%) |
|  | **eGFR** | **<60** | 0·0054 | –0·0941 | 0·1049 | 0·0302 | 0·2 | 0·86 | 88 (7·5%) | 26 053 (8·2%) |
|  | **STEMI** | **STEMI** | –0·0187 | –0·0794 | 0·0419 | 0·0184 | –1 | 0·31 | 888 (75·4%) | 239 678 (75·6%) |
|  | **Revascularization** | **Yes** | –0·02 | –0·075 | 0·0351 | 0·0167 | –1·2 | 0·23 | 71 (6·0%) | 15 996 (5·0%) |
|  | **# medications** | **>12** | –0·0046 | –0·0588 | 0·0496 | 0·0165 | –0·3 | 0·78 | 575 (48·9%) | 161 108 (50·8%) |
|  | **NYHA** | **No disease (ref)** |  |  |  |  |  |  | 653 (55·5%) | 143 325 (45·2%) |
|  |  | **No symptoms** | 0·016 | –0·0126 | 0·0446 | 0·0087 | 1·8 | 0·066 | 551 (46·8%) | 109 974 (34·7%) |
|  |  | **Mild symptoms** | 0·0198 | –0·0151 | 0·0547 | 0·0106 | 1·9 | 0·062 | 291 (24·7%) | 53 946 (17·0%) |
|  |  | **Marked/Severe** | 0·087 | 0·0182 | 0·1558 | 0·0209 | 4·2 | 3·2e-05 | 64 (5·4%) | 9870 (3·1%) |
|  | **CCS Angina grade** | **Asymptomatic (ref)** |  |  |  |  |  |  | 973 (82·7%) | 238 245 (75·1%) |
|  |  | **Strenuous exertion** | 0·028 | –0·0048 | 0·0607 | 0·01 | 2·8 | 0·005 | 314 (26·7%) | 60 921 (19·2%) |
|  |  | **Moderate exertion** | 0·0107 | –0·0487 | 0·0701 | 0·0181 | 0·6 | 0·55 | 73 (6·2%) | 14 557 (4·6%) |
|  |  | **Mild exertion/At rest** | –0·029 | –0·1448 | 0·0868 | 0·0352 | –0·8 | 0·41 | 24 (2·0%) | 3392 (1·1%) |
| Calendar features | **Days from start** | **≤30 (ref)** |  |  |  |  |  |  | 1175 (99·8%) | 31 985 (10·1%) |
|  |  | **31–60** | 0·0362 | 0·0112 | 0·0612 | 0·0076 | 4·8 | 1·9e-06 | 1158 (98·4%) | 31 190 (9·8%) |
|  |  | **61–90** | 0·0719 | 0·0462 | 0·0976 | 0·0078 | 9·2 | 3·2e-20 | 1131 (96·1%) | 29 566 (9·3%) |
|  |  | **91–120** | 0·1178 | 0·0911 | 0·1444 | 0·0081 | 14·5 | 6·2e-48 | 1064 (90·4%) | 26 517 (8·4%) |
|  |  | **121–150** | 0·1125 | 0·0846 | 0·1404 | 0·0085 | 13·3 | 3·5e-40 | 929 (78·9%) | 23 305 (7·3%) |
|  |  | **151–200** | 0·1331 | 0·1072 | 0·1589 | 0·0079 | 16·9 | 2e-64 | 850 (72·2%) | 34 212 (10·8%) |
|  |  | **201–250** | 0·1349 | 0·1075 | 0·1622 | 0·0083 | 16·2 | 4·4e-59 | 719 (61·1%) | 29 060 (9·2%) |
|  |  | **251–300** | 0·1423 | 0·1134 | 0·1713 | 0·0088 | 16·2 | 6·4e-59 | 626 (53·2%) | 25 906 (8·2%) |
|  |  | **301–350** | 0·1508 | 0·1198 | 0·1819 | 0·0094 | 16 | 1·6e-57 | 563 (47·8%) | 21 874 (6·9%) |
|  |  | **351–400** | 0·1611 | 0·1276 | 0·1945 | 0·0102 | 15·9 | 1·4e-56 | 457 (38·8%) | 18 106 (5·7%) |
|  |  | **401–450** | 0·1609 | 0·1246 | 0·1972 | 0·011 | 14·6 | 3·7e-48 | 367 (31·2%) | 15 358 (4·8%) |
|  |  | **451–500** | 0·1705 | 0·1305 | 0·2106 | 0·0122 | 14 | 1·5e-44 | 301 (25·6%) | 12 392 (3·9%) |
|  |  | **>500** | 0·1707 | 0·1304 | 0·211 | 0·0122 | 13·9 | 3·9e-44 | 241 (20·5%) | 17 644 (5·6%) |
|  | **Month** | **January (ref)** |  |  |  |  |  |  | 985 (83·7%) | 33 807 (10·7%) |
|  |  | **February** | 0·0099 | –0·0149 | 0·0347 | 0·0075 | 1·3 | 0·19 | 1030 (87·5%) | 32 736 (10·3%) |
|  |  | **March** | –0·0179 | –0·0521 | 0·0163 | 0·0104 | –1·7 | 0·085 | 1045 (88·8%) | 36 713 (11·6%) |
|  |  | **April** | –0·0004 | –0·0343 | 0·0335 | 0·0103 | 0 | 0·97 | 1007 (85·6%) | 28 654 (9·0%) |
|  |  | **May** | 0·0298 | –0·0048 | 0·0644 | 0·0105 | 2·8 | 0·0046 | 833 (70·8%) | 20 693 (6·5%) |
|  |  | **June** | 0·0347 | –0·0012 | 0·0707 | 0·0109 | 3·2 | 0·0015 | 662 (56·2%) | 17 110 (5·4%) |
|  |  | **July** | 0·0306 | –0·0051 | 0·0663 | 0·0109 | 2·8 | 0·0048 | 662 (56·2%) | 19 061 (6·0%) |
|  |  | **August** | –0·0039 | –0·0391 | 0·0314 | 0·0107 | –0·4 | 0·72 | 700 (59·5%) | 20 720 (6·5%) |
|  |  | **September** | –0·013 | –0·0479 | 0·0219 | 0·0106 | –1·2 | 0·22 | 759 (64·5%) | 21 921 (6·9%) |
|  |  | **October** | –0·0017 | –0·0363 | 0·0328 | 0·0105 | –0·2 | 0·87 | 814 (69·2%) | 25 810 (8·1%) |
|  |  | **November** | –0·0137 | –0·0479 | 0·0205 | 0·0104 | –1·3 | 0·19 | 878 (74·6%) | 28 088 (8·9%) |
|  |  | **December** | 0·0197 | –0·0139 | 0·0534 | 0·0102 | 1·9 | 0·054 | 932 (79·2%) | 31 802 (10·0%) |
|  | **Days of the week** | **Monday** | 0·3406 | 0·3197 | 0·3614 | 0·0063 | 53·8 | 0·0 | 1176 (99·9%) | 45 554 (14·4%) |
|  |  | **Tuesday** | 0·0439 | 0·0233 | 0·0645 | 0·0063 | 7 | 2·3e-12 | 1173 (99·7%) | 45 537 (14·4%) |
|  |  | **Wednesday (ref)** |  |  |  |  |  |  | 1175 (99·8%) | 45 419 (14·3%) |
|  |  | **Thursday** | 0·0081 | –0·0125 | 0·0287 | 0·0063 | 1·3 | 0·2 | 1175 (99·8%) | 45 449 (14·3%) |
|  |  | **Friday** | 0·0461 | 0·0254 | 0·0667 | 0·0063 | 7·3 | 2·1e-13 | 1176 (99·9%) | 45 305 (14·3%) |
|  |  | **Saturday** | 0·2965 | 0·2758 | 0·3172 | 0·0063 | 47·1 | 0·0 | 1174 (99·7%) | 44 844 (14·1%) |
|  |  | **Sunday** | 0·2013 | 0·1806 | 0·2221 | 0·0063 | 31·9 | 2·4e-223 | 1173 (99·7%) | 45 007 (14·2%) |
|  | **Holiday** | **Holiday** | 0·0036 | –0·0264 | 0·0336 | 0·0091 | 0·4 | 0·69 | 1131 (96·1%) | 12 327 (3·9%) |
|  | **Study Closeout** | **Yes** | 0·0173 | –0·0174 | 0·0519 | 0·0105 | 1·6 | 0·1 | 992 (84·3%) | 46 013 (14·5%) |
| Adverse events | **Hospitalized or discontinued** | **No AE (ref)** |  |  |  |  |  |  | 1167 (99·2%) | 302 600 (95·4%) |
|  |  | **Neither** | 0·1062 | 0·0533 | 0·1592 | 0·0161 | 6·6 | 4e-11 | 138 (11·7%) | 10 779 (3·4%) |
|  |  | **Discontinued only** | 0·1176 | –0·0375 | 0·2728 | 0·0472 | 2·5 | 0·013 | 19 (1·6%) | 604 (0·2%) |
|  |  | **Hospitalized only** | 0·0297 | –0·0523 | 0·1116 | 0·0249 | 1·2 | 0·23 | 126 (10·7%) | 2256 (0·7%) |
|  |  | **Both** | 0·0522 | –0·0848 | 0·1893 | 0·0416 | 1·3 | 0·21 | 46 (3·9%) | 876 (0·3%) |
|  | **7 days after AE** | **No AE (ref)** |  |  |  |  |  |  | 1177 (100·0%) | 315 308 (99·4%) |
|  |  | **Not hospitalized or discontinued** | –0·006 | –0·1259 | 0·1138 | 0·0364 | –0·2 | 0·87 | 90 (7·6%) | 699 (0·2%) |
|  |  | **Not hospitalized but discontinued** | 0·3151 | –0·1908 | 0·821 | 0·1538 | 2 | 0·04 | 7 (0·6%) | 39 (0·0%) |
|  |  | **Hospitalized, not discontinued** | –0·0106 | –0·1167 | 0·0954 | 0·0322 | –0·3 | 0·74 | 124 (10·5%) | 890 (0·3%) |
|  |  | **Hospitalized and discontinued** | 0·0012 | –0·2343 | 0·2366 | 0·0716 | 0 | 0·99 | 34 (2·9%) | 179 (0·1%) |
| Behavioural features | **Mobile application use** | **Yes** | 0·0789 | –0·0045 | 0·1622 | 0·0253 | 3·1 | 0·0018 | 325 (27·6%) | 1422 (0·4%) |
|  | **7 days after mobile application use** | **Yes** | 0·009 | –0·0246 | 0·0425 | 0·0102 | 0·9 | 0·38 | 345 (29·3%) | 10 145 (3·2%) |
|  | **Off-schedule use in last 7 days** | **Yes** | 0·0689 | 0·0504 | 0·0875 | 0·0056 | 12·2 | 1·8e-34 | 1100 (93·5%) | 36 008 (11·4%) |
|  | **Average time in last 7 days** | **0–4** | 0·0726 | –0·004 | 0·1492 | 0·0233 | 3·1 | 0·0018 | 122 (10·4%) | 2084 (0·7%) |
|  |  | **4–8** | –0·1143 | –0·1327 | –0·0959 | 0·0056 | –20·4 | 1·1e-92 | 766 (65·1%) | 72 852 (23·0%) |
|  |  | **8–12 (ref)** |  |  |  |  |  |  | 1067 (90·7%) | 171 100 (54·0%) |
|  |  | **12–16** | 0·2441 | 0·2201 | 0·2682 | 0·0073 | 33·4 | 6·2e-245 | 731 (62·1%) | 35 873 (11·3%) |
|  |  | **16–20** | 0·1513 | 0·1156 | 0·1869 | 0·0108 | 14 | 2·5e-44 | 429 (36·4%) | 21 225 (6·7%) |
|  |  | **20–24** | –0·1138 | –0·1602 | –0·0673 | 0·0141 | –8·1 | 8·2e-16 | 190 (16·1%) | 13 981 (4·4%) |
| Disruptions | **7 days after data gap** | **Yes** | 0·219 | 0·177 | 0·261 | 0·0128 | 17·2 | 5·4e-66 | 518 (44·0%) | 6115 (1·9%) |
|  | **7 days after a holiday** | **Yes** | 0·029 | 0·011 | 0·0471 | 0·0055 | 5·3 | 1·2e-07 | 1143 (97·1%) | 40 452 (12·8%) |
|  | **After a missed dose** | **No recent non–adherence (ref)** |  |  |  |  |  |  | 1171 (99·5%) | 235 200 (74·2%) |
|  |  | **1 day after ≤3 missed** | 0·3753 | 0·349 | 0·4016 | 0·008 | 47 | 0·0 | 1105 (93·9%) | 16 618 (5·2%) |
|  |  | **2 days after ≤3 missed** | 0·0946 | 0·0663 | 0·1229 | 0·0086 | 11 | 3·3e-28 | 1095 (93·0%) | 13 798 (4·4%) |
|  |  | **3 days after ≤3 missed** | 0·0414 | 0·0113 | 0·0714 | 0·0091 | 4·5 | 5·9e-06 | 1090 (92·6%) | 11 870 (3·7%) |
|  |  | **4 days after ≤3 missed** | 0·0154 | –0·0164 | 0·0471 | 0·0096 | 1·6 | 0·11 | 1080 (91·8%) | 10 434 (3·3%) |
|  |  | **5 days after ≤3 missed** | 0·007 | –0·0264 | 0·0403 | 0·0101 | 0·7 | 0·49 | 1074 (91·2%) | 9334 (2·9%) |
|  |  | **6 days after ≤3 missed** | 0·0147 | –0·0203 | 0·0496 | 0·0106 | 1·4 | 0·17 | 1067 (90·7%) | 8390 (2·6%) |
|  |  | **7 days after ≤3 missed** | 0·0355 | –0·0014 | 0·0723 | 0·0112 | 3·2 | 0·0015 | 1059 (90·0%) | 7458 (2·4%) |
|  |  | **1 day after >3 missed** | 0·5631 | 0·4764 | 0·6497 | 0·0263 | 21·4 | 1·7e-101 | 461 (39·2%) | 1426 (0·4%) |
|  |  | **2 days after >3 missed** | 0·2761 | 0·1575 | 0·3947 | 0·036 | 7·7 | 1·9e-14 | 399 (33·9%) | 698 (0·2%) |
|  |  | **3 days after >3 missed** | 0·05 | –0·0886 | 0·1886 | 0·0421 | 1·2 | 0·24 | 346 (29·4%) | 508 (0·2%) |
|  |  | **4 days after >3 missed** | 0·0873 | –0·0655 | 0·2401 | 0·0464 | 1·9 | 0·06 | 305 (25·9%) | 417 (0·1%) |
|  |  | **5 days after >3 missed** | –0·0075 | –0·1722 | 0·1572 | 0·0501 | –0·1 | 0·88 | 272 (23·1%) | 358 (0·1%) |
|  |  | **6 days after >3 missed** | –0·0559 | –0·2314 | 0·1196 | 0·0533 | –1 | 0·29 | 242 (20·6%) | 315 (0·1%) |
|  |  | **7 days after >3 missed** | 0·0331 | –0·1495 | 0·2156 | 0·0555 | 0·6 | 0·55 | 230 (19·5%) | 291 (0·1%) |
| Other | **Lost >5% weight** | **Yes** | –0·0257 | –0·0876 | 0·0363 | 0·0188 | –1·4 | 0·17 | 262 (22·3%) | 67 594 (21·3%) |
|  | **COVID-19 restrictions** | **Yes** | 0·0065 | –0·0265 | 0·0396 | 0·01 | 0·6 | 0·52 | 1032 (87·7%) | 224 665 (70·8%) |
|  | **Intercept** |  | 0·0546 | –0·0479 | 0·1572 | 0·0312 | 1·8 | 0·08 | 1177 (100%) | 317 115 (100%) |

Results of the linear mixed effects regression model fit on high-initial dose consistency subset of patients of the Box–Cox-transformed dose consistency (the absolute value of adherence time differences on consecutive days). Patients included in this dataset had above-median average dose consistency between days 7 and 35 after randomization. Only patients who had at least 10 days of data during that period were included in this analysis. The coefficients are represented by the red colour in corresponding Supplementary Figure 8. Variables with multiple values are dichotomized and reference values are indicated by (ref). Error bars represent 99·95% CIs.

AE=adverse event; BMI=body mass index; CCS=Canadian Cardiovascular Society; CI=confidence interval; eGFR=estimated glomerular filtration rate; LVEF=left ventricular ejection fraction; NYHA=New York Heart Association; SE=standard error; STEMI=ST-elevation myocardial infarction.

### Supplementary Table 13. Example of calculation of average time variance in last 7 days.

| Date | 12/1/2021 | 12/2/2021 | 12/3/2021 | 12/4/2021 | 12/5/2021 | 12/6/2021 | 12/7/2021 | 12/8/2021 | 12/9/2021 |
| --- | --- | --- | --- | --- | --- | --- | --- | --- | --- |
| Cap-opening time (h:m:s) | 12:05:01 | 12:01:01 | 12:30:29 | 11:00:00 | 13:20:10 | Dose missed | 12:10:13 | 12:10:10 | 12:12:00 |
| Dose consistency [sec] | - | 240 | 1768 | 5429 | 8410 | - | 4197 | 3 | 110 |
| Average cap-opening time in the previous 7 days | - | - | - | - | - | - | - | - | 12:11:01 |
| Average time variance in last 7 days | - | - | - | - | - | - | - | - | 25min 19sec (1519.4 sec) |

## Supplementary References

1.	Lumley T, Diehr P, Emerson S, Chen L. The importance of the normality assumption in large public health data sets. *Annu Rev Public Health* 2002; **23**: 151–69.

2.	Chen T, Guestrin C. XGBoost: A Scalable Tree Boosting System. Proceedings of the 22^nd^ ACM SIGKDD International Conference on Knowledge Discovery and Data Mining. San Francisco, California, USA: Association for Computing Machinery; 2016; 785–94.
